# SPRED2 Negatively Regulates CD8^+^ T Cell‐Mediated Antitumor Immunity in Breast Cancer

**DOI:** 10.1002/advs.77909

**Published:** 2026-09-27

**Authors:** Miao Tian, Teizo Yoshimura, Kun Zhao, Chunning Li, Tong Gao, Ziyi Wang, Masayoshi Fujisawa, Toshiaki Ohara, Akihiro Matsukawa

**Affiliations:** ^1^ Department of Pathology and Experimental Medicine, Graduate School of Medicine, Dentistry and Pharmaceutical Sciences Okayama University Okayama Japan; ^2^ Department of Oral Rehabilitation and Regenerative Medicine, Graduate School of Medicine, Dentistry and Pharmaceutical Sciences Okayama University Okayama Japan; ^3^ Department of Advanced International and Information Dentistry Faculty of Medicine Dentistry and Pharmaceutical Sciences Okayama University Okayama Japan

**Keywords:** breast cancer, CD8^+^ T cells, immunotherapy, MAPK/ERK, tumor microenvironment

## Abstract

Activation of the RAS/RAF/ERK pathway is crucial for adaptive immunity. Here, we provide evidence that Sprouty‐related EVH1 domain containing 2 (SPRED2), an endogenous inhibitor of this pathway, negatively regulates CD8^+^ T cell‐mediated antitumor immunity in breast cancer. In EO771 and 4T1 mouse models, *Spred2^−/−^* mice exhibit reduced tumor growth, with lower endpoint lung metastatic burden in the 4T1 model, accompanied by increased T‐cell infiltration and activation. *Spred2^−/−^* T cells show enhanced cytokine expression, proliferation, survival, in vitro cytotoxicity, and memory‐like phenotypes, particularly among CD8^+^ T cells. Adoptive transfer of *Spred2^−/−^* CD8^+^ T cells into tumor‐bearing wild‐type hosts similarly reduces endpoint lung metastatic burden. MEK inhibition with U0126 attenuates increased interferon‐gamma and granzyme B expression in *Spred2^−/−^* CD8^+^ T cells. Transcriptomic analyses reveal an inverse correlation between *SPRED2* expression and antitumor CD8^+^ T cell states in both humans and mice. In tumor‐infiltrating T cells from patients with breast cancer, *SPRED2^low^* T cells show enriched effector and cytotoxicity programs and are associated with “immune‐hot” tumors, whereas survival associations vary with CD8^+^ T cell context. Collectively, these findings suggest that SPRED2 functions as a cell‐intrinsic negative regulator of CD8^+^ T‐cell activation and highlight its potential as a therapeutic target to enhance T cell–based cancer immunotherapy.

## Graphical abstract

Sprouty‑related EVH1 domain containing 2 (SPRED2) restrains Ras/Raf/ERK signaling in CD8^+^ T cells. Host SPRED2 deficiency enhances T‐cell survival, cytotoxicity, and memory‐like phenotypes, reducing primary tumor growth and endpoint lung metastatic burden in breast cancer models. In patients with triple‐negative breast cancer, low SPRED2 in tumor‐infiltrating T cells marks effector‐like, immune‐hot tumors, identifying SPRED2 as a candidate therapeutic target.

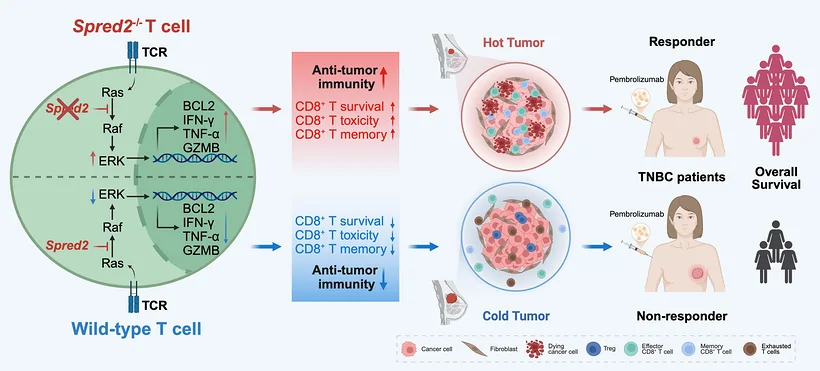

## 1 Introduction

T‐cell activation is initiated by the binding of antigenic peptide‐MHC complexes to T‐cell receptors (TCRs) on the cell surface. The proximal signaling is first generated by the activation of a set of protein tyrosine kinases, such as LCK, FYN, and ZAP‐70, followed by the activation of multiple distal signaling pathways, including the Ca^2+^‐calcineurin‐NFAT, PKCθ‐IKK‐NF‐κB, RASGRP1‐RAS‐ERK1/2, and TSC1/2‐mTOR pathways [1]. Concurrently, inhibitory signals are generated by co‐inhibitory receptors, known as immune checkpoints (ICPs), such as PD‐1 and CTLA4, to prevent excessive T‐cell activation and preserve immune homeostasis. However, this regulatory mechanism can also dampen protective immune responses following vaccination or during chronic infection and cancer [2, 3, 4].

Immunotherapy aiming at enhancing the patients’ own immune system has emerged as a powerful strategy for cancer treatment. Inhibition of immune checkpoints by monoclonal antibodies (mAbs) or adoptive transfer of chimeric antigen receptor (CAR) T cells has shown remarkable clinical efficacy in certain settings [5]. A growing number of intracellular molecules that block TCR signaling, known as intracellular ICPs (iICPs), have also been identified [4]. Thus, a deeper understanding of the mechanisms governing T‐cell activation and the identification of new molecular targets to potentiate T‐cell responses are necessary for the next generation of cancer immunotherapy.

The ERK signaling pathway, one of the distal signaling pathways downstream of TCR, controls the expression of a relatively small subset of proteins but selectively controls the expression of critical effector molecules and cytokines that enable T‐cell communication with other immune cells to mediate adaptive immune responses [6]. Sprouty‐related EVH1 domain containing 2 (SPRED2) is a member of the SPRED protein family and acts as a crucial endogenous inhibitor of the RAS/RAF/ERK signaling pathway. Its inhibitory function is primarily attributed to its binding to RAF, which leads to the suppression of the downstream ERK signaling pathway [7]. We and others have demonstrated that SPRED2 suppresses not only the proliferation, malignant behaviors, and survival of cancer cells [8, 9, 10], but also the development of inflammatory responses [11, 12]. Furthermore, SPRED2 protects mice from Concanavalin A (Con A)‐induced, T cell‐dependent liver injury by downregulating the production of the Th1 cytokine interferon‐gamma (IFN‐γ) and the chemokines CXCL9 and CXCL10 [13]. These findings strongly suggest that SPRED2 acts as a negative regulator of the TCR‐mediated T‐cell activation and that the deletion of SPRED2 in T cells may enhance antitumor immune responses.

Breast cancer (BC) is the leading cause of cancer‐related mortality among women worldwide, with most deaths attributed to drug resistance and metastasis [14]. Triple‐negative breast cancer (TNBC), characterized by the absence of estrogen receptor, progesterone receptor, and HER2/ERBB2 expression, is an aggressive subtype with poor prognosis [14]. In the absence of therapeutic targets, chemotherapy remains the standard treatment for TNBC, although some patients do not respond well to chemotherapy [15]. Interestingly, an abundance of tumor‐infiltrating lymphocytes (TILs) correlates with a better prognosis in stage I TNBC patients [16], suggesting the possibility of treating TNBC patients with immunotherapy; however, attempts to treat TNBC patients with immunotherapy have been unsuccessful [17].

In the present study, we investigated whether targeting SPRED2 augments antitumor immune responses against BC by transplanting two commonly used murine BC cell lines, EO771 (luminal B) and 4T1 (TN), into SPRED2‐deficient (*Spred2^−/−^*) mice. We demonstrate that SPRED2 restrains the effector function, survival, and differentiation of CD8^+^ T cells, in part by suppressing the ERK signaling pathway. Furthermore, transcriptomic analyses of publicly available datasets reveal that *SPRED2* expression is dynamically modulated in CD8^+^ T cells upon TCR activation and that tumor‐infiltrating T cells from immunotherapy responders exhibit lower *SPRED2* expression than those from non‐responders, although this difference does not reach statistical significance at the patient level. Thus, we propose that SPRED2 functions as a cell‐intrinsic negative regulator that fine‐tunes ERK activity in CD8^+^ T cells and thereby negatively regulates antitumor immune responses. SPRED2 may therefore represent a candidate molecular target for enhancing antitumor immunity against solid tumors, including BC.

## 2 Results

### 2.1 SPRED2 Deficiency in the Host Suppresses the Progression of EO771 and 4T1 BC Via Increased Antitumor Immune Responses

**FIGURE 1:**
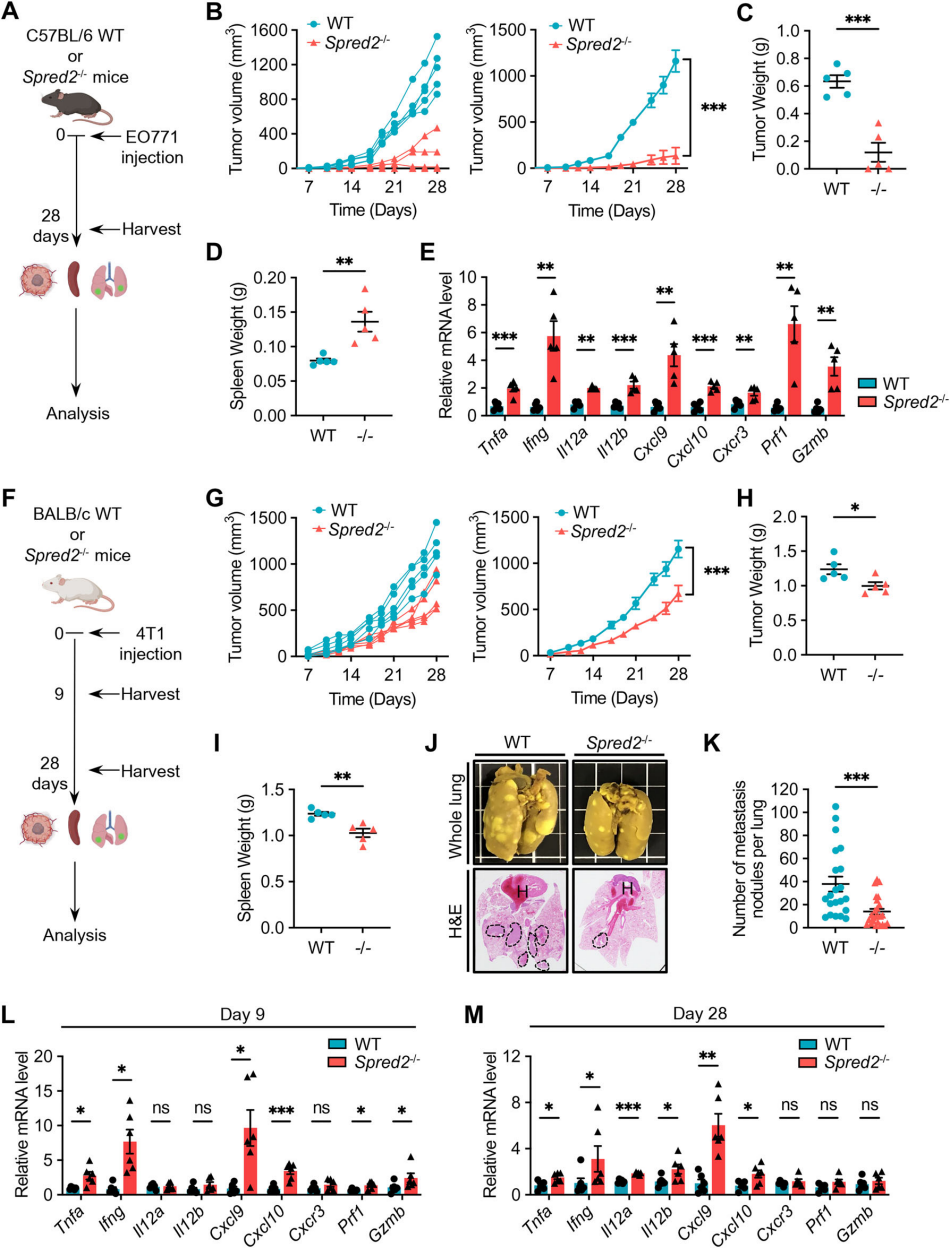
Host SPRED2 deficiency suppresses breast tumor growth and endpoint lung metastatic burden. (A–E) EO771 cells (1 × 10^5^ in 100 µL PBS) were injected into the mammary fat pad of C57BL/6J WT or *Spred2^−/−^* mice (*n* = 5 per group). (A) EO771 model design; (B) EO771 tumor growth: individual tumor trajectories (left) and means (right). (C–E) Day 28 analysis: (C) primary tumor weight; (D) spleen weight; (E) tumor immune‐gene expression by RT‐qPCR. (F–M) 4T1 cells (5 × 10^4^ in 100 µL PBS) were injected into the mammary fat pad of BALB/c WT or *Spred2^−/−^* mice (G–J; *n* = 5 per group). (F) 4T1 model design; (G) 4T1 tumor growth: individual tumor trajectories (left) and means (right). (H–K) Day 28 analysis: (H) primary tumor weight; (I) spleen weight; (J) lungs after fixation in Bouin's solution (upper panel) and H&E‐stained sections (lower panel); (K) macroscopic lung metastatic nodules (WT *n* = 21, *Spred2^−/−^ n* = 25; pooled from 3 experiments, each point = one mouse). (L, M) tumor‐immune‐gene expression on day 9 (L) and 28 (M) by RT‐qPCR (*n* = 6 per group). Data are presented as mean ± SEM. Statistics: (B, G) two‐way repeated‐measures ANOVA followed by Šídák's multiple‐comparisons test; (C, D, H, I) unpaired two‐tailed Student's *t*‐test; (K) two‐tailed Mann–Whitney U test; (E, L, M) unpaired two‐tailed Student's t‐test with Holm–Šídák's correction. ^*^ *p* < 0.05, ^**^ *p* < 0.01, ^***^ *p* < 0.001.

To test the hypothesis that host SPRED2 deficiency enhances antitumor immune responses against BC, we first employed the EO771 model (Figure 1A). EO771 is a luminal B BC cell line derived from a tumor that spontaneously arose in a C57BL/6J mouse and has been shown to respond to immunotherapy [18]. After implantation of EO771 cells into the mammary fat pads, tumors in *Spred2^−/−^* mice were smaller than those in wild‐type (WT) mice, with approximately 80% reduction in tumor weights on day 28 (Figure 1B,C). Spleen weights were higher in tumor‐bearing *Spred2^−/−^* mice compared to WT mice (Figure 1D), although the cellular basis of this difference was not determined. There was no lung metastasis in both WT or *Spred2^−/−^* mice. Tumor‐associated inflammation and elevated production of specific cytokines within the tumor microenvironment (TME) are hallmarks of antitumor immune responses [19]. Analysis by quantitative real‑time PCR (RT‐qPCR) revealed that mRNA levels of cytokines, including *Tnfa* and *Ifng*, *Il12a* and *Il12b*, IFN‐γ‐inducible chemokines, including *Cxcl9* and *Cxcl10*, and their receptor *Cxcr3* [20], and cytotoxic molecules, including perforin‐1 *(Prf1)* and granzyme B *(Gzmb)*, were all upregulated in tumors from *Spred2^−/−^* mice on day 28 (Figure 1E).

We next employed the 4T1 model, which is more resistant to immunotherapy, including immune checkpoint blockade, than the EO771 model [21]. Unlike EO771 cells, 4T1 cells spontaneously metastasize to the lung. Since 4T1 cells are derived from a tumor that arose in a BALB/c mouse, we backcrossed C57BL/6J *Spred2^−/−^* mice with BALB/c WT mice for 10 generations. We then orthotopically implanted 4T1 cells into the mammary fat pads of BALB/c WT and *Spred2^−/−^* mice and compared tumor growth and lung metastasis (Figure 1F). Tumor growth in *Spred2^−/−^* mice was slower than in WT mice (Figure 1G), with approximately a 20% reduction in tumor weights on day 28 (Figure 1H). In contrast to the EO771 model, spleen weights were reduced by approximately 20% in *Spred2^−/−^* mice compared to WT mice (Figure 1I). Splenomegaly is one of the characteristics of this cancer model due to increased myelopoiesis caused by growth factors secreted by 4T1 cells, likely explaining the smaller spleens in *Spred2^−/−^* mice. Importantly, the number of macroscopic lung metastatic nodules counted at the experimental endpoint (day 28) was reduced by approximately 70% in *Spred2^−/−^* mice compared with WT mice (Figure 1J,K). In addition, many metastatic nodules in WT mice were large (> 2 × 2 mm), whereas most nodules in *Spred2^−/−^* lungs were small (< 1 × 1 mm). By RT‐qPCR, mRNA levels of cytokines, including *Tnfa* and *Ifng*, and chemokines *Cxcl9* and *Cxcl10*, were all upregulated in 4T1 tumors from *Spred2^−/−^* mice on day 9 and day 28 (Figure 1L,M). Expression of the cytotoxic molecules *Prf1* and *Gzmb* was elevated on day 9 (Figure 1L), while the expression of *Il12a* and *Il12b* was upregulated on day 28 (Figure 1M). Collectively, these results support the view that host SPRED2 deficiency promotes the development of antitumor immune responses and suppresses EO771 and 4T1 tumor progression. Considering that metastasis is a major cause of cancer‐related death in BC patients, the reduced endpoint lung metastatic burden detected in *Spred2^−/−^* mice is clinically important. Accordingly, we performed further studies mainly focusing on the 4T1 model.

### 2.2 Host SPRED2 Deficiency Facilitates a CD8^+^ T‐Cell–Dependent Antitumor Response and Reduces Endpoint Lung Metastatic Burden

**FIGURE 2:**
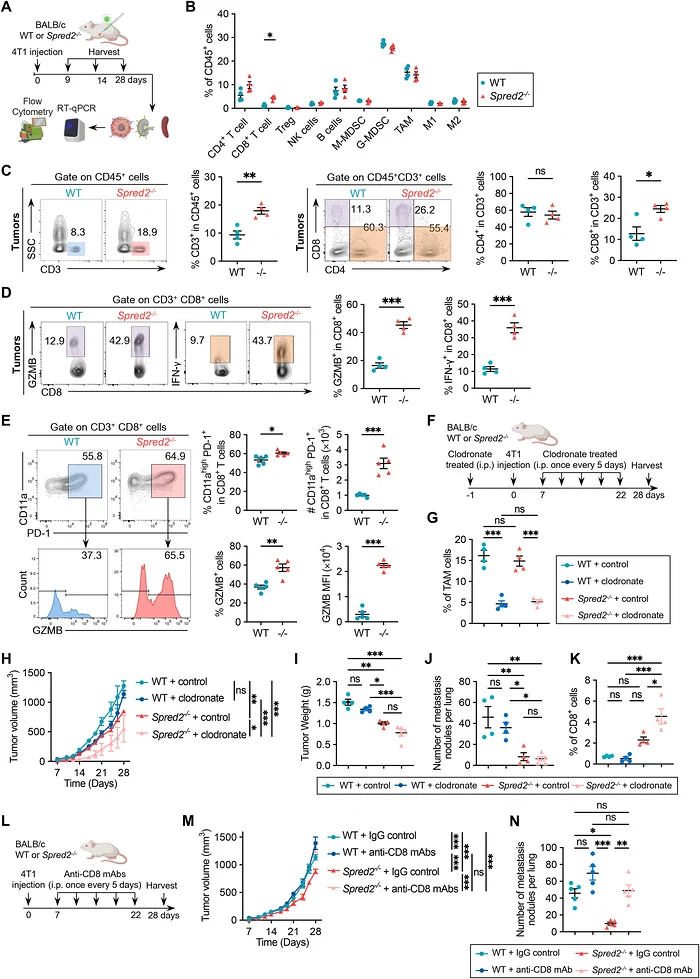
CD8^+^ T cell‐dominated remodeling of antitumor immunity in SPRED2‐deficient hosts. (A–E) 4T1 cells (5 × 10^4^ in 100 µL PBS) were injected into the mammary fat pad of WT or *Spred2^−/−^* mice (B–D, *n* = 4; E, *n* = 5 per group). (A) Experimental design; (B) Tumor immune cell composition: the frequency of each population as a percentage of CD45^+^ leukocytes; (C) Representative flow cytometry plots and quantification of tumor‐infiltrating T cells: CD3^+^ cells (% of CD45^+^), CD4^+^ and CD8^+^ T cells (% of CD3^+^); (D) Representative flow cytometry plots and the frequency of granzyme B (GZMB)^+^ and IFN‐γ^+^ cells (% of CD8^+^); (E) Antigen‐experienced (putatively tumor‐reactive) CD8^+^ T cells: CD11a vs. PD‐1 contour plots (gated CD3^+^CD8^+^): frequency and number of CD11a^high^ PD‐1^+^ cells, and GZMB^+^ frequency and mean fluorescence intensity (MFI) within this subset. (F–K) WT and *Spred2^−/−^* mice received clodronate liposomes or control liposomes from one day before tumor implantation and then every 5 days until harvest (*n* = 4 per group). (F) Experimental design for macrophage depletion; (G) Tumor‐associated macrophage frequency (% of CD45^+^ leukocytes) after clodronate treatment; (H) Primary tumor growth curves; (I) Endpoint primary tumor weight; (J) Lung metastatic nodules; (K) CD8^+^ T cell frequency after clodronate treatment. (L–N) 4T1 tumor‐bearing WT and *Spred2^−/−^* mice received anti‐CD8 mAb (Lyt‐2.2) or control IgG every 5 days until harvest (*n* = 5 per group). (L) Experimental design for CD8^+^ T‐cell depletion; (M) Primary tumor growth curves; (N) Lung metastatic nodules. Data are presented as mean ± SEM. Statistics: (B) multiple unpaired *t*‐tests with Šídák's correction; (C–E) unpaired two‐tailed *t*‐test; (H, M) repeated‐measures two‐way ANOVA followed by Šídák's multiple‐comparisons test; (G, I–K, N) two‐way ANOVA with Šídák's correction. ^*^ *p* < 0.05, ^**^ *p* < 0.01, ^***^ *p* < 0.001; ns, not significant.

To understand the mechanisms underlying the potent antitumor effects observed in *Spred2^−/−^* mice, we characterized leukocyte infiltration in the TME and in secondary lymphoid organs of 4T1 tumor‐bearing mice (Figure 2A). Among the CD45^+^ leukocyte populations examined, the frequencies of T cells selectively increased in *Spred2^−/−^* hosts, whereas the frequencies of other immune cell populations, including myeloid cells, natural killer (NK) cells, and B cells, did not differ significantly between genotypes (Figure 2B), consistent with the essential role of T cells in the rejection of tumors, such as 4T1 and EO771 [22, 23]. Within the CD3^+^ T‐cell compartment, this increase was attributable to CD8^+^ T cells, whereas the frequency of CD4^+^ T cells remained unchanged (Figure 2C). Furthermore, intratumoral CD8^+^ T cells from *Spred2^−/−^* mice displayed a markedly higher fraction of GZMB^+^ and IFN‐γ^+^ cells compared with WT controls (Figure 2D), consistent with enhanced cytotoxic effector function. This selective CD8^+^ T‐cell expansion was also observed in secondary lymphoid organs, including tumor‐draining lymph nodes (TDLNs) and spleen (Figure S1A–E), and was accompanied by elevated expression of effector‐ and memory‐associated genes, including *Ifng*, *Gzmb*, *Prf1*, *Cxcr3*, and *Tbx21*, in the spleen (Figure S1F,G). Because defined immunodominant MHC class I antigens are not available in the 4T1 model, we used CD11a^high^PD‐1^+^ expression as an antigen‐independent surrogate phenotype for antigen‐experienced (putatively tumor‐reactive) CD8^+^ T cells [24, 25]. Among tumor‐infiltrating CD8^+^ T cells, both the frequency and absolute number of CD11a^high^PD‐1^+^ cells were higher in *Spred2^−/−^* than in WT mice (Figure 2E). Moreover, a greater proportion of this subset expressed GZMB, with higher per‐cell GZMB content (Figure 2E), indicating enhanced accumulation and effector arming of antigen‐experienced (putatively tumor‐reactive) CD8^+^ T cells in *Spred2^−/−^* tumors. A similar increase was observed in the broader PD‐1^+^ CD8^+^ T‐cell population, which also showed elevated GZMB expression (Figure S2).

In contrast, the proportion of CD11b^+^ F4/80^+^ tumor‐associated macrophages (TAMs) and their polarization into NOS2^+^ (M1‐like) or CD206^+^ (M2‐like) subsets were comparable between WT and *Spred2^−/−^* mice (Figure 2B and Figure S3A,B), indicating that the loss of SPRED2 does not grossly alter TAM abundance or polarization at this stage. In the EO771 model, a histological analysis similarly revealed a selective increase in CD8^+^ T cells without appreciable changes in CD4^+^ T cells or M2‐like macrophages (Figure S3C,D). Furthermore, to more rigorously assess the contribution of myeloid SPRED2, we employed LysM‐Cre *Spred2* ^fl/fl^ mice and found that myeloid‐specific deletion of *Spred2* failed to recapitulate the tumor control observed in *Spred2^−/−^* mice (Figure S3E–I).

To further evaluate the contribution of SPRED2 deficiency in macrophages, we depleted TAMs using clodronate liposomes in 4T1 tumor‐bearing WT and *Spred2^−/−^* mice (Figure 2F). Clodronate efficiently reduced TAM frequency in both genotypes (from 16.2% ± 1.3% to 4.7% ± 0.7% in WT mice and from 14.9% ± 1.2% to 5.2% ± 0.5% in *Spred2^−/−^* mice; Figure 2G). In WT mice, TAM depletion did not significantly affect primary tumor growth (Figure 2H), tumor weight (Figure 2I), or the number of lung metastatic nodules (Figure 2J), nor did it alter intratumoral CD8^+^ T‐cell frequency (Figure 2K). In *Spred2^−/−^* hosts, by contrast, TAM depletion was accompanied by a further reduction in primary tumor volume (Figure 2H), a similar trend toward reduced tumor weight (Figure 2I), and a further increase in intratumoral CD8^+^ T cells (Figure 2K). Notably, the number of lung metastases was unaffected by TAM depletion in either genotype (Figure 2J). Together, these data indicate that macrophages exert an immunosuppressive role in the TME, but that macrophage‐intrinsic SPRED2 is not the principal driver of the reduced endpoint lung metastatic burden observed in *Spred2^−/−^* mice. However, because clodronate liposomes also deplete other phagocytic populations, including dendritic cells (DCs), the reduction in primary tumor growth in *Spred2^−/−^* hosts cannot be attributed to TAMs alone.

To determine whether CD8^+^ T cells are the key population required for the enhanced antitumor phenotype and the reduced endpoint lung metastatic burden in *Spred2^−/−^* mice, we depleted CD8^+^ T cells with an anti‐CD8 mAb in 4T1 tumor‐bearing WT and *Spred2^−/−^* mice (Figure 2L). In *Spred2^−/−^* hosts, CD8^+^ T‐cell depletion reversed both the suppression of primary tumor growth (Figure 2M) and the reduction in endpoint lung metastatic burden (Figure 2N), restoring these parameters to levels comparable to those observed in WT mice. Together, our results indicate that the antitumor phenotype and the reduced endpoint lung metastatic burden of *Spred2^−/−^* hosts are driven principally by CD8^+^ T cells rather than by non–T‐cell populations.

### 2.3 SPRED2 Deficiency Enhances T‐Cell Proliferation and Survival as Well as CD8^+^ T‐Cell Effector Function In Vitro

**FIGURE 3:**
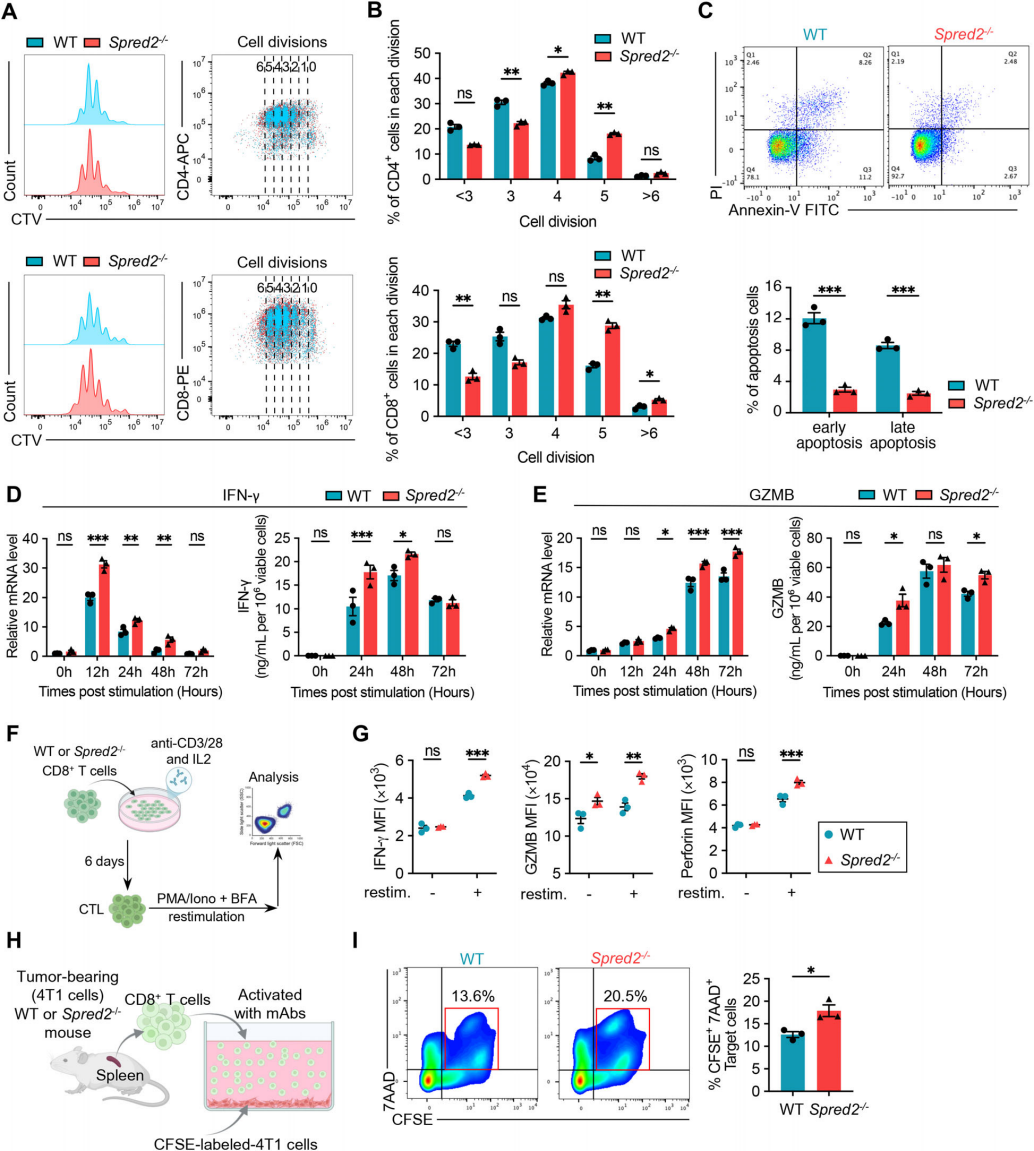
SPRED2 deficiency enhances activated T‐cell division and survival and CD8^+^ T‐cell effector function in vitro. (A–C) Splenic T cells from WT and *Spred2^−/−^* mice were stimulated with anti‐CD3e/CD28 mAbs for 72 h (*n* = 3 biological replicates per group). (A, B) Splenic T cells were labeled with CellTrace Violet (CTV) before the stimulation; (A) Representative CTV histograms and division‐gating plots are shown for CD4^+^/CD8^+^ T cells; (B) Per‐division proportions of CD4^+^/CD8^+^ T cells; (C) Representative flow cytometry plots (upper panel) and quantification of apoptotic cells (lower panel): Early (Annexin V^+^, PI^−^) and late (Annexin V^+^, PI^+^) apoptosis were analyzed at 72 h. (D, E) Naive CD8^+^ T cells from WT and *Spred2^−/−^* mice were stimulated with anti‐CD3e/CD28 mAbs; IFN‐γ (D) and GZMB (E) were measured at the indicated time points as mRNA (left, RT‐qPCR) and secreted protein (right, ELISA per 10^6^ viable cells) (*n* = 3 biological replicates). (F, G) Naive CD8^+^ T cells from WT or *Spred2^−/−^* mice were activated with anti‐CD3e/CD28 mAbs + IL‐2, differentiated to cytotoxic T lymphocytes (CTLs) over 6 days, restimulated with PMA/ionomycin + brefeldin A, and then intracellular flow‐cytometric analysis was performed. (F) Experimental design; (G) IFN‐γ, GZMB, and perforin MFI (mean fluorescence intensity) in CTLs ± restimulation (*n* = 3 biological replicates). (H, I) CD8^+^ T cells from 4T1‐bearing WT or *Spred2^−/−^* mice were stimulated with anti‐CD3e/CD28 mAbs for 48 h, and then co‐cultured with carboxyfluorescein succinimidyl ester (CFSE)‐labeled 4T1 target cells for 48 h (effector: target = 10: 1) (*n* = 3 biological replicates per group). (H) Experimental design; (I) Representative flow cytometry plots (left) and quantification of CFSE^+^7AAD^+^ target cells (right). Data are presented as mean ± SEM. Statistics: (B) repeated‐measures two‐way ANOVA with Šídák's multiple‐comparisons test; (C–E, G) two‐way ANOVA followed by Šídák's multiple‐comparisons test; (I) unpaired two‐tailed Student's *t*‐test. ^*^ *p* < 0.05, ^**^ *p* < 0.01, ^***^ *p* < 0.001; ns, not significant.

To determine whether SPRED2 deficiency in T cells underlies the robust antitumor effects against 4T1 cells, we isolated CD3^+^ T cells from the spleens of naive BALB/c WT or *Spred2^−/−^* mice and stimulated them with anti‐CD3e/CD28 mAbs. The number of *Spred2^−/−^* T cells was greater than that of WT T cells over a 96‐h culture period (Figure S4A). To track cell division, splenic T cells from WT and *Spred2^−/−^* mice were labeled with CellTrace Violet (CTV) and stimulated with anti‐CD3e/CD28 mAbs for 72 h. Quantification of cell proportions in each division showed that *Spred2^−/−^* CD4^+^ and CD8^+^ T cells were distributed toward higher division numbers than WT cells, indicating enhanced TCR‐driven proliferation (Figure 3A,B). *Spred2^−/−^* T cells also showed reduced early (Annexin V^+^, PI^−^) and late‐stage (Annexin V^+^, PI^+^) apoptosis at 72 h after TCR activation (Figure 3C). Consistent with our previous findings using cells on the C57BL/6J background [13], naive resting CD8^+^ T cells from both WT and *Spred2^−/−^* BALB/c mice released only low levels of IFN‐γ (Figure S4B). Upon TCR stimulation, however, CD8^+^ T cells from *Spred2^−/−^* mice expressed and released significantly higher levels of IFN‐γ (Figure 3D) and GZMB (Figure 3E). To assess effector molecule production on a per‐cell basis in cytotoxic T lymphocytes (CTLs), naive splenic CD8^+^ T cells from both genotypes were differentiated into CTLs and then restimulated with PMA/ionomycin for 6 h (Figure 3F). The MFIs of IFN‐γ, GZMB, and perforin were all higher in *Spred2^−/−^* than in WT CTLs. Without restimulation, these effector molecules were comparable between genotypes except for GZMB, which showed a modest increase in *Spred2^−/−^* CTLs (Figure 3G and Figure S4C,D). Because GZMB is pre‐stored in cytotoxic granules whereas IFN‐γ is produced de novo upon restimulation, this baseline difference in GZMB likely reflects a larger pre‐formed pool established during differentiation, consistent with the higher GZMB observed over the activation time course (Figure 3E). Together, these findings indicate that SPRED2 deficiency increases the per‐cell production capacity of CD8^+^ T‐cell effector molecules.

The increased production of effector molecules by *Spred2^−/−^* CD8^+^ T cells suggested enhanced cytotoxicity. To assess this, we conducted an in vitro T cell killing assay using 4T1 cells. CD8^+^ T cells were isolated from naive WT or *Spred2^−/−^* mice, activated in vitro with anti‐CD3e/CD28 mAbs, and then co‐cultured with carboxyfluorescein succinimidyl ester (CFSE)‐labeled 4T1 tumor cells for 48 h (Figure S4E). *Spred2^−/−^* CD8^+^ T cells showed greater in vitro killing of CFSE‐labeled 4T1 target cells than WT cells (Figure S4F). A similar advantage was observed when CD8^+^ T cells were isolated from the spleens of 4T1 tumor‐bearing mice (Figure 3H,I). These data indicated that CD8^+^ T cells secrete higher levels of effector molecules in the absence of SPRED2 and display enhanced in vitro killing of 4T1 target cells.

### 2.4 SPRED2 Deficiency Increases the Frequency of Memory‐Like CD8^+^ T Cells

**FIGURE 4:**
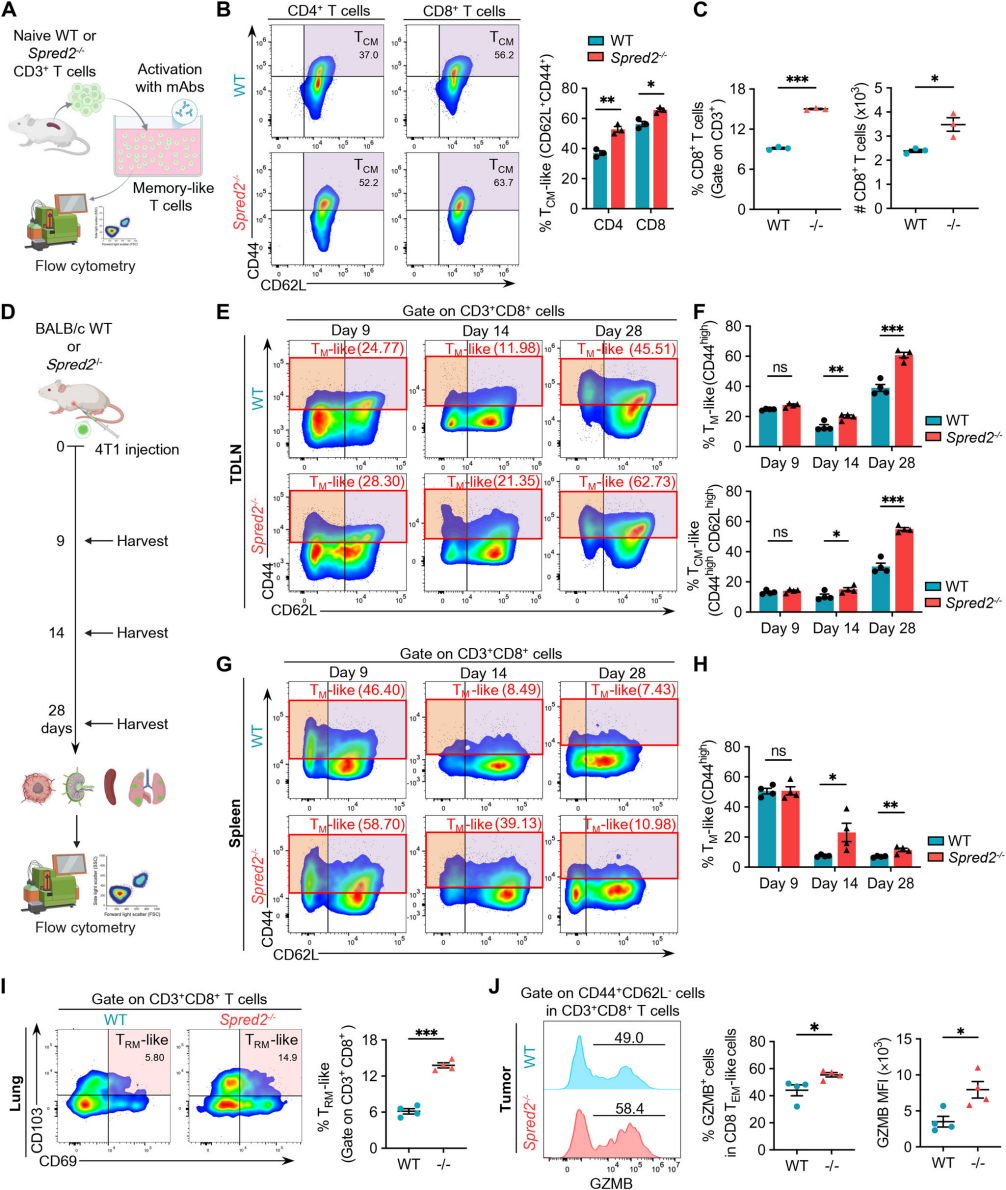
SPRED2 deficiency increases memory‐like CD8^+^ T cells in 4T1 tumor‐bearing mice. (A–C) Pan‐T cells from naive WT or *Spred2^−/−^* mice were stimulated with anti‐CD3e/CD28 mAbs for 2 weeks in vitro (*n* = 3 biological replicates per group). (A) Experimental design; (B) Representative flow cytometry plots (left) and frequency of memory phenotype (T_CM_‐like) of CD4^+^/CD8^+^ T cells by flow cytometry (right); (C) Frequency (% of CD3^+^, left) and absolute number of CD8^+^ T cells (right) after activation/memory differentiation. (D–J) 4T1 cells (5 × 10^4^ in 100 µL PBS) were injected into the mammary fat pad of WT or *Spred2^−/−^* mice, and primary tumors, tumor‐draining lymph nodes (TDLNs), spleens, and lungs were harvested (*n* = 4 mice per group at each time point). (D) Experimental design; (E–H) Representative flow cytometry plots and the frequency of T_M_‐like (CD44^high^), T_CM_‐like (CD44^high^CD62L^high^), and T_EM_‐like (CD44^high^CD62L^low^) within CD3^+^CD8^+^ cells in TDLNs (E, F) and spleens (G, H) by flow cytometry; (I) Representative flow cytometry plots and the frequency of T_RM_‐like (CD69^high^CD103^high^) within CD3^+^CD8^+^ cells in lungs (day 28) by flow cytometry; (J) Tumor‐infiltrating T_EM_‐like (CD44^high^CD62L^low^) within CD3^+^CD8^+^ cells (day 28): Left, representative GZMB histograms by flow cytometry; Right, GZMB^+^ frequency and GZMB MFI (mean fluorescence intensity) per 10^3^ cells. Data are presented as mean ± SEM. Statistics: (B, F, H) two‐way ANOVA followed by Šídák's multiple‐comparisons test; (C, I, J) unpaired two‐tailed Student's *t*‐test. ^*^ *p* < 0.05, ^**^ *p* < 0.01, ^***^ *p* < 0.001; ns, not significant. T_CM_‐like, central memory‐like T cells; T_M_‐like, memory‐like T cells; T_EM_‐like, effector memory‐like T cells; T_RM‐_like, tissue‐resident memory‐like T cells.

We next investigated the impact of SPRED2 deficiency on memory T‐cell differentiation. Naive WT and *Spred2^−/−^* T cells were cultured on plates coated with anti‐CD3e/CD28 mAbs for 2 weeks, and the memory phenotype of surviving T cells was analyzed by flow cytometry (Figure 4A). During the culture, T cells undergo proliferation, differentiation, and contraction. Compared to WT cells, surviving *Spred2^−/−^* T cells exhibited a significantly higher frequency of central memory‐like T cells (T_CM_‐like) characterized by the CD8^+^CD44^high^CD62L^high^ or CD4^+^CD44^high^CD62L^high^ phenotype (Figure 4B). The frequency and the number of CD8^+^ T cells were also significantly increased in the *Spred2^−/−^* group relative to the WT group (Figure 4C).

As shown above (Figure S1F,G), the expression of *Cxcr3* and *Tbx21*, genes associated with enhanced effector‐memory status and T_CM_‐like differentiation [26, 27], was elevated in whole‐spleen tissue of tumor‐bearing mice throughout tumor progression, including at advanced stages when T‐cell exhaustion occurs. Although these bulk‐tissue measurements do not resolve the cellular source of these transcripts, they prompted us to evaluate the frequency of memory‐like T cells (T_M_‐like) directly by flow cytometry in the lymphoid organs and lungs of 4T1 tumor‐bearing mice (Figure 4D). In the TDLNs of *Spred2^−/−^* mice, the frequency of CD8^+^ T_M_‐like cells (CD8^+^CD44^high^) was significantly higher than that of WT mice, particularly at advanced stages of tumor progression (Figure 4E,F). The frequency of CD8^+^ T_CM_‐like cells (CD44^high^CD62L^high^) was also significantly increased in the TDLNs of *Spred2^−/−^* mice (Figure 4F). Although the frequency of CD8^+^ T_M_‐like cells in the spleen decreased during tumor progression on day 14 and 28 in both WT and *Spred2^−/−^* mice, the decrease was smaller, and the frequency of CD8^+^ T_M_‐like cells was significantly higher in *Spred2^−/−^* mice compared to WT mice (Figure 4G,H). In the lung of tumor‐bearing mice at the advanced stage (on day 28), the frequency of tissue‐resident memory‐like T cells (T_RM_‐like, CD8^+^CD69^high^CD103^high^) was significantly increased in *Spred2^−/−^* mice (Figure 4I). To assess whether the expanded effector‐memory‐like cell compartment showed enhanced effector capacity, we examined the presence of GZMB in tumor‐infiltrating CD8^+^ effector‐memory‐like T cells (T_EM_‐like, CD44^high^CD62L^low^). Both the frequency of GZMB^+^ cells and the per‐cell GZMB level (MFI) were significantly higher in tumor‐infiltrating CD8^+^ T_EM_‐like cells of *Spred2^−/−^* mice than in WT mice (Figure 4J). Together with the enhanced proliferation, survival, and effector function of *Spred2^−/−^* T cells described above, these results indicate that SPRED2 deficiency affects both the expansion and the differentiation of CD8^+^ T cells during tumor progression. Collectively, these findings indicate that SPRED2 deficiency is associated with higher frequencies of memory‐like CD8^+^ T cells in lymphoid organs and lung, and with greater GZMB content within tumor‐infiltrating T_EM_‐like cells, consistent with the enhanced antitumor response in *Spred2^−/−^* mice.

### 2.5 Adoptive Transfer of *Spred2^−/−^* CD8^+^ T Cells Suppresses Primary Tumor Growth and Endpoint Lung Metastatic Burden in the 4T1 Model

**FIGURE 5:**
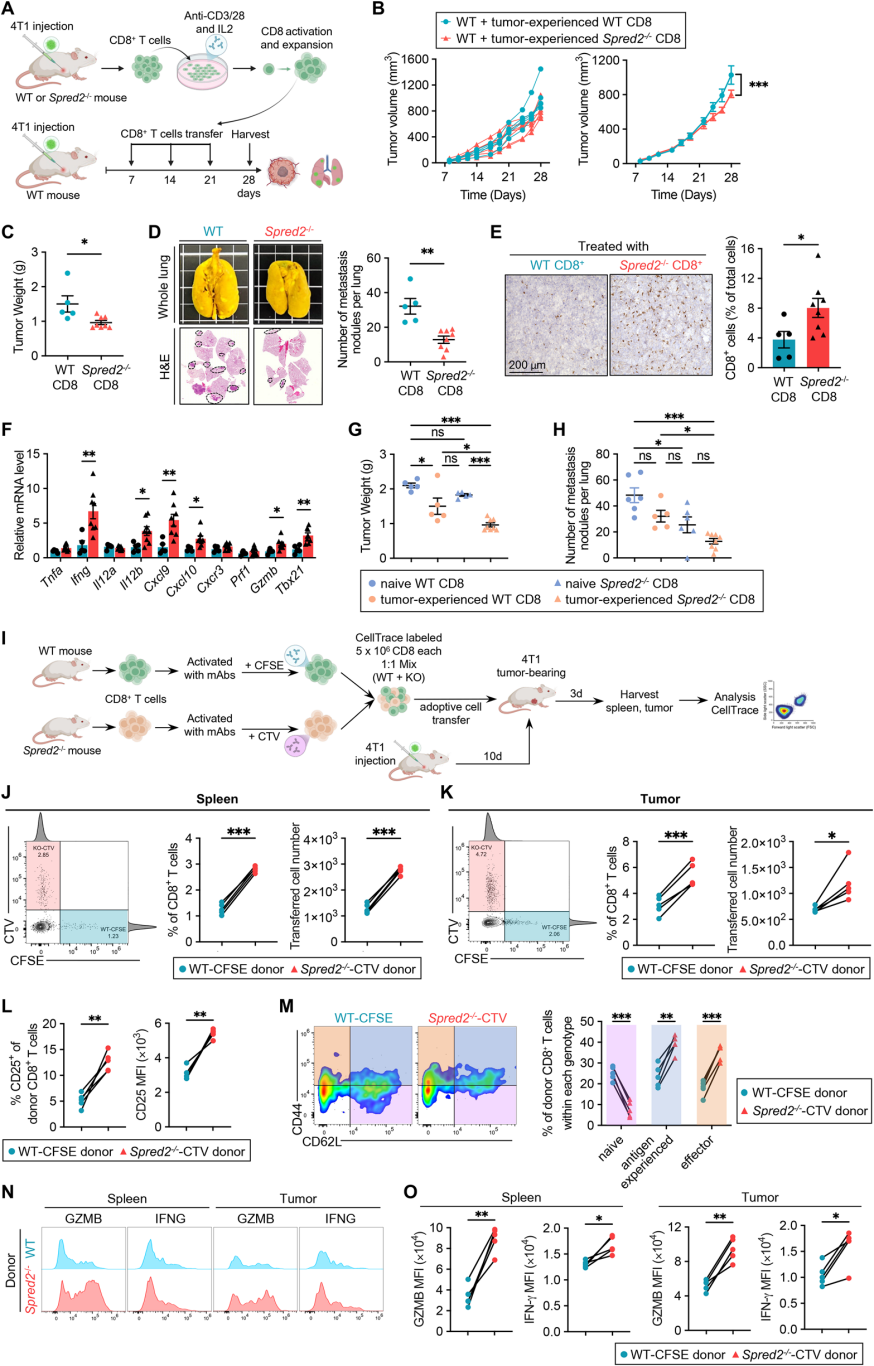
*Spred2^−/−^* CD8^+^ T cells exert enhanced antitumor activity after adoptive transfer. (A–H) 4T1 cells (5 × 10^4^ in 100 µL PBS) were injected into the mammary fat pad of WT recipients; donor splenic CD8^+^ T cells from 4T1‐bearing WT or *Spred2^−/−^* mice were expanded with anti‐CD3e/CD28 mAbs + IL‐2 for 3 days, transferred on days 7, 14, and 21 (5 × 10^6^ cells in 100 µL PBS per infusion); the primary tumors and lungs were harvested on day 28 (WT CD8^+^ transfer *n* = 5 mice, *Spred2^−/−^* CD8^+^ transfer *n* = 8 mice). (A) Experimental design; (B) Tumor growth; individual tumor trajectories (left) and means (right); (C) Tumor weight; (D) Left, lungs fixed in Bouin's solution (upper panel) and stained with hematoxylin and eosin (lower panel); Right, number of macroscopic metastatic nodules; (E) Tumor‐infiltrating CD8^+^ T cells by immunohistochemistry: representative images (left) and quantification by QuPath as percentage of positive cells (right); (F) Tumor immune‐gene expression by RT‐qPCR; (G, H) Primary tumor weight (G) and lung metastatic nodule number (H) in WT recipients given CD8^+^ T cells from naive or tumor‐bearing WT or *Spred2^−/−^* donors. (I–O) CD8^+^ T cells from WT and *Spred2^−/−^* mice were activated with anti‐CD3e/CD28 mAbs for 3 days, labeled with CFSE (WT) or CTV (CellTrace Violet; *Spred2^−/−^*), mixed at 1:1 (5 × 10^6^ cells of each), and transferred into WT mice bearing 10‐day 4T1 tumors; spleens and tumors were analyzed 3 days later (*n* = 5 biological replicates). (I) Experimental design for the competitive co‐transfer assay; (J, K) Recovery in spleen (J) and tumor (K): representative CTV vs. CFSE plots, the percentage of CD8^+^ T cells, and absolute transferred‐cell number; (L) The percentage of CD25^+^ and CD25 MFI in donor CD8^+^ T cells; (M) Donor differentiation by CD44/CD62L: representative plots and the percentage of naive (CD44^−^CD62L^+^), antigen‐experienced (CD44^+^CD62L^+^), and effector (CD44^+^CD62L^−^) cells; (N, O) GZMB and IFN‐γ in donor CD8^+^ T cells in spleen and tumor (ex vivo without restimulation): representative histograms (N) and MFI (O). Data are presented as mean ± SEM. Statistics: (B, G, H, M) two‐way ANOVA followed by Šídák's multiple‐comparisons test; (C, E) unpaired two‐tailed Student's *t*‐test; (D) two‐tailed Mann–Whitney U test; (F) multiple unpaired *t*‐tests with Šídák's correction; (J–L, O) paired two‐tailed Student's *t*‐test. ^*^ *p* < 0.05, ^**^ *p* < 0.01, ^***^ *p* < 0.001; ns, not significant.

Next, we isolated CD8^+^ T cells from tumor‐bearing WT and *Spred2^−/−^* mice, expanded them in vitro in the presence of anti‐CD3e/CD28 mAbs, and adoptively transferred them into tumor‐bearing WT mice on days 7, 14, and 21. Tumor growth and lung metastatic burden were evaluated on day 28 (Figure 5A). Under these conditions, adoptive transfer of *Spred2^−/−^* CD8^+^ T cells significantly suppressed tumor growth after the third transfer, resulting in smaller tumor volumes (Figure 5B) and tumor weights (Figure 5C) on day 28 compared with WT CD8^+^ T cells. The number of macroscopic lung metastatic nodules at the experimental endpoint (day 28) was reduced by approximately 60% (Figure 5D). Immunohistochemistry (IHC) showed increased intratumoral infiltration of CD8^+^ T cells (Figure 5E), and the expression of T cell response‐related genes, including *Ifng, Il12b, Cxcl9* and *Cxcl10, Gzmb* and *Tbx21*, was higher in mice receiving *Spred2^−/−^* CD8^+^ T cells than in those receiving WT CD8^+^ T cells (Figure 5F). When CD8^+^ T cells isolated from naive WT and *Spred2^−/−^* mice were expanded in vitro with anti‐CD3e/CD28 mAbs and then adoptively transferred, *Spred2^−/−^* CD8^+^ T cells also exerted stronger antitumor effects than WT CD8^+^ T cells (Figure S5A–F). Importantly, the antitumor effects of *Spred2^−/−^* CD8^+^ T cells were greater when the donor cells were derived from tumor‐bearing rather than naive mice (Figure 5G,H).

To assess whether the enhanced antitumor activity of *Spred2^−/−^* CD8^+^ T cells reflects a cell‐intrinsic property, we performed a competitive co‐transfer experiment. CD8^+^ T cells from WT and *Spred2^−/−^* mice were labeled with CFSE (WT) and CTV (*Spred2^−/−^*), respectively, mixed at a 1:1 ratio, co‐transferred into 4T1 tumor‐bearing WT recipients, and recovered 3 days later for analysis (Figure 5I). In both spleens and tumors, *Spred2^−/−^* CD8^+^ T cells were recovered at significantly higher frequencies and absolute numbers than co‐transferred WT cells (Figure 5J,K), and a label‐swap control yielded consistent results. *Spred2^−/−^* CD8^+^ T cells also expressed higher levels of the activation marker CD25 (Figure 5L) and were preferentially skewed toward antigen‐experienced (CD44^+^CD62L^+^) and effector (CD44^+^CD62L^−^) phenotypes, with a corresponding reduction in the naive (CD44^−^CD62L^+^) phenotype (Figure 5M). Consistently, *Spred2^−/−^* CD8^+^ T cells expressed higher levels of GZMB and IFN‐γ in both spleen and tumor directly ex vivo without restimulation (Figure 5N,O). Together, these data indicate that *Spred2^−/−^* CD8^+^ T cells possess a cell‐intrinsic advantage over WT CD8^+^ T cells in accumulation, activation, effector‐phenotype acquisition, and effector‐molecule expression, suggesting that adoptive transfer of SPRED2‐deficient T cells may represent a new strategy for cancer treatment.

### 2.6 SPRED2 Restrains ERK Signaling and Effector‐Molecule Production in Mouse CD8^+^ T Cells

**FIGURE 6:**
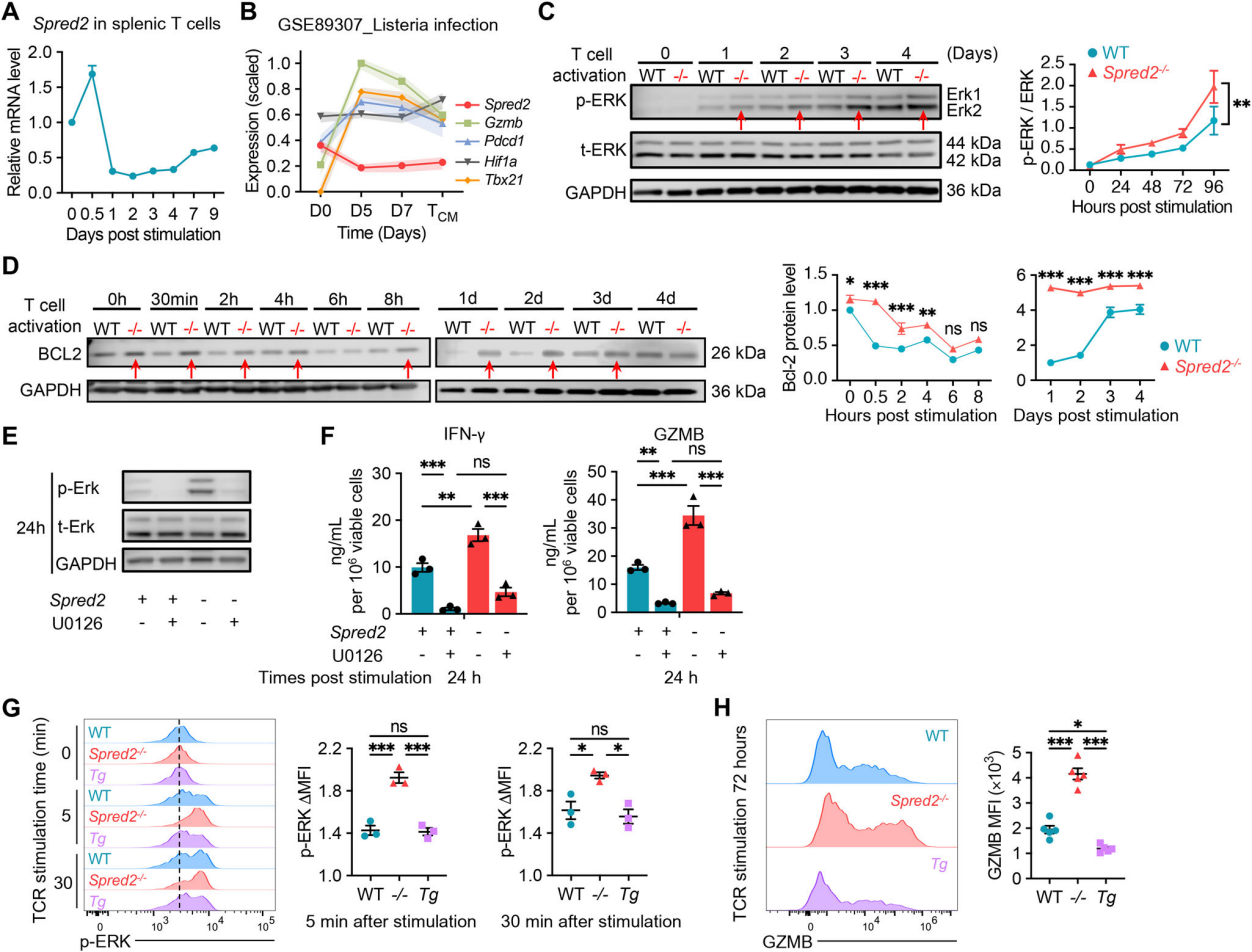
Dynamics of *Spred2* expression and SPRED2‐dependent ERK activation and effector‐molecule output in mouse CD8^+^ T cells. (A) Naive WT splenic pan‐T cells were stimulated with anti‐CD3e/CD28 mAbs over 9 days; *Spred2* expression was measured by RT‐qPCR across time points (*n* = 3 biological replicates per group). (B) Expression of *Spred2, Gzmb*, *Pdcd1*, *Hif1a*, and *Tbx21* in a published Listeria RNA‐seq dataset (GSE89307; *n* = 3 mice; mean ± 95% confidence intervals; expression scaled 0 to 1). (C, D) Naive splenic pan‐T cells from WT and *Spred2^−/−^* mice were activated with anti‐CD3e/CD28 mAbs for the indicated times. Left, representative phosphorylated ERK (p‐ERK), total ERK (t‐ERK) (C), and BCL2 (D) images by Western blot. Right, semi‐quantified data after normalization with GAPDH (*n* = 3 biological replicates per group). (E, F) Naive CD8^+^ T cells from WT and *Spred2^−/−^* mice were pre‐incubated with 10 µM U0126 or DMSO (Ctrl) for 30 min, then stimulated with anti‐CD3e/CD28 mAbs for 24 h. (E) Representative p‐ERK/t‐ERK expression by Western blotting. (F) IFN‐γ and GZMB concentrations were measured by ELISA (per 10^6^ viable cells) (*n* = 3 biological replicates). (G, H) WT, *Spred2^−/−^*, and *Spred2Tg* CD8^+^ T cells were stimulated with anti‐CD3e/CD28 cross‐linked with anti‐hamster IgG. (G) Left, representative p‐ERK histograms at 0/5/30 min after stimulation. Right, p‐ERK MFI at 5 and 30 min as fold over each genotype's 0‐min baseline (*n* = 3 biological replicates per genotype). (H) Intracellular GZMB after 72 h: representative histograms (left) and GZMB MFI (right) (*n* = 5 biological replicates per genotype). Data are presented as mean ± SEM. Statistics: (A) one‐way ANOVA followed by Dunnett's multiple‐comparisons test; (C, D) two‐way ANOVA followed by Šídák's multiple‐comparisons test; (F–H) one‐way ANOVA followed by Tukey's multiple‐comparisons test. ^*^ *p* < 0.05, ^**^ *p* < 0.01, ^***^ *p* < 0.001; ns, not significant.

SPRED2 is expressed in T cells and inhibits the RAS/RAF/ERK pathway; we therefore asked whether *SPRED2* expression itself is dynamically regulated during T‐cell activation. We activated mouse splenic T cells with anti‐CD3e/CD28 mAbs and analyzed the kinetics of *Spred2* mRNA. *Spred2* mRNA levels showed a transient increase during the early phase after TCR stimulation, followed by a marked decrease below the initial level from day 1 to day 4, and then a modest rebound on days 7 and 9 without exceeding the initial level (Figure 6A). Consistent with this, in a published dataset of mouse CD8^+^ T cells responding to acute Listeria monocytogenes infection (GSE89307) [28], *Spred2* mRNA level decreased by day 5 and remained low from the effector phase through to the central‐memory (T_CM_) stage, inversely tracking the effector‐ and activation‐associated genes *Gzmb*, *Tbx21*, and *Pdcd1*, whereas the *Hif1a* level remained unchanged—suggesting that these transcriptional changes are not accompanied by transcriptional induction of HIF1α (Figure 6B) [29]. Together, these observations suggest that endogenous *Spred2* level is dynamically regulated during CD8^+^ T‐cell activation and reduced during the effector‐expansion phase, coinciding with the acquisition of an activated effector phenotype.

To further examine ERK signaling and associated survival and effector changes, we analyzed splenic WT and *Spred2^−/−^* T cells. The level of p‐ERK increased from day 1 to day 4 after TCR stimulation in both genotypes, with a further increase in *Spred2^−/−^* T cells (Figure 6C). The level of the anti‐apoptotic protein BCL2, a regulator of T cell survival, was higher in *Spred2^−/−^* T cells than in WT T cells during activation, particularly at early (0–4 h) and later (after 24 h) time points (Figure 6D). This elevated BCL2 expression is consistent with the reduced apoptosis of *Spred2^−/−^* T cells during the proliferative phase (Figure 3A–C), although its functional contribution to this phenotype was not directly tested. Pretreatment with the MEK inhibitor U0126 abolished ERK activation in both WT and *Spred2^−/−^* T cells (Figure 6E) and reduced IFN‐γ and GZMB protein levels in the supernatants of both genotypes, such that the difference between genotypes was no longer significant (Figure 6F), indicating that the enhanced effector‐molecule production of *Spred2^−/−^* CD8^+^ T cells depends on MEK–ERK activity under these conditions. Notably, WT and *Spred2^−/−^* T cells showed no significant difference in apoptosis rates in the presence of U0126, with only a slight increase compared with vehicle controls (Figure S6). Because cytokine levels were normalized to viable cell numbers, the reduction in IFN‐γ and GZMB production following MEK inhibition is unlikely to reflect differences in cell death between genotypes and instead reflects a requirement for ERK signaling in effector cytokine production.

We next asked whether enforced SPRED2 expression could restrain TCR‐induced ERK signaling and effector‐molecule production. Previous work using SPRED2‐overexpressing transgenic (*Spred2Tg*) mice showed that SPRED2 overexpression reduces ERK activation and IFN‐γ production in Con A‐driven splenocyte activation [13]. In the present CD8^+^ T‐cell system, in which p‐ERK was quantified as the fold induction over each genotype's own 0‐min baseline, TCR‐induced ERK phosphorylation was increased in *Spred2^−/−^* CD8^+^ T cells, whereas no significant difference was observed between WT and *Spred2Tg* CD8^+^ T cells at either 5 or 30 min after stimulation (Figure 6G). As a significant level of endogenous SPRED2 is likely present in T cells in the early phase after TCR stimulation, enforced SPRED2 expression may have only a limited additional effect on the immediate ERK response. Although early ERK phosphorylation did not differ significantly between WT and *Spred2Tg* cells, GZMB accumulation after 72 h of stimulation was increased in *Spred2^−/−^* CD8^+^ T cells and decreased in *Spred2Tg* CD8^+^ T cells compared with WT cells (Figure 6H). Thus, enforced SPRED2 expression reduced downstream effector output despite the absence of a detectable reduction in early ERK phosphorylation in this assay. These results further support a model in which SPRED2 functions as a negative regulator of ERK‐dependent CD8^+^ T‐cell effector‐molecule production.

### 2.7 Low *SPRED2* Expression Marks Activated Effector States in Human CD8^+^ T Cells and TNBC‐Infiltrating T Cells

**FIGURE 7:**
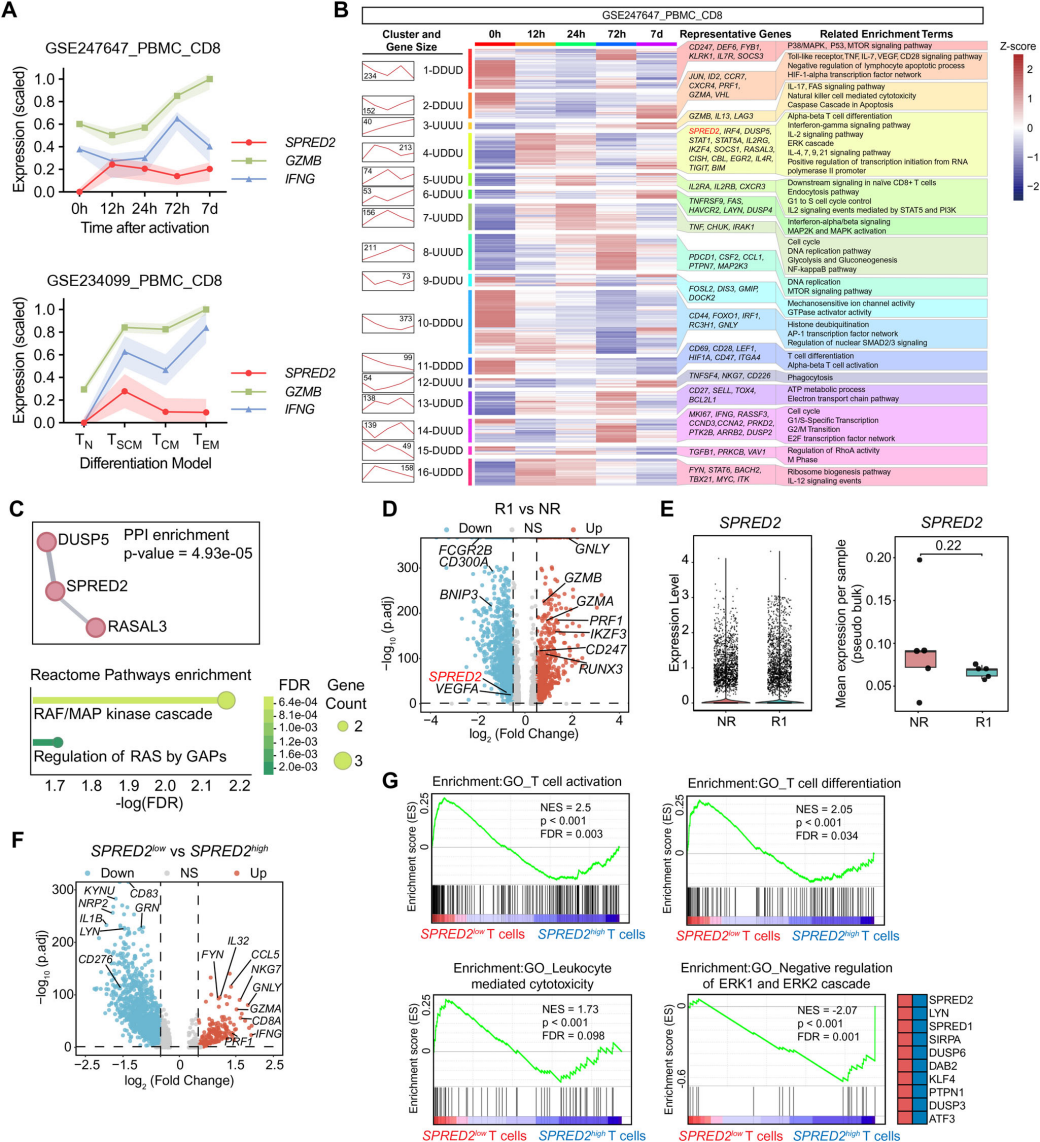
Low *SPRED2* marks activated effector states in human CD8^+^ T cells and TNBC‐infiltrating T cells. (A) Expression of *SPRED2*, *GZMB* and *IFNG* in vitro‐activated human CD8^+^ T cells (GSE247647, upper panel) and CD8^+^ T cells across in vitro differentiation stages (GSE234099, lower panel) (*n* = 3 per group; mean ± 95% confidence intervals; scaled 0–1). (B) EBSeq‐HMM time‐series transcriptional changes in human CD8^+^ T cells during TCR activation (GSE247647): line plots and heatmap for 16 gene clusters, with representative genes and related Gene Ontology (GO)/Kyoto Encyclopedia of Genes and Genomes (KEGG) terms listed for each cluster. (C) Cluster 4 protein–protein interaction (PPI) (STRING, confidence ≥ 0.4; MCL inflation = 3) showing the SPRED2‐containing cluster (edge width ∝ combined score) (upper panel) and Reactome enrichment (hypergeometric test, BH correction) (lower panel). (D) Volcano plot of T‐cell differentially expressed genes (DEGs) between responders and non‐responders (R1 vs. NR) in scRNA‐seq data of CD45‐positive cells from 10 baseline TNBC biopsies collected pre‐pembrolizumab (GSE246613; responders, R1, *n* = 5; non‐responders, NR, *n* = 5; up in R1 red, down blue; FDR < 0.05 with fold‐change threshold). (E) *SPRED2* expression by response group in the same dataset. Left, per‐cell violin plot with overlaid cells (each point = one cell). Right, per‐patient pseudobulk values (each point = one patient; NR, *n* = 5; R1, *n* = 5). Boxes show the median and interquartile range (Q1–Q3), and all individual values are plotted. Groups were compared by a two‐sided Wilcoxon rank‐sum test. (F) Volcano plot of DEGs between *SPRED2^low^* and *SPRED2^high^* T cells (up in *SPRED2^low^* red, down blue). (G) GSEA enrichment profiles for representative pathways in *SPRED2^low^* vs. *SPRED2^high^* T cells, with the leading‐edge genes shown; normalized enrichment score (NES), nominal P, and FDR q are shown for each.

To determine whether the inverse relationship between SPRED2 and CD8^+^ T‐cell activation observed in mice extends to human T cells, we analyzed publicly available human transcriptomic datasets. Naive CD8^+^ T cells isolated from peripheral blood mononuclear cells (PBMCs) were stimulated with anti‐CD3e/CD28 mAbs alone (GSE247647) or in combination with IL‐2 (GSE234099). In the first dataset, *SPRED2* expression was dynamically regulated during human CD8^+^ T‐cell activation: it was transiently upregulated early after stimulation and subsequently declined as the expression of effector‐associated genes, such as *IFNG* and *GZMB*, increased (Figure 7A, upper panel). Analysis of the second dataset, which examined PBMC‐derived CD8^+^ T cells across differentiation states, revealed a similar pattern: *SPRED2* mRNA levels were higher at early/stem‐like stages and declined as *IFNG* and *GZMB* expression rose (Figure 7A, lower panel). Thus, low *SPRED2* expression is a conserved feature of activated effector CD8^+^ T cells in both mice and humans.

To identify genes co‐expressed with SPRED2 during activation in human CD8^+^ T cells, we modeled the time‐series data from the first dataset (five activation time points) using EBSeq‐HMM and decomposed the dynamics into directional gene clusters (Figure 7B). This analysis placed *SPRED2* within a negative‐feedback module (cluster 4) containing other signal attenuators (*DUSP* and *EGR* family members, *CISH*, *SOCS1*, *RASAL3*). This cluster was enriched for the ERK cascade, IFN‐γ signaling, and T‐cell differentiation‐related terms, consistent with a feedback‐regulatory module. A compact protein–protein interaction (PPI) subnetwork further linked SPRED2 with the ERK phosphatase DUSP5 and the RAS‐GAP‐RASAL3, with enrichment for the RAF–MAP kinase cascade and regulation of RAS by GAPs (Figure 7C and Figure S7A–D). Although these human data analyses are correlative and do not constitute functional validation of SPRED2 loss of function, they are consistent with our findings in mice and suggest that SPRED2 acts within an ERK “brake” module during human CD8^+^ T‐cell activation.

We next asked whether *SPRED2* expression in tumor‐infiltrating T cells is associated with clinical responses to immune checkpoint blockade in patients with TNBC. We analyzed a single‐cell RNA‐sequencing dataset (GSE246613) of 10 baseline biopsies collected before the first cycle of pembrolizumab (responders, R1, *n* = 5; non‐responders, NR, *n* = 5) [30]. Consistent with previously described immune spatial phenotypes in TNBC [31, 32, 33], R1 biopsies showed high immune infiltration and the strongest T‐cell responses, characteristic of inflamed phenotypes, whereas NR biopsies aligned with immune‐ignored phenotypes (Table S1). Across 58,231 CD45^+^ cells, five cell clusters were identified—B, plasma, T, myeloid, and mast cells (Figure S8A). Within the T‐cell compartment (Figure S8B), *SPRED2* expression levels were lower in R1 than in NR T cells (Figure 7D,E, left panel); at the patient level, however, this difference did not reach statistical significance (*p* = 0.22, two‐sided Wilcoxon rank‐sum test; Figure 7E, right panel). These data show a directional difference in this small cohort and are hypothesis‐generating, warranting further investigation of whether low *SPRED2* expression in tumor‐infiltrating T cells is associated with favorable immunotherapy responses in larger cohorts.

To characterize the transcriptional programs associated with SPRED2 expression in tumor‐infiltrating T cells, T cells were pooled across all patients and stratified by relative *SPRED2* expression into *SPRED2^high^* and *SPRED2^low^* subpopulations. Effector‐associated genes (*CD8A*, *GNLY*, *GZMA*, *PRF1*, *IFNG*) were upregulated in *SPRED2^low^* T cells, whereas immunosuppression‐associated genes (*CD276*, *GRN*, *LYN*) were elevated in *SPRED2^high^* T cells (Figure 7F). Over‐representation analysis (Kyoto Encyclopedia of Genes and Genomes, KEGG; Gene Ontology biological process, GO_BP) showed enrichment of TCR signaling, T‐cell activation, differentiation, and cytotoxicity in *SPRED2^low^* T cells (Figure S8C,D). Gene set enrichment analysis (GSEA) confirmed higher enrichment for T‐cell activation, tumor cell killing, and negative regulation of apoptotic process in *SPRED2^low^* T cells, whereas *SPRED2^high^* T cells showed enrichment for negative regulation of ERK signaling (Figure 7G and Figure S8E). Bulk cross‐cohort co‐expression analysis independently corroborated the enrichment of immune‐effector programs among genes negatively correlated with *SPRED2* (Figure S9A–D). Together, these human data support the notion that low *SPRED2* expression marks activated effector states in tumor‐infiltrating T cells and identify *SPRED2^high^* T cells as a functionally distinct subpopulation with reduced effector programs.

### 2.8 *SPRED2* Expression is Inversely Associated With T‐Cell Effector Programs and Shows CD8‐Context‐Dependent Associations With Survival

**FIGURE 8:**
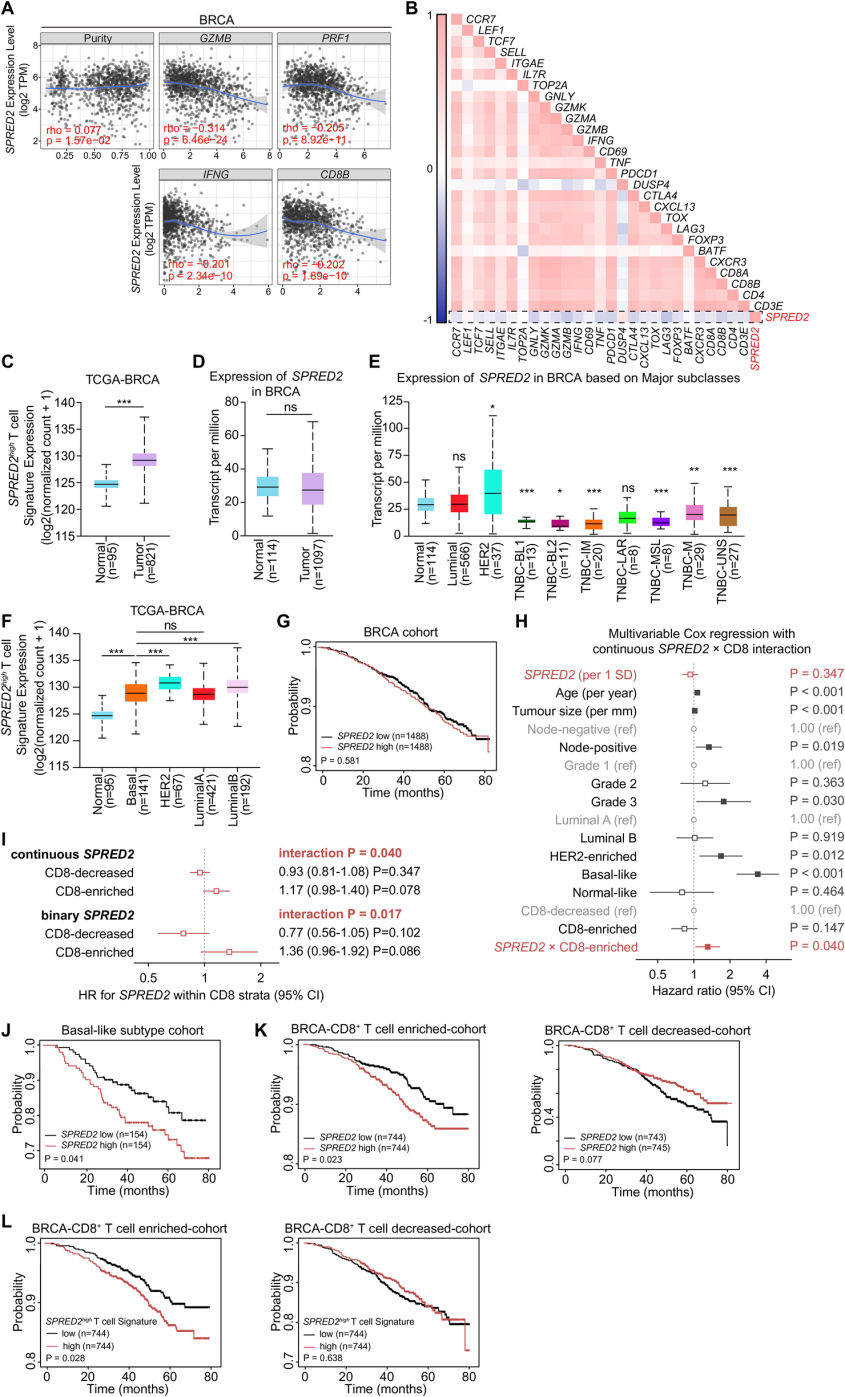
Low *SPRED2* and reduced *SPRED2^high^* T‐cell signature are associated with “immune‐hot” breast cancer and CD8‐context‐dependent survival. (A) Purity‐adjusted partial Spearman correlations (TIMER 3.0, TCGA‐BRCA) between SPRED2 and T cell/cytotoxic markers (purity as covariate). (B) Cross‐database validation (TNMplot) confirming negative SPRED2–effector‐gene correlations. (C) *SPRED2^high^* T‐cell signature in tumor vs. normal tissues (TCGA‐BRCA): box plots (median, IQR). (D) SPRED2 mRNA in BRCA vs. normal samples. (E) SPRED2 mRNA in normal breast tissue, luminal and HER2 breast cancers, and seven TNBC molecular subtypes. (F) *SPRED2^high^* T‐cell signature across PAM50 subtypes. (G) Overall survival (OS) in the whole BRCA cohort (SCAN‐B, GSE96058) stratified by *SPRED2* expression at the cohort median. (H) Multivariable Cox model for OS including continuous *SPRED2* expression (per 1 SD), CD8 stratum, their interaction, and adjustment for age, tumor size, nodal status, histological grade, and PAM50 subtype. Squares indicate hazard ratios (HRs) and horizontal lines indicate 95% confidence intervals (CIs). (I) CD8‐stratum‐specific *SPRED2* effects derived from the interaction models in (H) and Figure S9F. (J–L) OS stratified by *SPRED2* expression in Basal‐like tumors (J), by *SPRED2* expression in CD8^+^ T cell‐enriched and ‐decreased tumors (K), and by the *SPRED2^high^* T‐cell signature in CD8^+^ T cell‐enriched and ‐decreased tumors (L). Case number (n) is shown in the panels. Statistics: (A) partial Spearman correlation analysis adjusted for tumor purity; (B) Spearman correlation analysis; (C, D) two‐sided Wilcoxon rank‐sum test with FDR correction; (E, F) Kruskal–Wallis test with Dunn's post‐hoc test and FDR correction; (G–L) Kaplan–Meier groups were defined by the within‐cohort median, with ties assigned to the high group; *p* values are from log‐rank tests; HRs, CIs, Benjamini–Hochberg‐adjusted *p* values, and patient/event numbers for all six Kaplan–Meier comparisons are provided in Tables S4 and S5; interaction *p* values are from Wald tests. ^*^ *p* < 0.05, ^**^ *p* < 0.01, ^***^ *p* < 0.001.

To extend our observations from single‐cell data to bulk tumor cohorts, we asked whether *SPRED2* expression is inversely associated with CD8^+^/effector programs at the population level. We first performed tumor purity‐adjusted partial Spearman correlations using TIMER 3.0 in the TCGA‐BRCA cohort. *SPRED2* expression was inversely correlated with canonical T cell or cytotoxic marker genes (*GZMB*, *PRF1*, *IFNG*, *CD8B*, and *CD3D*) (Figure 8A and Figure S9E). *CD8A* showed the same direction with a smaller effect size, whereas *CD4* was not significantly associated (Figure S9E). Notably, *SPRED2* itself showed only weak correlation with tumor purity (ρ = 0.077), indicating these inverse associations are unlikely to be driven by compositional differences between tumor and T cell compartments. Cross‐database validation using TNMplot confirmed negative correlations between *SPRED2* and *CD8A*, *CD8B*, *GZMB*, *IFNG*, and *TNF* (Figure 8B), reinforcing the robustness of this inverse relationship.

To integrate the SPRED2‐associated T‐cell states into a single quantitative readout applicable at the cohort level, we derived a *SPRED2^high^* T‐cell gene signature from the differentially expressed genes (DEGs) upregulated in *SPRED2^high^* tumor‐infiltrating T cells (Table S2). Because tissues adjacent to tumors contain few infiltrating lymphocytes, whereas tumors are extensively infiltrated by immune cells, *SPRED2^high^* T‐cell gene signature was, as expected, higher in tumors than in adjacent normal tissues (Figure 8C), validating that the signature reflects the tumor‐infiltrating T‐cell compartment. *SPRED2* expression itself did not differ between tumors and adjacent normal tissues in pan‐BRCA (Figure 8D) but was markedly reduced in TNBC relative to luminal and HER2 BC subtypes and normal tissues (Figure 8E). Furthermore, the *SPRED2^high^* T‐cell signature was lower in Basal‐like tumors than in HER2 and luminal B (Figure 8F). Because TNBC/Basal‐like subtypes are typically enriched with T cells and associated with more favorable immunotherapy responses [34], these findings raise the possibility that the association between *SPRED2* and clinical outcome may differ according to the tumor immune landscape.

To evaluate the prognostic relevance of *SPRED2* in the context of the tumor immune landscape, we analyzed overall survival (OS) in the breast cancer cohort. *SPRED2* was not significantly associated with OS in the overall BRCA cohort by Kaplan–Meier analysis (Figure 8G and Tables S3–S5) or multivariable Cox regression adjusted for clinicopathological covariates and CD8 status (Tables S3 and S6). We therefore tested whether the association between *SPRED2* and OS differed depending on CD8 status. The primary continuous model showed a significant *SPRED2* × CD8 interaction (interaction HR = 1.25, 95% CI 1.01–1.56; *p* = 0.040; Figure 8H and Table S7a), with stratum‐specific estimates in opposite directions in CD8‐decreased and CD8‐enriched tumors (Figure 8I). Although neither stratum‐specific estimate was individually significant, the contrast between the two effects was supported by the significant interaction term. A median‐dichotomized sensitivity analysis yielded a concordant interaction (Figure S9F and Figure 8I and Table S7b). Consistent with this effect modification, median‐based Kaplan–Meier analyses showed nominally shorter OS with higher *SPRED2* expression in Basal‐like and CD8^+^ T‐cell–enriched tumors, but an opposite pattern in CD8^+^ T‐cell–decreased tumors (Figure 8J,K and Tables S4 and S5). Similarly, a higher *SPRED2^high^* T‐cell signature score showed a nominal adverse association with OS in CD8^+^ T‐cell–enriched tumors but not in CD8^+^ T‐cell–decreased tumors (Figure 8L and Tables S4 and S5). None of the six Kaplan–Meier comparisons remained significant after Benjamini–Hochberg correction (Table S4), and these analyses are therefore presented as descriptive illustrations of the interaction rather than as independent inferential findings. Together, these cohort‐level analyses indicate that *SPRED2* does not show a statistically significant cohort‐wide association with OS in breast cancer; rather, its survival association varies with the CD8^+^ T‐cell context, consistent with the inverse relationship between *SPRED2* and T‐cell effector programs observed across datasets.

## 3 Discussion

In the present study, we investigated whether SPRED2 in T cells has a role in the development of antitumor immune responses through regulation of the ERK signaling pathway, a distal signaling pathway downstream of the TCR. Using transplantable mouse BC models, we found that SPRED2 deficiency reduced primary tumor growth and endpoint lung metastatic burden, enhanced effector functions of tumor‐infiltrating CD8^+^ T cells, promoted memory‐like and T_RM_‐like CD8^+^ T cell phenotypes, and improved the antitumor activity of adoptively transferred CD8^+^ T cells. Analyses of published mouse and human datasets further showed that lower *Spred2*/*SPRED2* expression was associated with elevated effector‐like CD8^+^ T‐cell states and immune‐inflamed tumor features. In a small pembrolizumab‐treated cohort (*n* = 5 responders and 5 non‐responders), *SPRED2* expression was also lower in responder T cells, although this difference was not statistically significant at the patient level. The association between *SPRED2* expression and OS further varied depending on the CD8^+^ T‐cell states. Thus, SPRED2 may represent a candidate molecular target in CD8^+^ T cells to improve T cell‐based immunotherapies against solid tumors, including TNBC.

The degree of T‐cell activation is determined by the balance between stimulatory and inhibitory signals. Inhibitory signals mediated by ICPs expressed on the surface of T cells, such as PD‐1 and CTLA4, play an essential role in controlling inflammatory responses and protecting normal cells from T cell‐mediated cytotoxicity. T‐cell exhaustion is also driven by the upregulation of ICPs. While inhibitory pathways regulated by membrane‐associated ICPs have been extensively investigated [35], less is known about intracellular molecules that inhibit the strength of TCR signaling and downregulate the development of memory T cells. To date, more than 30 iICPs have been identified, and several phase I and II clinical trials targeting intracellular inhibitory molecules have been registered [4]. In this study, we obtained evidence indicating that SPRED2 functions as a cell‐intrinsic negative regulator of CD8^+^ T cell activation that fine‐tunes ERK activity downstream of the TCR in CD8^+^ T cells.

The strength of TCR engagement determines both the extent and duration of ERK activation. Transient vs. sustained ERK phosphorylation controls cell fate decisions toward either exclusive proliferation alone or proliferation accompanied by differentiation, respectively [36]. The ERK signaling pathway has also been implicated in anti‐apoptotic activity. For example, ERK activation suppresses the expression of the pro‐apoptotic factor Bim [37], upregulates anti‐apoptotic BCL2 family members, and promotes the degradation of pro‐apoptotic proteins, thereby enhancing cell survival [38]. *Spred2^−/−^* CD8^+^ T cells showed enhanced proliferation, reduced apoptosis, increased BCL2 expression, and higher per‐cell effector‐molecule output after TCR stimulation. Although elevated BCL2 expression is consistent with the reduced apoptosis of *Spred2^−/−^* T cells, its functional requirement for this phenotype was not directly tested. Conversely, enforced SPRED2 expression restrained GZMB accumulation, further supporting a role for SPRED2 in restraining CD8^+^ T‐cell effector‐molecule production.

We previously demonstrated by using a mouse bleomycin‐induced lung injury model that *Spred2* mRNA expression was downregulated in mouse bronchial epithelial cells during the recovery phase when epithelial cells were actively proliferating but significantly increased once the injured lung tissues healed and bronchiolar epithelialization was completed, suggesting that the modulation of *Spred2* expression is critical for the regulation of cell proliferation of primary cells [11]. In the present study, we showed that *Spred2* mRNA levels transiently increased after TCR activation, but subsequently decreased in both mouse and human T cells in vitro. Furthermore, the expression of *SPRED2* was lower in tumor‐infiltrating T cells from TNBC patients who responded to immunotherapy than in those from non‐responders, although at the patient level this difference did not reach statistical significance. These findings are consistent with the hypothesis that downregulation of *SPRED2* endows CD8^+^ T cells with enhanced effector function and an increased propensity to acquire memory‐like phenotypes upon TCR activation. The mechanisms regulating the transcription of the *SPRED2/Spred2* gene in epithelial cells or T cells remain uncharacterized. It will be important to elucidate how activation of growth factor receptors on epithelial cells or TCR on T cells leads to the downregulation of *SPRED2* expression in each cell type to find a way to control their proliferation or activation by manipulating its expression.

To determine whether the enhanced antitumor phenotype could reflect pre‐existing differences in T‐cell development or homeostasis, we examined tumor‐free *Spred2^−^* ^/^ *^−^* mice. No detectable alterations were observed in thymic T‐cell development (Figure S10) or peripheral T‐cell homeostasis (Figure S11) compared with WT mice, arguing against baseline differences in T‐cell composition as the basis for the enhanced antitumor phenotype. Our data indicate that the loss of SPRED2 in CD8^+^ T cells is a major driver of the enhanced antitumor immunity; however, other hematopoietic compartments may still contribute in more subtle ways. Among CD4^+^ T cells, *Spred2^−/−^* cells displayed a Th1‐skewed differentiation profile with reduced Th2 and Th17 responses upon anti‐CD3e/CD28 mAbs and PMA/ionomycin stimulation, together with increased IL‐2 production, a cytokine critical for T‐cell survival (Figure S12A–C) [39]. Such a Th1‐oriented CD4^+^ compartment would be expected to reinforce, rather than counteract, the enhanced CD8^+^ T‐cell response. Importantly, the regulatory T‐cell compartment was unaffected within the 4T1 TME: the intratumoral frequencies of Foxp3^+^, CD25^+^, and Foxp3^+^CD25^+^ T cells (% of CD4^+^) were comparable between WT and *Spred2^−^* ^/^ *^−^* mice (Figure S12D), indicating that the antitumor phenotype is not attributable to a reduction in regulatory T cells. Beyond lymphoid cells, SPRED2 in macrophages may influence antigen presentation, chemokine production (e.g., CXCL9/10), and TAM polarization by inhibiting ERK activation, thereby indirectly shaping CD8^+^ T‐cell recruitment and persistence within tumors [40, 41]. We have previously shown that SPRED2 restrains ERK‐driven inflammatory outputs in mouse macrophages under specific stimuli, such as heightened TNFα/CCL2 production in response to lipotoxic signals [42], and that SPRED2 deficiency exacerbates lipopolysaccharide‐induced lung inflammation via increased ERK signaling [43]. However, neither myeloid‐specific *Spred2* deletion nor TAM depletion reproduced the reduction in endpoint lung metastatic burden, and global SPRED2 loss did not grossly alter TAM abundance or M1/M2 polarization. The dissociation between the lack of effect of myeloid‐specific *Spred2* deletion and the tumor‐suppressive effect of TAM depletion in *Spred2^−/−^* hosts suggests that TAMs contribute to primary tumor growth through mechanisms independent of macrophage‐intrinsic SPRED2, likely by suppressing CD8^+^ T‐cell activity in the TME. This clodronate‐based approach does not, however, exclude contributions from other phagocytic populations, such as dendritic cells, which may themselves shape CD8^+^ T‐cell responses within the TME. By contrast, depletion of CD8^+^ T cells with anti‐CD8 mAb abrogated both the suppression of primary tumor growth and the reduction in endpoint lung metastatic burden in *Spred2^−/−^* mice. Together, these findings identify CD8^+^ T cells as the principal effector population responsible for both phenotypes, although future studies using CD8^+^ T cell‐specific *Spred2^−/−^* mice will be required to provide more definitive evidence.

There are several limitations in this study. First, our analyses of public datasets remain correlative and require further validation to establish clinical relevance. In SCAN‐B, limited subgroup event numbers and loss of significance after multiple‐testing correction warrant cautious interpretation of the stratified survival analyses, which primarily illustrate the observed *SPRED2* × CD8 interaction. Second, although we investigated the role of SPRED2 in CD8^+^ T cells, CD4^+^ T cells, and macrophages, we did not examine SPRED2 deficiency in other leukocyte populations, including DCs, NK cells, and myeloid‐derived suppressor cells. For example, DC antigen‐presenting activity may also be enhanced in the absence of SPRED2. Although the intratumoral frequencies of these cell populations did not differ significantly between WT and *Spred2^−^* ^/^ *^−^* mice, their cell‐intrinsic functions were not assessed, and their contributions to the observed antitumor phenotype cannot be excluded. Third, tumor‐reactive CD8^+^ T cells were inferred using the surrogate phenotype CD11a^high^PD‐1^+^ rather than antigen‐specific tetramers. This approach enriches for antigen‐experienced, putatively tumor‐reactive cells but does not definitively identify tumor antigen‐specific CD8^+^ T cells or exclude bystander activation. This caveat also applies to the adoptive‐transfer and in vitro killing experiments. The killing assays used polyclonally activated CD8^+^ T cells without MHC class I blockade or an antigen‐specific readout and therefore measure cytotoxic capacity against 4T1 cells under the assay conditions rather than MHC class I‐restricted, tumor antigen‐specific killing. Consistent with this limitation, CD8^+^ T cells from tumor‐naive mice also killed 4T1 targets (Figure S4E,F). The greater antitumor activity of CD8^+^ T cells from tumor‐bearing donors (Figure 5G,H) may reflect enrichment of tumor‐reactive cells, differences in differentiation states, or both. Direct characterization of tumor antigen‐specific CD8^+^ T cells using TCR clonotype tracking or antigen‐specific reagents remains an important direction for future studies. Fourth, because primary tumor burden also differed between genotypes in the 4T1 model, the reduced endpoint lung metastatic burden in *Spred2^−/−^* mice cannot be interpreted independently of primary tumor growth. Our endpoint analysis also cannot distinguish effects on tumor‐cell dissemination or lung seeding from effects on subsequent metastatic outgrowth, and the observed differences in metastatic nodule size cannot be attributed to any one of these steps. Fifth, memory‐like and T_RM_‐like populations were defined phenotypically, without functional recall or tissue‐residency validation. Thus, these findings indicate memory‐associated phenotypes rather than functionally established durable memory or true tissue residency.

Beyond these limitations, several important directions emerge for future investigation. In particular, whether SPRED2 deletion in CD8^+^ T cells synergizes with established ICP inhibitors—including anti‐PD‐1, anti‐PD‐L1, and anti‐CTLA4 mAbs—warrants further exploration. In 4T1 tumors, as noted above, PD‐1^+^CD8^+^ T cells were expanded in *Spred2^−/−^* tumors and retained granzyme B expression (Figure S2). Because PD‐1 marks both recent activation and exhaustion, and functional exhaustion was not assessed, these cells cannot be assigned to either state based on these markers alone. The persistence of PD‐1 expression on granzyme B‐expressing *Spred2^−/−^* CD8^+^ T cells provides a rationale for combining SPRED2‐targeted T‐cell therapy with PD‐1/PD‐L1 blockade. A more comprehensive profiling of checkpoint expression on peripheral immune cells vs. tumor‐associated T cells in SPRED2‐deficient hosts will be an important next step to fully evaluate this combination strategy.

The field of cancer immunotherapy has advanced rapidly, particularly in restoring T cell‐mediated immune responses. Strategies such as ICP blockade and adoptive cell therapy have shown success in treating refractory solid tumors and achieving long‐term remission in patients [5]. One of the most promising approaches is the genetic engineering of T cells to enhance their antitumor capabilities. Gene editing technologies, such as CRISPR/Cas9, have been used to upregulate critical genes or disrupt inhibitory genes, thereby improving the efficacy of engineered T cells [44, 45]. A major focus in this area is the deletion of negative regulatory factors to counteract T‐cell exhaustion and enhance their persistence and functionality within the TME. For instance, deletion of diacylglycerol kinases in mice and human CAR T cells increased T‐cell activation and tumor clearance by enhancing ERK activation [46, 47]. Knockout of the *Rasa2* gene in CD8^+^ TILs enhanced their responsiveness to TCR stimulation by augmenting the RAS‐ERK signaling cascade and improved antigen sensitivity, effector function, and tumor control [48]. Moreover, knockout of the *FAM49B* gene (also known as the *CYRIB* gene) or disruption of the FAM49B‐Rac interaction indirectly promoted ERK‐associated downstream signaling, thereby enhancing T‐cell activation and the antitumor effects of CD8^+^ T cells [49]. Our study adds SPRED2 to this conceptual framework and suggests that SPRED2 targeting may represent a new strategy to improve CD8^+^ T‐cell function, persistence, and antitumor activity in solid tumors.

## 4 Experimental Section

### 4.1 Cell Lines, Culture Conditions, and Cell‐Isolation Procedures

Detailed information is provided in the Supplementary Methods.

### 4.2 Mice

The generation of *Spred2^−/−^* mice, *Spred2Tg* mice and LysM‐Cre *Spred2* ^fl/fl^ mice on the C57BL/6J background was previously described [13, 50]. To generate *Spred2^−/−^* on a BALB/c background, we crossed *Spred2^−/−^* mice on the C57BL/6J background with BALB/c mice. The resulting F1 heterozygous offspring were then backcrossed to BALB/c WT mice. After 10 generations of backcrossing, we obtained *Spred2^−/−^* mice on a BALB/c background. The following primer sets were used to genotype the mice: *Spred2*WT(KO)/F: 5’‐CCAGGTTCCGCTCACAACTA‐3’; *Spred2*WT/R: 5’‐GCGCACGATGGAGCTCGCGTCTTC‐3’ and *Spred2*KO (Neo3’‐1): 5’‐ CGAGATCAGCAGCCTCTGTTCCACATAC‐3’. C57BL/6J or BALB/c mice were used as WT mice. All mice used in this study were bred and maintained under a continuous 12‐h light/12‐h dark cycle under specific pathogen‐free conditions at the Department of Animal Resources, Okayama University (Okayama, Japan). Unless otherwise indicated, female mice aged 6–8 weeks at the start of each experiment were used. Age‐matched mice were used for comparisons between genotypes. Only female mice were used in this study, reflecting the clinical relevance of breast cancer as a predominantly female disease. This decision was further supported by the fact that all patients included in this study were also female, enabling a model that more accurately reflects the hormonal and physiological context of human breast cancer.

### 4.3 Mice Experiments

For the tumor transplantation model, one million tumor cells were seeded in a T‐75 tissue culture flask and grown to 50%–80% confluence. Cells were detached with 0.2% trypsin‐EDTA (Gibco, Carlsbad, CA, USA), washed once with complete medium and three times with PBS, and resuspended in PBS at 5  ×  10^5^ cells/mL (4T1 cells) or 1  ×  10^6^ cells/mL (EO771 cells). 4T1 or EO771 cells in 100 µL PBS were injected into the third left mammary fpad. Tumor length and width were measured using a caliper, and tumor volume was calculated using the following formula: Volume  =  (width)^2^ × length/2. Tumor tissues were excised and fixed in 10% neutral buffered formalin (NBF) for histological examination. To evaluate the presence of lung metastases, the lungs were perfused with Bouin's solution (Wako, Osaka, Japan), fixed in the same solution, and then the number of tumor nodules was counted by eye. After fixation in Bouin's solution, subpleural lung surface metastases were easily identified by their bright, white appearance. To minimize observer bias, the lung specimens were anonymized, and metastatic nodules were counted independently by a blinded observer. Closely adjacent lesions were counted as separate nodules only when distinct boundaries were visible. For the CD8^+^ T cell adoptive transfer model, CD8^+^ T cells were isolated from the spleens of naive or 4T1 tumor‐bearing mice as described above. CD8^+^ T cells from 4T1 tumor‐bearing mice were further expanded by culturing at 37°C in RPMI 1640 medium containing 10% FBS with anti‐CD3e/CD28 mAbs and 10 ng/mL recombinant murine IL‐2. After culturing for 3 days, the cells were washed three times with PBS and resuspended in PBS. Five million WT or *Spred2^−/−^* CD8^+^ T cells were injected into the tail vein of WT mice 7, 14, and 21 days after tumor cell inoculation. Five to 10 mice were used per group; the exact number for each experiment is provided in the corresponding figure legend. Tumor size was measured with calipers three times per week. Tumors and lungs were harvested at the end of each experiment. For experiments involving treatment allocation within the same genotype, mice were randomly assigned to experimental groups. Genotype‐based comparisons were determined by the animals’ genotype and therefore were not subject to random allocation. Experimental protocols used in this study were reviewed and approved by the Animal Care and Use Committee at Okayama University, Okayama, Japan (OKU‐2020446, OKU‐2023216, OKU‐2023621, OKU‐2024461).

### 4.4 Histological Analysis

NBF‐fixed lung samples were embedded in paraffin. Thin sections were prepared and stained with hematoxylin and eosin (H&E) to show representative images of the lungs. Tumors were fixed in 10% NBF overnight and embedded in paraffin. Sections were prepared, and IHC was performed manually using a conventional method: Briefly, sections were deparaffinized in xylene and rehydrated in descending concentrations of ethanol. Endogenous peroxidase reactivity was blocked with 3% H_2_O_2_ for 10 min. For antigen retrieval, sections were immersed in 10 mm citrate buffer (pH 6.0) or 5 mm EDTA solution (pH 8.0) and microwaved (700 W) continuously for 15 min in a pressure cooker and then incubated with the appropriate primary antibody for 1.5 h at room temperature. After washing, sections were incubated with an appropriate secondary antibody conjugated with horseradish peroxidase (Nichirei, Tokyo, Japan), and the signals were visualized using DAB (Dako, Santa Clara, CA) according to the manufacturer's instructions. Finally, sections were counterstained with hematoxylin, dehydrated, and mounted. Images were captured using an Olympus BX43 light microscope connected to a DP73 digital camera (Olympus). Antibodies used for IHC are listed in Table S8. To count the number of CD3, CD4, and CD8 positive T cells, we randomly captured at least 4–6 images in each section at 40 × or 20 × magnification. We conducted quantitative analyses using QuPath 0.4.2, open‐source software [51], and trained cell classifiers based on previous studies to specifically quantify CD3, CD4, and CD8 positive TILs, and other negative cells. For each image, TIL measurements were analyzed using the following constructed variables: positive cells (% of total cells) = 100 × (# of TILs / # of total cells), representing the proportion of TILs (CD3^+^, CD4^+^, or CD8^+^ T cells) relative to all detected cells. Image acquisition and quantitative analysis were performed with sample identity masked to the investigator.

### 4.5 Macrophage Depletion

Fifty µL of clodronate or control liposomes (FormuMax Scientific, Sunnyvale, CA, USA) was intraperitoneally injected into mice one day before 4T1 inoculation (day ‐1) and then every 5 days from day 7 to day 22. Tumors and lungs were harvested on day 28. Depletion efficiency was confirmed by flow cytometry.

### 4.6 CD8^+^ T‐Cell Depletion

One hundred µg of anti‐CD8 mAb (Lyt‐2.2) or rat IgG2b κ isotype control antibody (MedChemExpress, HY‐P990682) was intraperitoneally injected into mice every 5 days from day 7 to day 22. Tumors and lungs were harvested on day 28. Depletion efficiency was verified by flow cytometry.

### 4.7 Quantitative Real‐Time PCR (RT‐qPCR)

For gene expression analysis, total RNA was extracted from cells and tumor tissues using the High Pure RNA Isolation Kit (Roche, Mannheim, Germany) or TRIsure reagent (Nippon Genetics, Tokyo, Japan). Complementary DNA (cDNA) was synthesized using the High‐Capacity cDNA Reverse Transcription Kit (ThermoFisher, Waltham, MA). Real‐time qPCR was performed with the StepOnePlus system (ThermoFisher). The expression of the genes was analyzed using the TaqMan gene expression assay (Applied Biosystems, Foster City, CA). The primers used in this study are listed in Table S9. The total volume of each reaction was 10 µL containing 5.2 µL TaqMan ProAmp Master Mix reagent, 0.4 µL primer, and 2.8 µL UltraPure DNase/RNase‐free distilled water (Invitrogen, Carlsbad, CA). The expression level of each gene was normalized to that of the *Gapdh* gene and expressed as fold change over control gene expression.

### 4.8 ELISA

Detailed procedures are provided in the Supplementary Methods.

### 4.9 Flow Cytometry

After Fc blocking with anti‐CD16/CD32 antibody (BioLegend, 101302), cells were incubated with fluorescently labeled antibodies of interest or isotype control rat IgG. For intracellular cytokine staining, cells were stimulated with PMA plus ionomycin in the presence of brefeldin A (BioLegend) for 6 h before staining with antibodies to surface proteins, followed by fixation and permeabilization (BioLegend, 424401) and staining with antibodies to intracellular antigens. Cell populations were examined using a CytoFLEX S flow cytometer (Beckman Coulter, Inc., Brea, CA), and data were analyzed using FlowJo X. Dead cells were excluded based on the viability dye staining (7AAD or Zombie Yellow Fixable Viability Kit, BioLegend). Cells were sequentially gated to exclude debris, doublets, and dead cells before identification of the indicated immune‐cell populations, and the same gating strategy was applied consistently across samples within each experiment. Representative gating strategies are provided in Figure S13. Fluorescence compensation was established using single‐stained controls acquired under the same instrument settings as the experimental samples and was verified before downstream analysis. Antibodies used for flow cytometry are listed in Table S10.

### 4.10 T‐Cell Proliferation, Apoptosis Assays and CFSE‐Based In Vitro Cytotoxicity Assay

Detailed procedures are provided in the Supplementary Methods.

### 4.11 Competitive Co‐Transfer

CD8^+^ T cells were isolated from naive WT and *Spred2^−/−^* mice and activated in vitro with anti‐CD3e/CD28 mAbs for 3 days. Activated WT and *Spred2^−/−^* CD8^+^ T cells were labeled with CFSE and CTV, mixed at 1:1 (5 × 10^6^ cells of each genotype); the input ratio was confirmed by flow cytometry. The mixture was transferred into WT mice that had received 4T1 cells 10 days earlier. Three days later, spleens and tumors were collected, and the recovery of CFSE^+^ (WT) and CTV^+^ (*Spred2^−/−^*) cells among CD8^+^ T cells was quantified by flow cytometry. The same experiment was done by swapping the labels.

### 4.12 Western Blotting

For total protein isolation, cells were lysed in a lysis buffer (Cell Signaling, Danvers, MA) containing protease inhibitor cocktail (Roche) and Halt phosphatase inhibitor cocktail (ThermoFisher), incubated on ice for 30 min, and then centrifuged at 14,500 × *g* for 10 min. Tumor tissues were lysed in RIPA buffer containing phenylmethanesulfonyl fluoride. Protein concentrations were measured by BCA protein assay (TaKaRa, Kusatsu, Shiga, Japan). Equal amounts of protein samples were denatured at 100°C for 10 min with 4 × NuPAGE LDS sample buffer and 10  ×  sample reducing agent (Invitrogen). Proteins (15–30 µg) were separated on 4%–12% NuPAGE Bis‐Tris precast gels (ThermoFisher) and transferred onto nitrocellulose blotting membranes (GE Healthcare Life Sciences, Freiburg, Germany). The membranes were blocked with 5% milk in Tris‐buffered saline‐Tween 20 (TBS‐T) for 1 h at room temperature. After overnight incubation with a primary antibody at 4°C, the membrane was washed three times with TBS‐T for 10 min and then incubated with horseradish peroxidase‐conjugated secondary antibodies for 1 h at room temperature. After secondary antibody incubation, the membranes were washed three times with TBS‐T for 10 min. The target proteins were visualized using ImmunoStar LD (Wako, Osaka, Japan), and the membranes were scanned using a C‐DiGit blot scanner (LI‐COR Biotechnology, Lincoln, NE). The blot images were semi‐quantitated using Image Studio software. The antibodies used for Western blotting are listed in Table S11.

### 4.13 Transcriptomic and Bioinformatic Analyses

We analyzed publicly available bulk and single‐cell RNA‐seq (scRNA‐seq) datasets of human and mouse CD8^+^ T cells to characterize SPRED2‐linked transcriptional programs. scRNA‐seq data were processed with CellRanger/Seurat to define *SPRED2^high^*/*SPRED2^low^* T‐cell signatures. Bulk RNA‐seq data were trimmed, aligned, quantified, and batch‐corrected using standard pipelines. Differential expression, trajectory, and enrichment analyses used DESeq2, EBSeq‐HMM, clusterProfiler, and MSigDB. Cross‐cohort correlation, subtype, and survival analyses were performed via LinkedOmics, TIMER 3.0, UCSC Xena, UALCAN, TNMplot, and Kaplan–Meier Plotter. PPI networks were generated through STRING/Cytoscape. Comprehensive descriptions of data preprocessing, statistical analyses, computational parameters, and software settings used across all pipelines are provided in the Supplementary Methods.

### 4.14 Statistical Analysis

Data analysis and graphical representation were performed in GraphPad Prism 10 software (San Diego, CA, USA) or R (version 4.5.0) and edited for appearance using Adobe Illustrator (Adobe). DEGs of each scRNA‐seq cluster were identified using Seurat FindAllMarkers function with the normalized gene expression (SCTransform v2) and the default test setting, which is the non‐parametric Wilcoxon Rank Sum test following adjusted *p*‐value based on Bonferroni correction using all genes in the dataset. Unless otherwise indicated, no additional data points were excluded. Data were pooled only when experiments were performed under the same experimental conditions. The biological replicate was defined according to the experimental unit. For in vivo experiments, each mouse represented one biological replicate. For ex vivo experiments using primary mouse cells, independently isolated cells from individual mice represented biological replicates. Technical replicate wells or repeated measurements from the same biological sample were not treated as independent biological replicates. The number of biological replicates (n) is provided in the corresponding figure legends. After assessments of the normality of data, statistical tests were performed accordingly. Paired two‐tailed t tests were used for competitive co‐transfer experiments, in which both genotypes were recovered from the same recipient. Dunnett's post‐hoc test was used for comparisons against a single reference time point. Multiple *t* tests were corrected using the Holm–Šídák's or Šídák's method as indicated in the figure legends. Survival analyses (Kaplan–Meier with log‐rank tests and multivariable Cox proportional hazards models), partial Spearman correlations, and Benjamini–Hochberg false discovery rate correction are described in detail in the Supplementary Methods. Data are displayed as mean ± standard error of the mean (SEM) or 95% confidence intervals unless indicated otherwise. A value of *p* < 0.05 was considered statistically significant, adopting the following convention: ∗*p* < 0.05, ∗∗*p* < 0.01, ∗∗∗*p* < 0.001; ns, not significant.

## Author Contributions

M.T. participated in the experimental design, performed most of the experiments, analyzed the data, and drafted the original manuscript. T.Y. provided guidance on the experimental design, contributed to data analysis, and revised the manuscript. K.Z. co‐analyzed all sequencing datasets. C.L. and T.G. provided technical support. M.F. and T.O. offered advice on study design and data interpretation. Z.W. developed the sequencing analysis software and performed transcriptomic and bioinformatic analyses. A.M. supervised the study, provided resources, secured funding, and reviewed and finalized the manuscript.

## Funding

This work was supported in part by Japan Society for the Promotion of Science: 25293095, 21H02988, and 22K19562.

## Conflicts of Interest

The authors declare no conflicts of interest.

## Supporting information




**Supporting File 1**: advs77909‐sup‐0001‐SuppMat.pdf.

**Supporting File 2**: advs77909‐sup‐0002‐SuppMat.docx.

**Supporting File 3**: advs77909‐sup‐0003‐SuppMat.docx.

## Data Availability

Public transcriptomic datasets are available from GEO (GSE89307, GSE247647, GSE234099, GSE246613, and GSE96058). All human data analyzed in this study were obtained from publicly available, de‐identified datasets; ethical approval and informed consent were obtained in the original studies. TCGA‐BRCA data are available via LinkedOmics (https://www.linkedomics.org/login.php), TIMER 3.0 (https://compbio.cn/timer3/), TNMplot (https://tnmplot.com/analysis/), UCSC Xena (https://xena.ucsc.edu/), and UALCAN (https://ualcan.path.uab.edu/index.html). PPI and GSEA resources are available through STRING (https://cn.string‐db.org/) and MSigDB (https://www.gsea‐msigdb.org/gsea/msigdb/).
