# Supplementary material for: SPRED2 Negatively Regulates CD8+ T Cell‐Mediated Antitumor Immunity in Breast Cancer
Source: Adv Sci (Weinh). 2026 Sep 27:e77909. Online ahead of print. doi: 10.1002/advs.77909 (PMC13616240; doi:10.1002/advs.77909)
Supplement: Supplementary file 1 [file ADVS-9999-e77909-s002.pdf]

Supporting Information

SPRED2 Negatively Regulates CD8^{+} T Cell-mediated Antitumor Immunity in Breast Cancer

Miao Tian[1], Teizo Yoshimura[1], Kun Zhao[2], Chunning Li[1], Tong Gao[1], Ziyi Wang[3], Masayoshi Fujisawa[1], Toshiaki Ohara[1], Akihiro Matsukawa[1]**

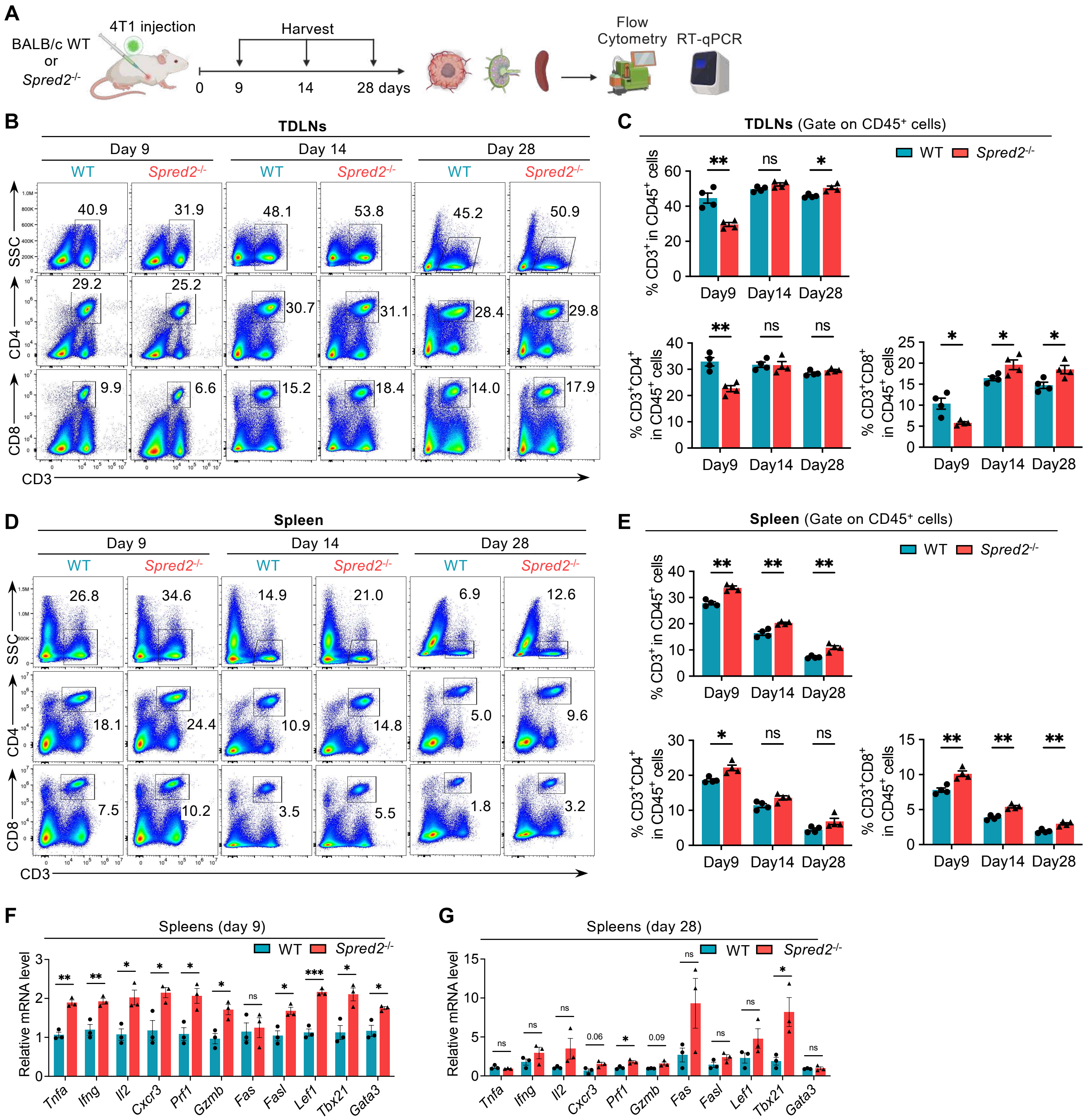

Figure S1. SPRED2 deficiency expands T cells in secondary lymphoid organs during 4T1 tumor progression. A-G) 4T1 cells (5×10^4 in 100 μL PBS) were injected into the mammary fat pad of WT or *Spred2*$^{-/-}$ mice; TDLNs and spleens were analyzed at the indicated time points (n = 3 per group). (A) Experimental design; (B, D) Flow cytometry of CD3$^+$, CD4$^+$, and CD8$^+$ T cells in TDLNs (B) and spleens (D); (C, E) Frequency of each cell population as a percentage of CD45$^+$ cells in TDLNs (C) and spleens (E); (F, G) Splenic immune-gene expression by RT-qPCR on day 9 (F) and day 28 (G). Data are presented as mean ± SEM. Statistics: (C, E) two-way ANOVA followed by Šídák's multiple-comparisons test; (F, G) multiple unpaired t-tests with Šídák's correction. *$p < 0.05$, **$p < 0.01$; ns, not significant.

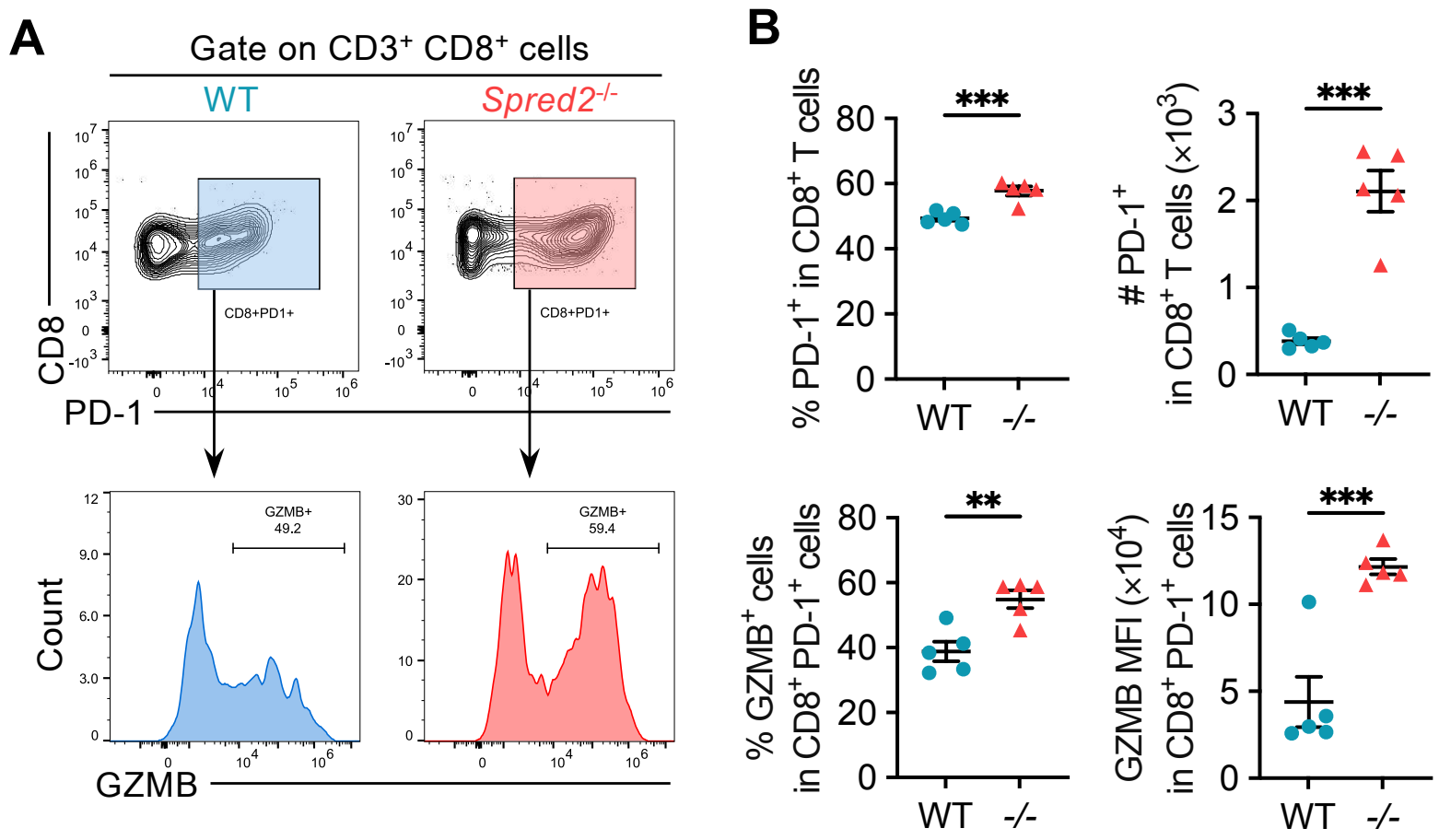

Figure S2. Spred2 deficiency increases GZMB expression in PD-1$^+$ tumor-infiltrating CD8$^+$ T cells. 4T1 cells (5×10^4 in 100 μL PBS) were injected into WT or *Spred2*$^{-/-}$ mice; tumors were harvested on day 28 and analyzed by flow cytometry. (A) Representative CD8 vs PD-1 contour plots (gated on CD3$^+$CD8$^+$). (B) Frequency (left) and number (right) of PD-1$^+$CD8$^+$ T cells (upper panel); GZMB$^+$ frequency (left) and MFI (right) within this subset (lower panel) (n = 5 per group). Data are presented as mean ± SEM. Statistics: (B) unpaired two-tailed Student's t-test. $^{**}p < 0.01$, $^{***}p < 0.001$.

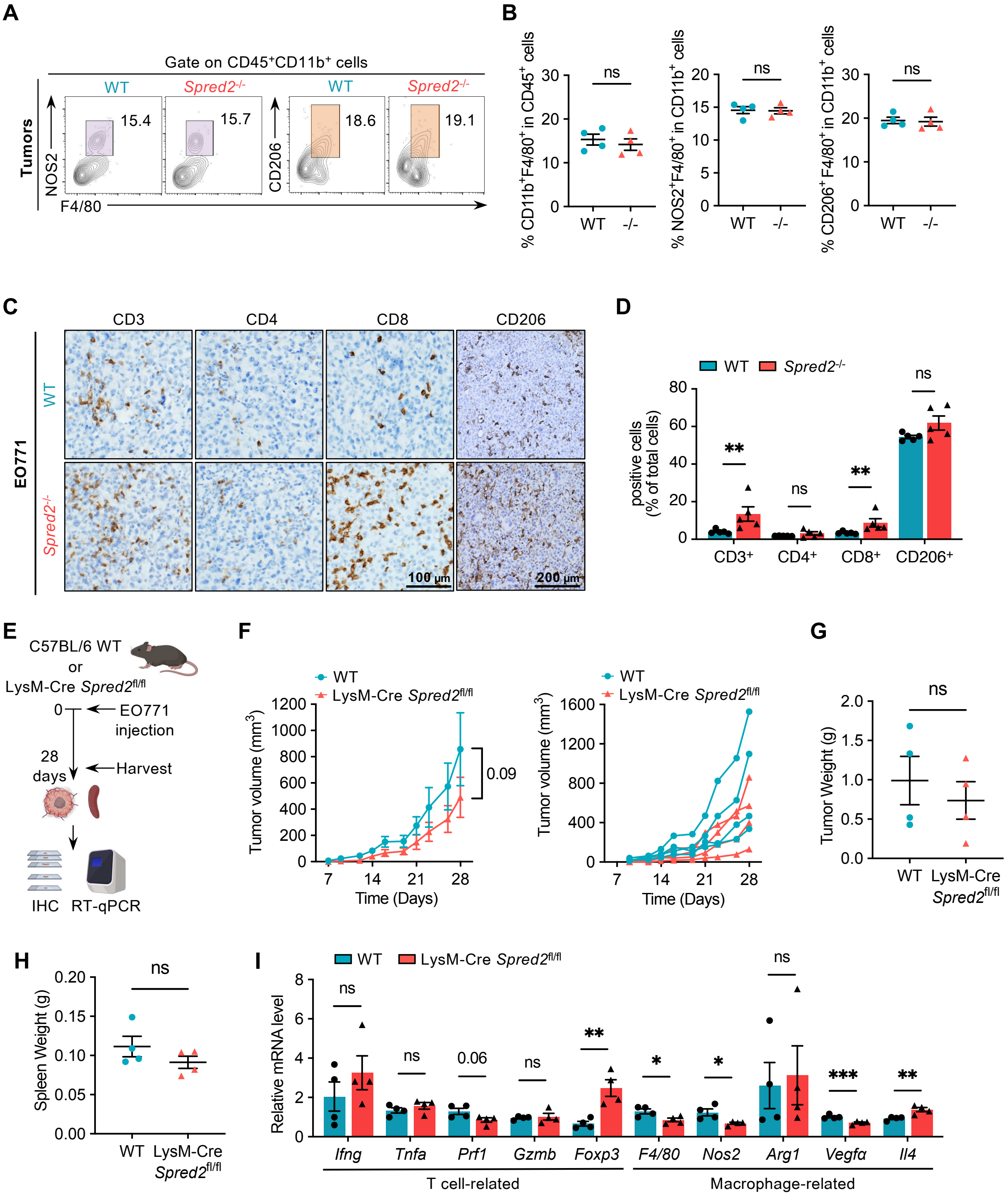

Figure S3. Myeloid-specific SPRED2 deletion does not recapitulate the antitumor phenotype of systemic SPRED2 deficiency. A, B) 4T1 cells (5×10^4 in 100 µL PBS) were injected into the mammary fat pad of WT or *Spred2*-/- mice, and the primary tumors were resected on day 28. TAMs were assessed by flow cytometry. The expression of each marker was analyzed under the gating conditions shown. C, D) EO771 cells (1×10^5 in 100 µL PBS) were injected into the mammary fat pad of C57BL/6J WT or *Spred2*-/- mice; primary tumors were harvested on day 28 for immunohistochemistry. (C) Representative images; (D) QuPath quantification (% positive cells; n = 5 per group). E-I) EO771 cells (1×10^5 in 100 µL PBS) were injected into the mammary fat pad of WT or LysM-Cre Spred2$^{fl/fl}$ mice; primary tumors and spleens were analyzed on day 28 (n = 4 per group). (E) Experimental design; (F) Tumor growth (left, means; right, individual trajectories); (G–I) Day-28 analysis: (G) primary tumor weight; (H) spleen weight; (I) tumor immune-gene expression by RT-qPCR. Data are presented as mean ± SEM. Statistics: (B, G, H) unpaired two-tailed t-test; (D, I) multiple unpaired t-tests with Šídák's correction; (F) repeated-measures two-way ANOVA with Šídák's multiple-comparisons test. No statistical analysis was performed for schematic or representative image panels. *$p < 0.05$, **$p < 0.01$, ***$p < 0.001$; ns, not significant.

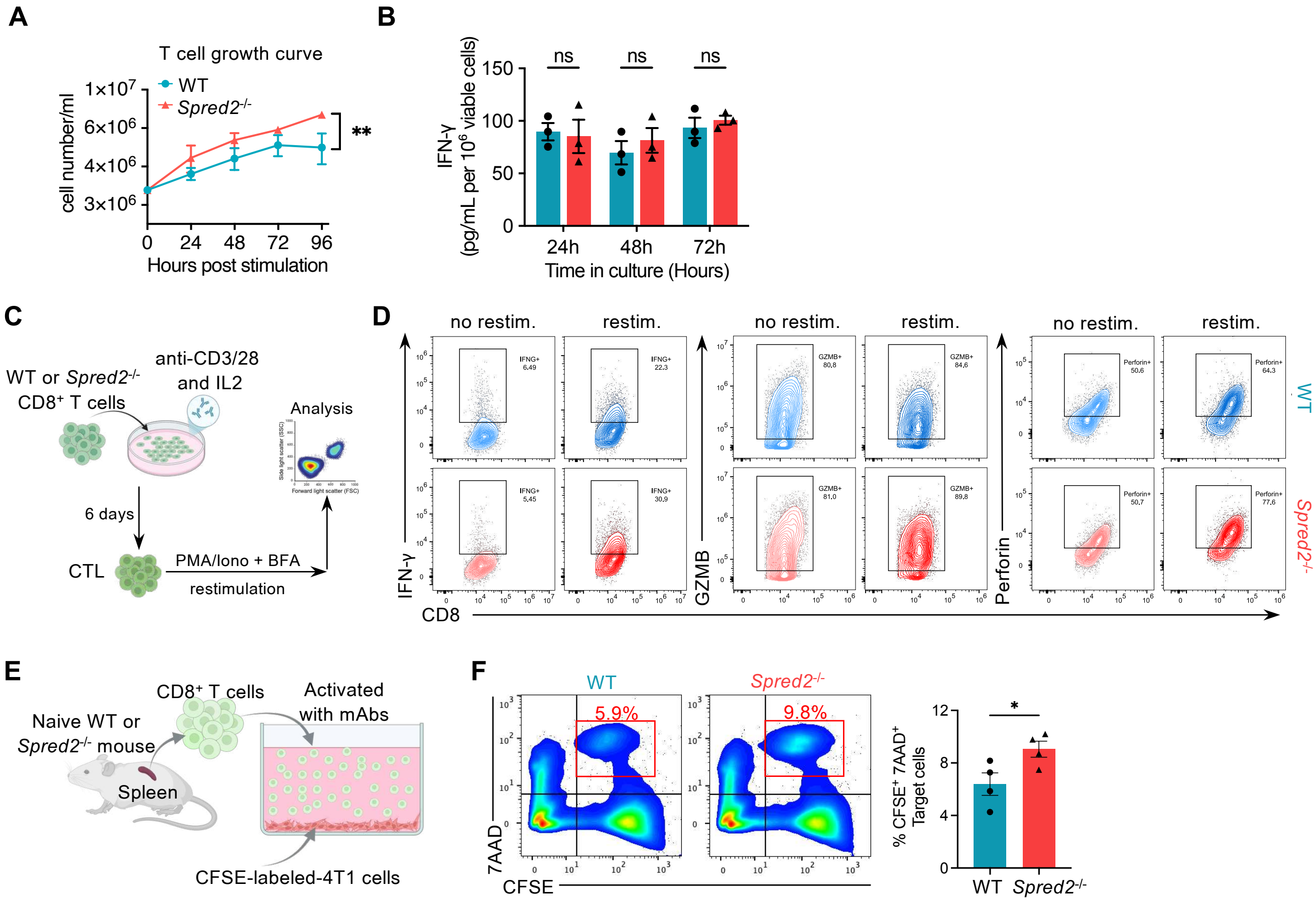

Figure S4. SPRED2 deficiency enhances T-cell expansion and cytotoxic function in vitro. (A) Splenic T cells from naive WT or *Spred2*$^{-/-}$ mice were activated with anti-CD3e/CD28 mAbs; T cell numbers were counted over time (n = 3 biological replicates). (B) IFN-γ production by unstimulated naive CD8^{+} T cells over time by ELISA (n = 3 biological replicates). C, D) Naive CD8^{+} T cells from WT or *Spred2*$^{-/-}$ mice were activated with anti-CD3e/CD28 mAbs + IL-2, differentiated into CTLs over 6 days, and restimulated with PMA/ionomycin + BFA. (C) Experimental design; (D) Representative contour plots (marker vs CD8) for IFN-γ, GZMB, and perforin in WT (upper panel) and *Spred2*$^{-/-}$ (lower panel) CTLs, with (restim.) and without (no restim.) restimulation (n = 3 biological replicates). E, F) CD8^{+} T cells from naive WT or *Spred2*$^{-/-}$ mice were activated for 48 hours, then co-cultured with CFSE-labeled 4T1 targets for 48 hours (effector: target = 10: 1). (E) Experimental design; (F) Quantification of CFSE^{+}7AAD^{+} target cells by flow cytometry (n = 4 per group). Data are presented as mean ± SEM. Statistics: (A) two-way repeated-measures ANOVA followed by Šídák's multiple-comparisons test; (B) two-way ANOVA followed by Šídák's multiple-comparisons test; (F) unpaired two-tailed Student's t-test. *$p < 0.05$, **$p < 0.01$; ns, not significant.

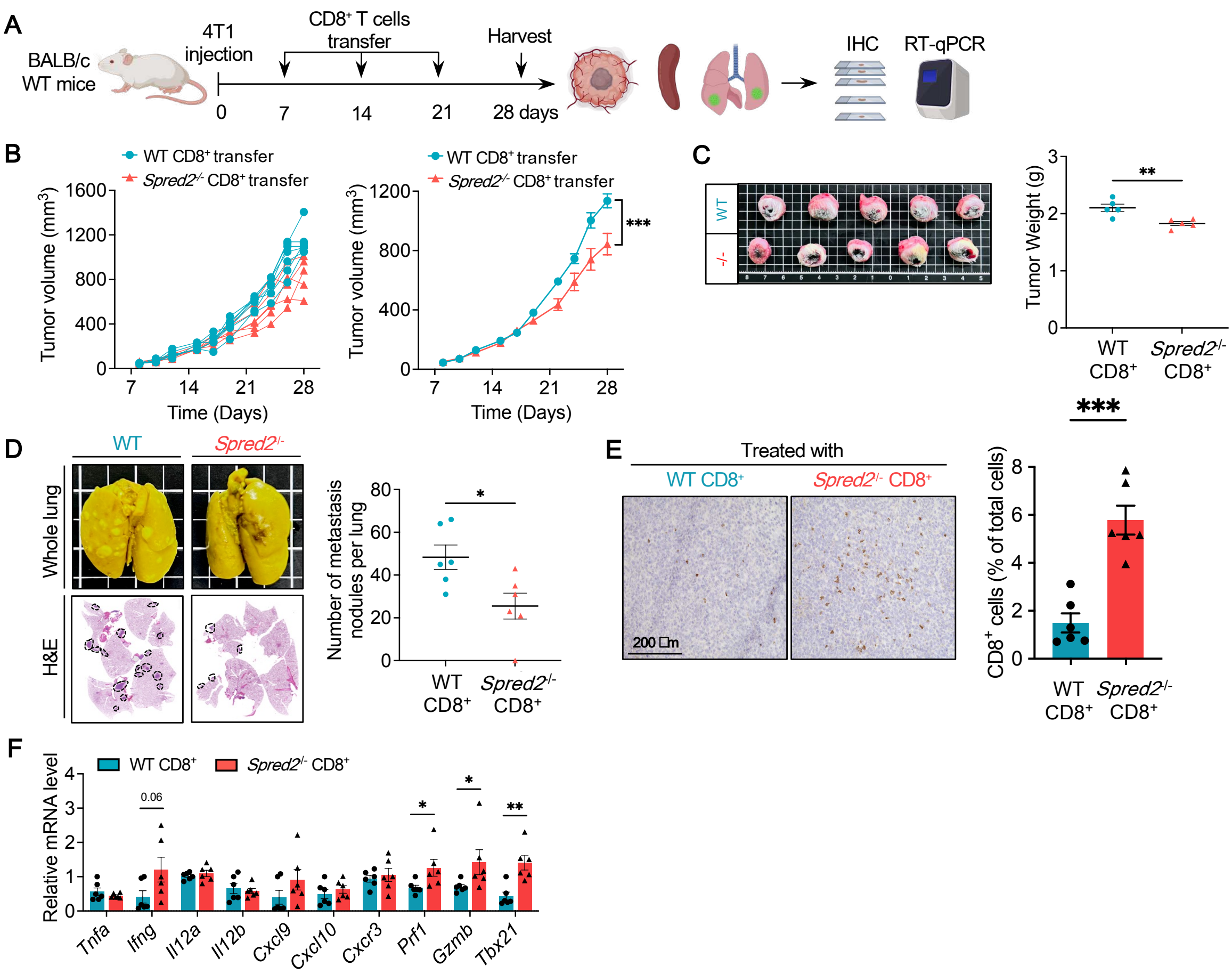

Figure S5. Naive *Spred2-/-* CD8+ T cells show enhanced antitumor activity after adoptive transfer. A-F) 4T1 cells (5×10^4 in 100 μL PBS) were injected into WT recipients; splenic CD8+ T cells from naive WT or *Spred2-/-* donors were expanded with anti-CD3e/CD28 mAbs + IL-2 for 3 days and transferred on days 7, 14, and 21 (5×10^6 cells in 100 μL PBS/infusion). (A) Experimental design; (B) Tumor growth (left, individual trajectories; right, means); (C) Tumor photos (left) and weight on day 28 (right; n = 5 per group); (D) Left, lungs fixed in Bouin's solution (upper panel) and H&E-stained (lower panel); Right, metastatic nodule count (n = 6 per group). (E) Tumor-infiltrating CD8+ T cells by immunohistochemistry on day 28: representative images (left) and QuPath quantification (right; n = 6 per group); (F) Tumor immune-gene expression by RT-qPCR (n = 6 per group). Data are presented as mean ± SEM. Statistics: (B) two-way repeated-measures ANOVA followed by Šídák's multiple-comparisons test; (C, E) unpaired two-tailed Student's t-test; (D) two-tailed Mann–Whitney U test; (F) multiple unpaired t-tests with Šídák's correction. *$p < 0.05$, **$p < 0.01$, ***$p < 0.001$.

A

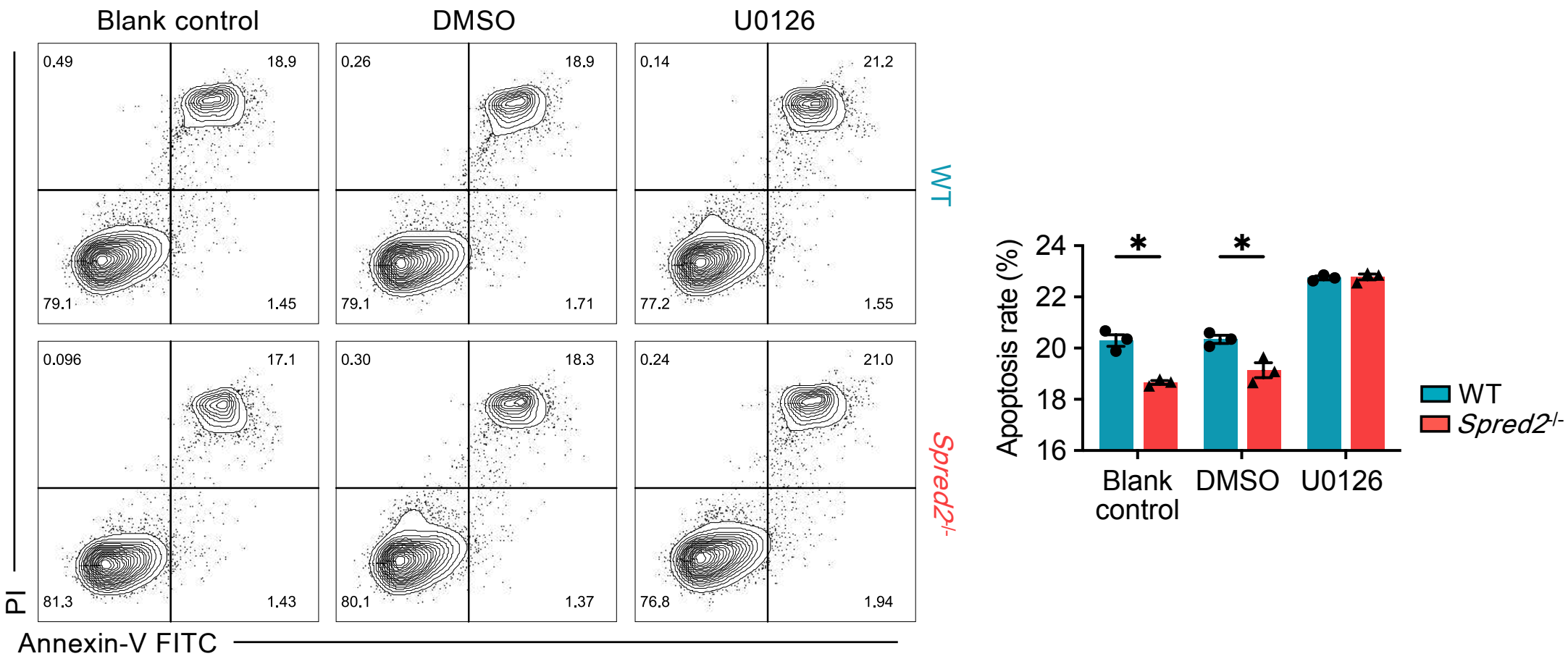

Figure S6. ERK inhibition abolishes the apoptosis difference between WT and *Spred2*$^{-/-}$ CD8^{+} T cells. CD8^{+} T cells from naive WT and *Spred2*$^{-/-}$ mice were left untreated or pre-incubated with 10 μM U0126 or DMSO for 30 minutes, then stimulated with anti-CD3e/CD28 mAbs for 24 hours and analyzed by Annexin V–FITC/PI flow cytometry. Left, representative contour plots showing viable (Annexin V^{-}/PI^{-}), early apoptotic (Annexin V^{+}/PI^{-}), late apoptotic (Annexin V^{+}/PI^{+}), and Annexin V^{-}/PI^{+} populations (representative of n = 3 biological replicates). Right, the apoptosis rate, calculated as the sum of early and late apoptotic cells (n = 3 biological replicates). Data are presented as mean ± SEM. Statistics: two-way ANOVA with Šídák's multiple-comparisons test. *$p < 0.05$.

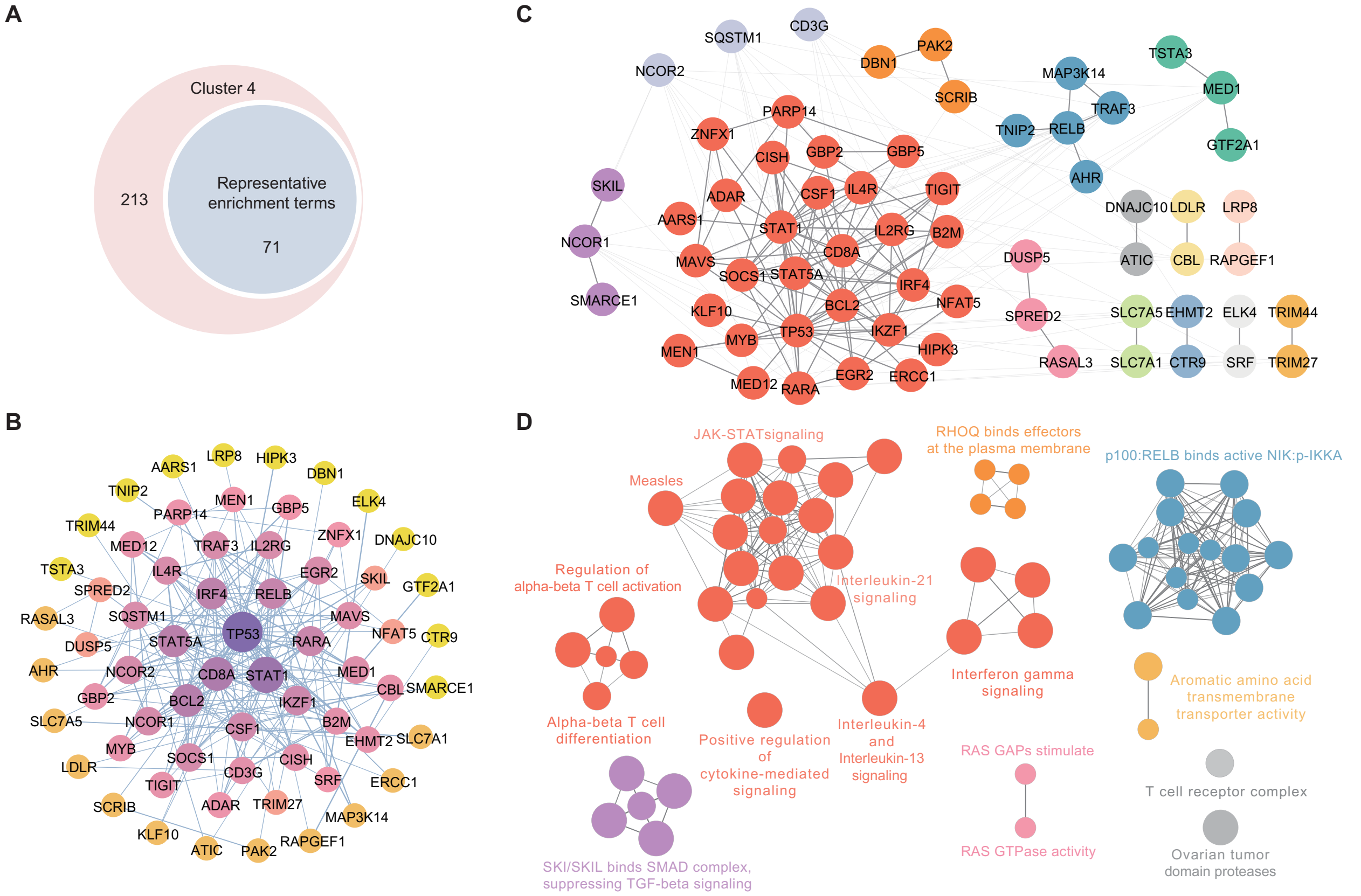

Figure S7. Molecular connectivity and unsupervised clustering of the brake module from time-series data. (A) Enriched terms (GO/KEGG/Reactome) were collected for cluster 4 (Figure 7B), and their deduplicated member genes were combined (n = 71). (B) PPI (protein-protein interaction) network of genes from (A) was assembled in STRING (confidence ≥ 0.4); edges denote interactions (combined score), node size/color encode degree (enrichment $p < 1 \times 10^{-16}$). C, D) Markov clustering (MCL; inflation = 3) was applied on the PPI network: subclusters (C) and enriched terms by subcluster (D).

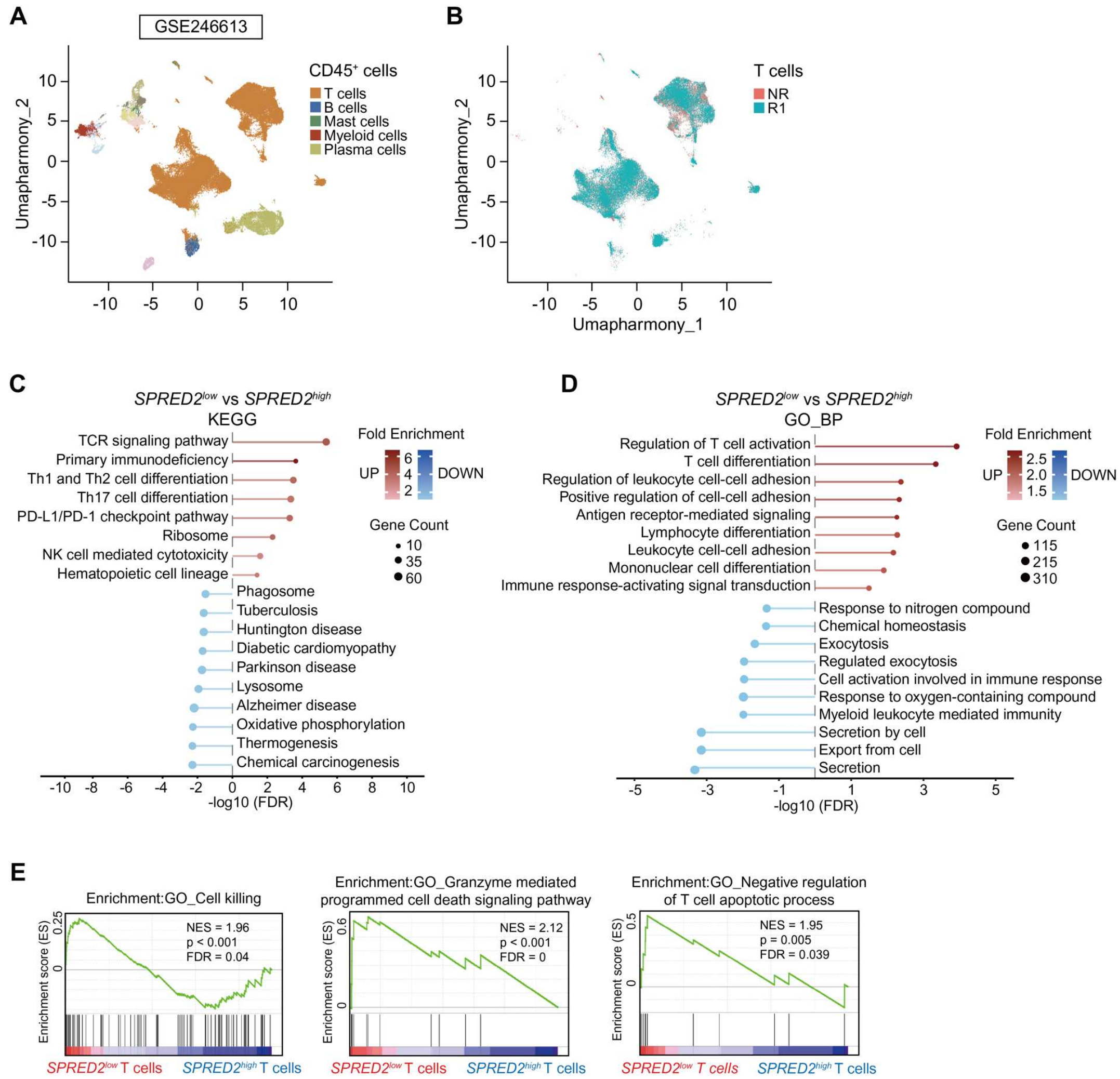

Figure S8. *SPRED2*^{low} T cells are enriched for T-cell activation, cytotoxicity, and survival programs in human TNBC. (A) UMAP of CD45^{+} immune cells from 10 baseline TNBC biopsies pre-pembrolizumab, with cell-type annotations (GSE246613: responders; R1, n = 5, non-responders; NR, n = 5). (B) UMAP of the T cell compartment colored by response group showing the distribution of R1 and NR across T cell clusters (n = 5 per group). C, D) Functional enrichment analysis of differentially expressed genes between *SPRED2*^{low} and *SPRED2*^{high} T-cell populations using KEGG (C) and GO_BP (D). (E) GSEA enrichment profiles for representative pathways in *SPRED2*^{low} vs *SPRED2*^{high} T cells, with exemplar plots.

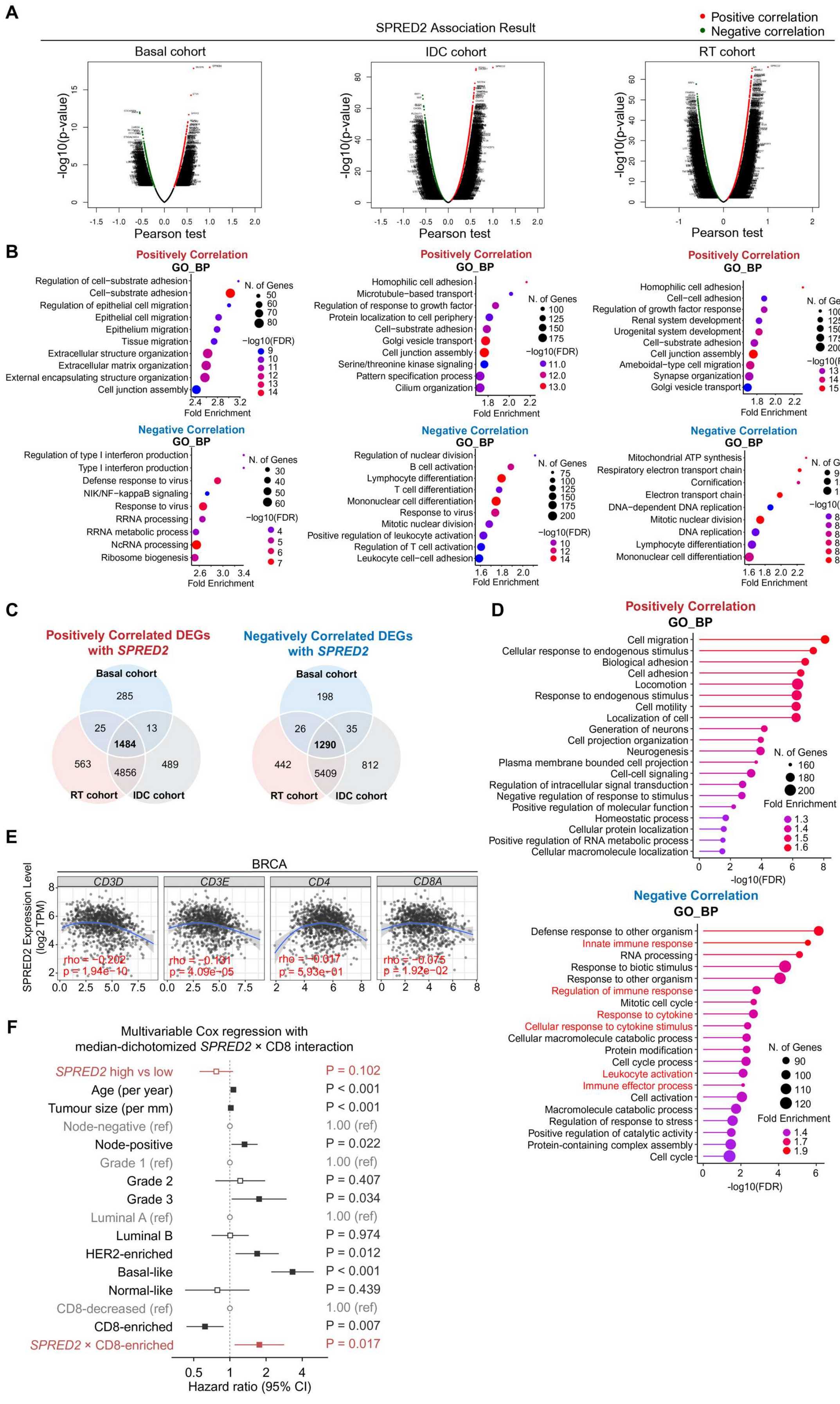

Figure S9. SPRED2 expression is inversely associated with immune T-cell signatures and prognosis in breast cancer cohorts. (A) Volcano plots of SPRED2 co-expression in basal-like, IDC (invasive ductal carcinoma), and radiotherapy (RT) cohort (LinkedOmics); FDR-significant genes highlighted (red, concordant; green, discordant with SPRED2). (B) ORA (over-representation analysis) of DEGs from (A) (top 10). (C) Cross-cohort overlap of genes positively and negatively correlated with SPRED2 across the three cohorts. (D) ORA of the intersection in (C), highlighting immune system process (top 20). (E) Scatter plots depict tumor purity-adjusted partial Spearman correlations (TIMER 3.0, TCGA-BRCA) between *SPRED2* and T cell markers. Purity is included as a covariate (rank-based partial correlation). (F) Multivariable Cox proportional-hazards model for OS with *SPRED2* dichotomized at the cohort median. Squares indicate hazard ratios (HRs) and horizontal lines indicate 95% confidence intervals (CIs).

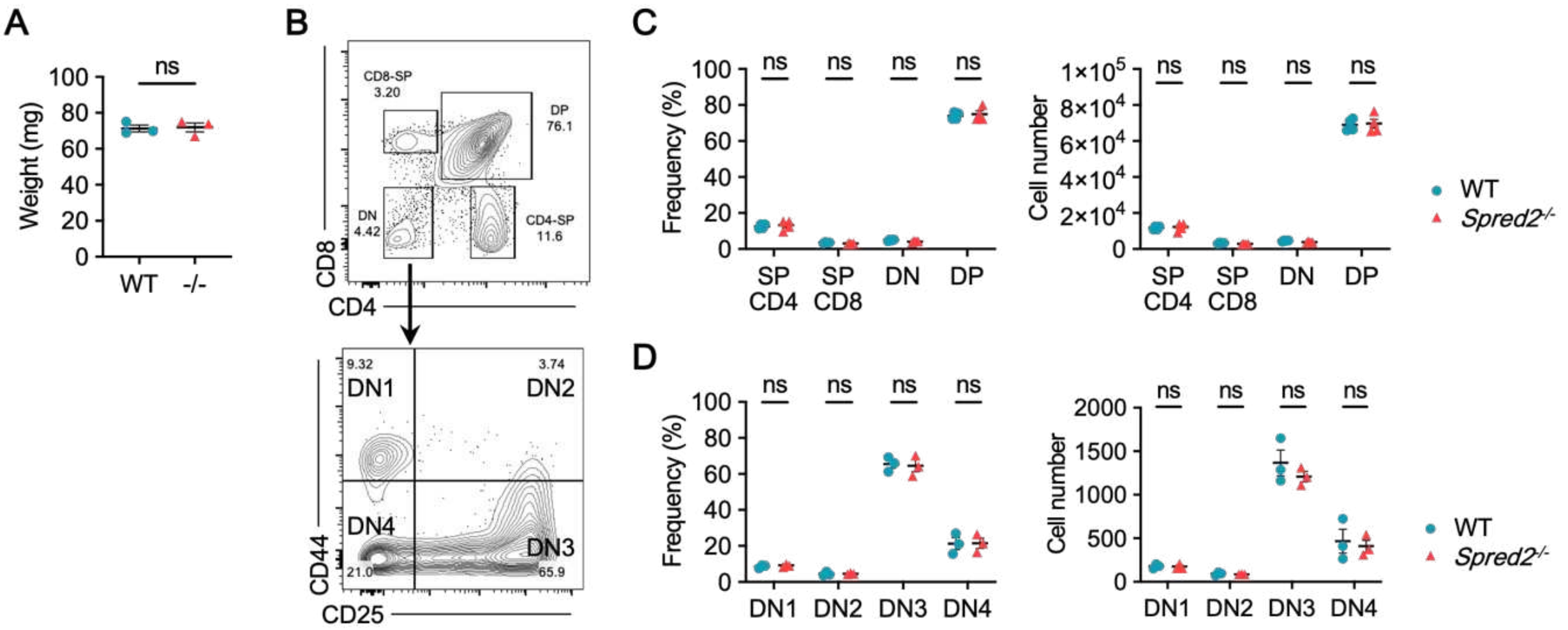

Figure S10. Spred2 deficiency does not alter steady-state thymic T-cell development. Thymi from naive WT and *Spred2*$^{-/-}$ mice were analyzed (n = 3 biological replicates). (A) Thymus weight; (B) Representative gating: Upper panel, CD4-SP ($CD4^+$ single-positive); CD8-SP ($CD8^+$ single-positive); DP ($CD4^+CD8^+$ double-positive) and DN ($CD4^-CD8^-$ double-negative) populations. Lower panel, DN cells were further resolved by CD44 versus CD25 into DN1 ($CD44^+CD25^-$), DN2 ($CD44^+CD25^+$), DN3 ($CD44^-CD25^+$) and DN4 ($CD44^-CD25^-$). (C) Frequencies (left) and numbers (right) of CD4-SP, CD8-SP, DN, and DP thymocytes. (D) Frequencies and numbers of DN1–DN4 subsets. Data are presented as mean ± SEM. Statistics: (A) unpaired t-test; (C, D) repeated-measures two-way ANOVA with Šídák's multiple-comparisons test. ns, not significant.

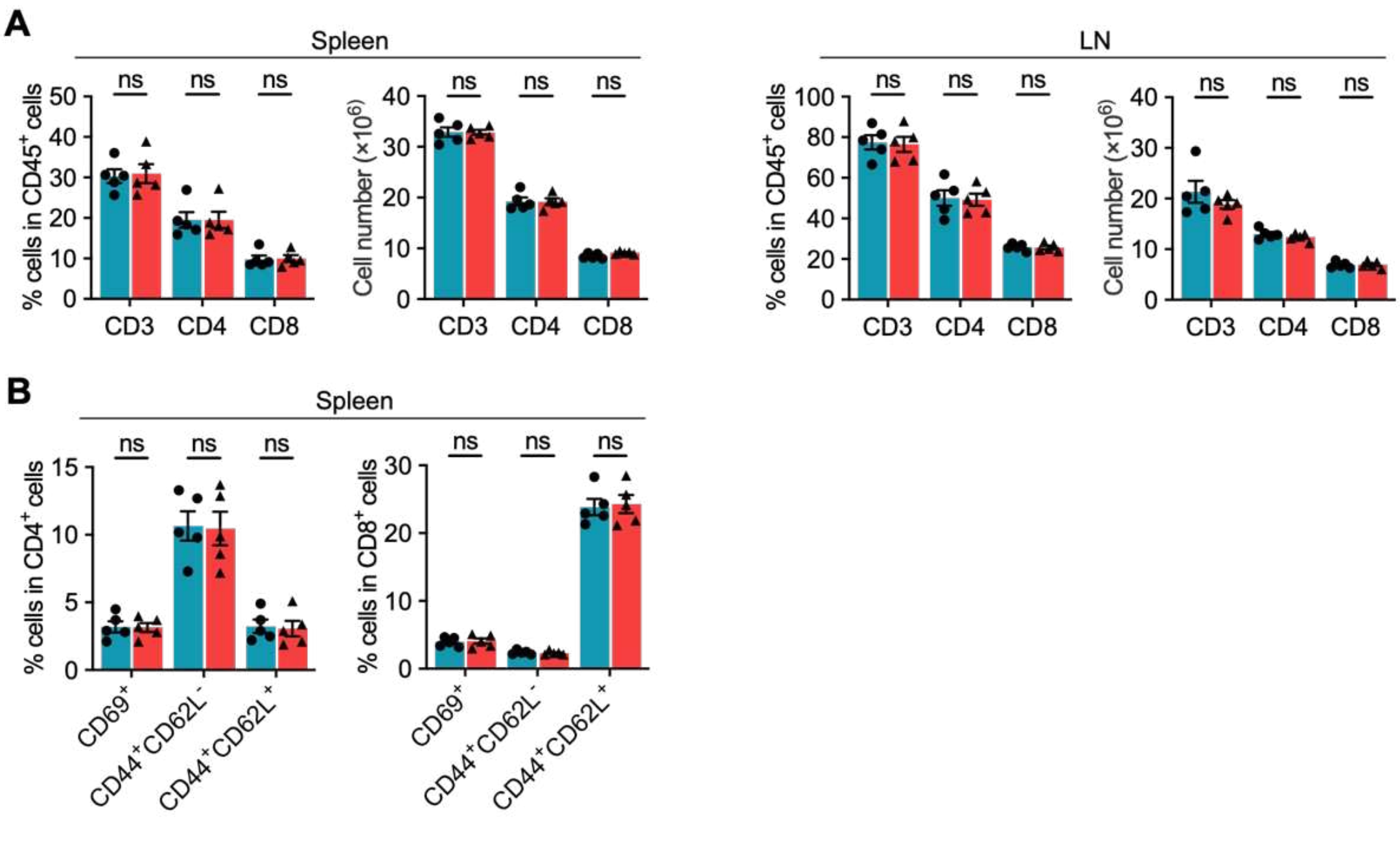

Figure S11. Spred2 deficiency does not alter peripheral T-cell homeostasis in tumor-free mice. Spleen and peripheral lymph nodes (LN) from naive WT and *Spred2*$^{-/-}$ mice were analyzed (n = 5 per group). (A) Frequencies and numbers of $CD3^+$, $CD4^+$, and $CD8^+$ T cells in spleen (left) and LN (right). (B) Frequencies of $CD69^+$, effector-memory ($CD44^+CD62L^-$), and central-memory ($CD44^+CD62L^+$) cells among splenic $CD4^+$ and $CD8^+$ T cells. Data are presented as mean ± SEM. Statistics: two-way repeated-measures ANOVA (mixed-effects model) with Šídák's multiple-comparisons test. ns, not significant.

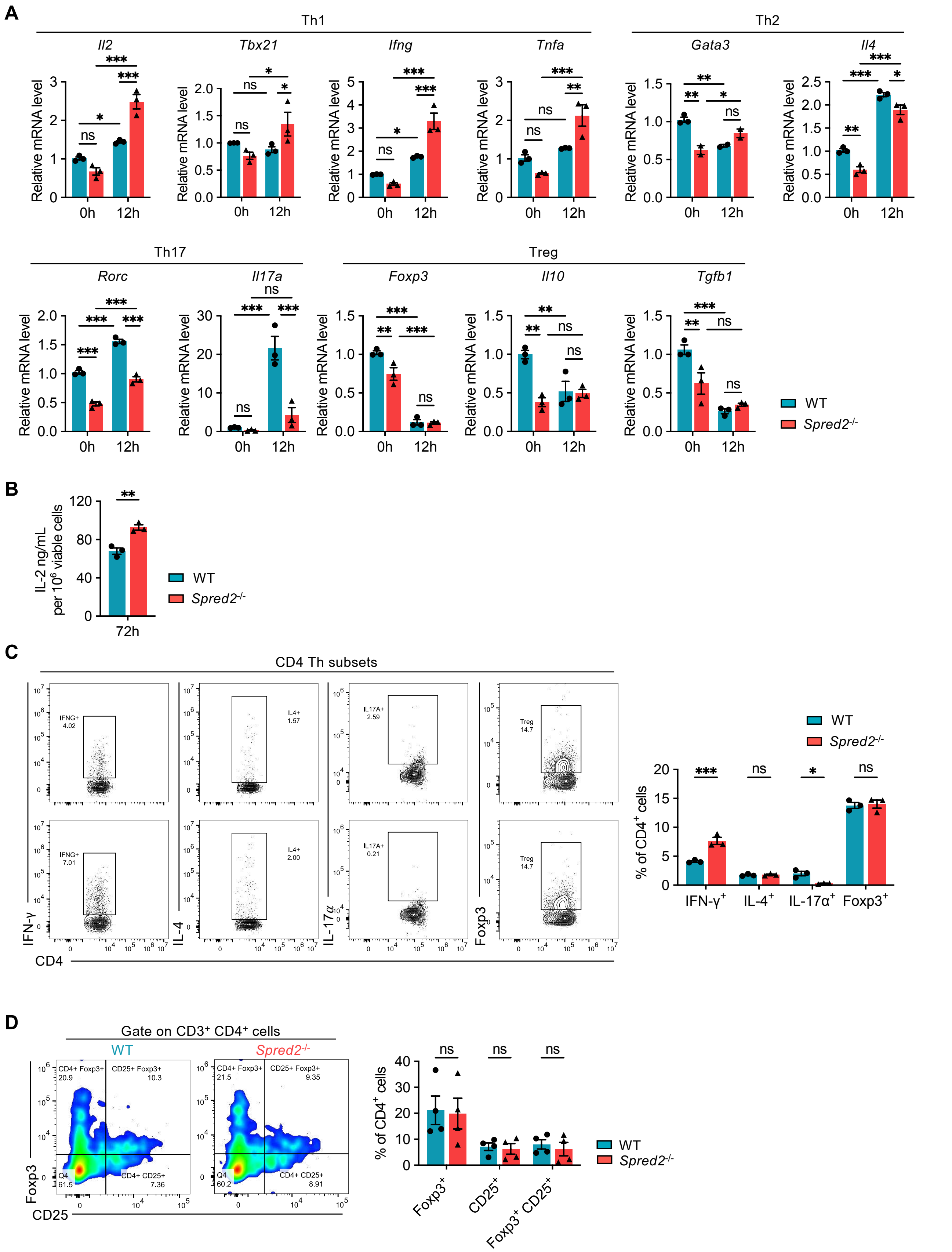

Figure S12. Spred2 deficiency affects $CD4^+$ T-cell responses. A, B) $CD4^+$ T cells were isolated from naive WT and *Spred2*$^{-/-}$ mice and stimulated with anti-CD3e/CD28 mAbs. (A) RT-qPCR of Th1 (Il2, Tbx21, Ifng, Tnfa), Th2 (Gata3, Il4), Th17 (Rorc, Il17a), and Treg (Foxp3, Il10, Tgfb1) genes before and after 12 hours stimulation; (n = 3 biological replicates); (B) IL-2 level in 72-hour supernatants by ELISA (n = 3 biological replicates). (C) Naive $CD4^+$ T cells from WT or *Spred2*$^{-/-}$ mice were activated with PMA/ionomycin + brefeldin A (BFA) (6 h). Representative flow cytometry plots (left) and quantification of intracellular IFN-γ^+, IL-4^+, IL-$17A^+$, and $Foxp3^+$ cells (n = 3 biological replicates). (D) 4T1 tumors were resected 28 days after implantation of 4T1 cells (5×10^5 cells/mouse) into WT and *Spred2*$^{-/-}$ BALB/c mice. Representative flow cytometry plots (left) and quantification of $Foxp3^+$, $CD25^+$, and $Foxp3^+CD25^+$ cells among CD4+ T cells are shown (n = 5 biological replicates). Data are presented as mean ± SEM. Statistics: (A) two-way ANOVA with Šídák's multiple-comparisons test, (B) unpaired Student t-test, (C) multiple unpaired t-tests with Šídák's correction, (D) two-way repeated-measures ANOVA with Šídák's multiple-comparisons test. *$p < 0.05$, **$p < 0.01$, ***$p < 0.001$; ns, not significant.

A Gating of tumor-infiltrating immune subsets in 4T1 tumors

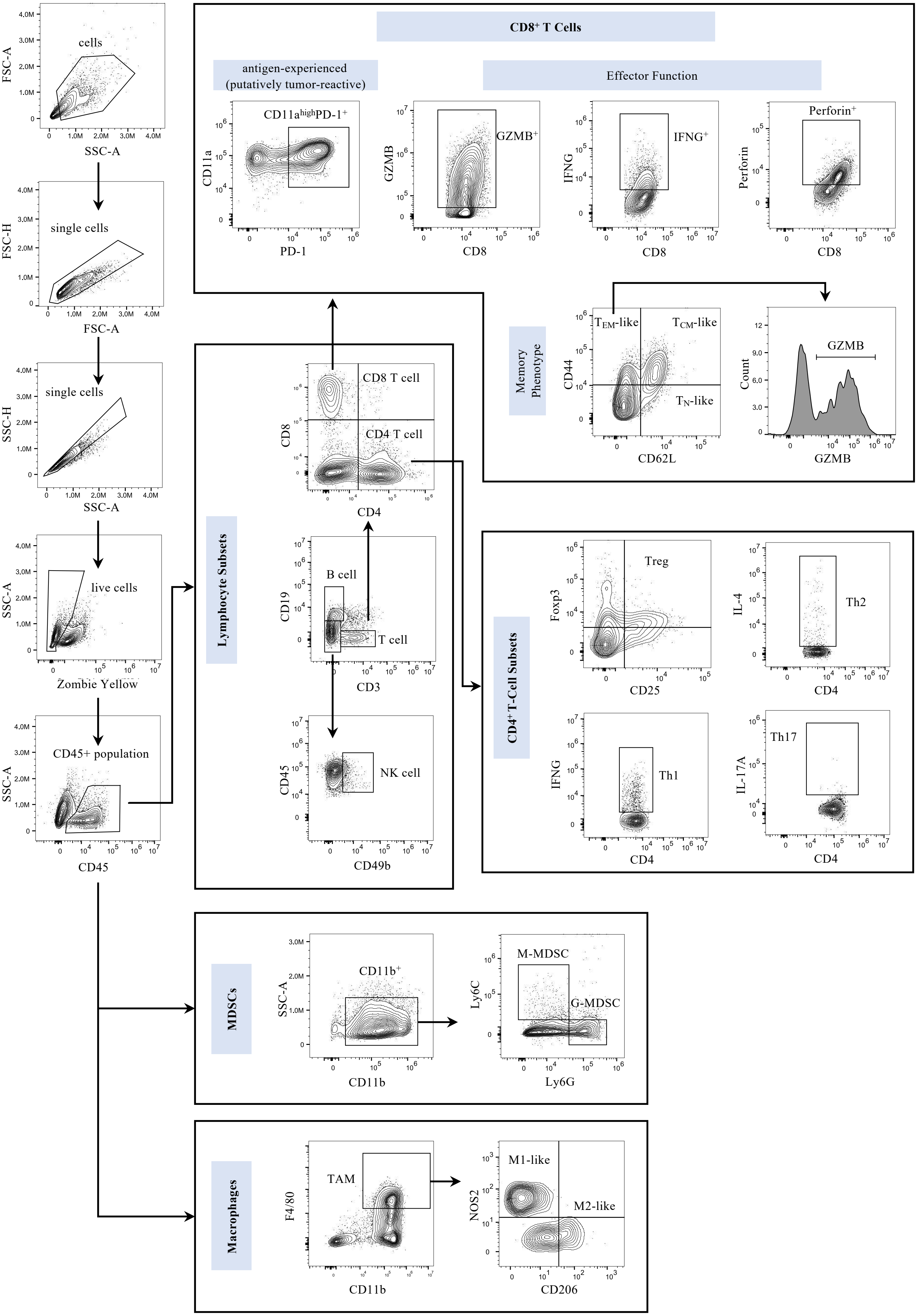

B Gating of lung CD8$^+$ T_{RM}-like cells in tumor-bearing mice

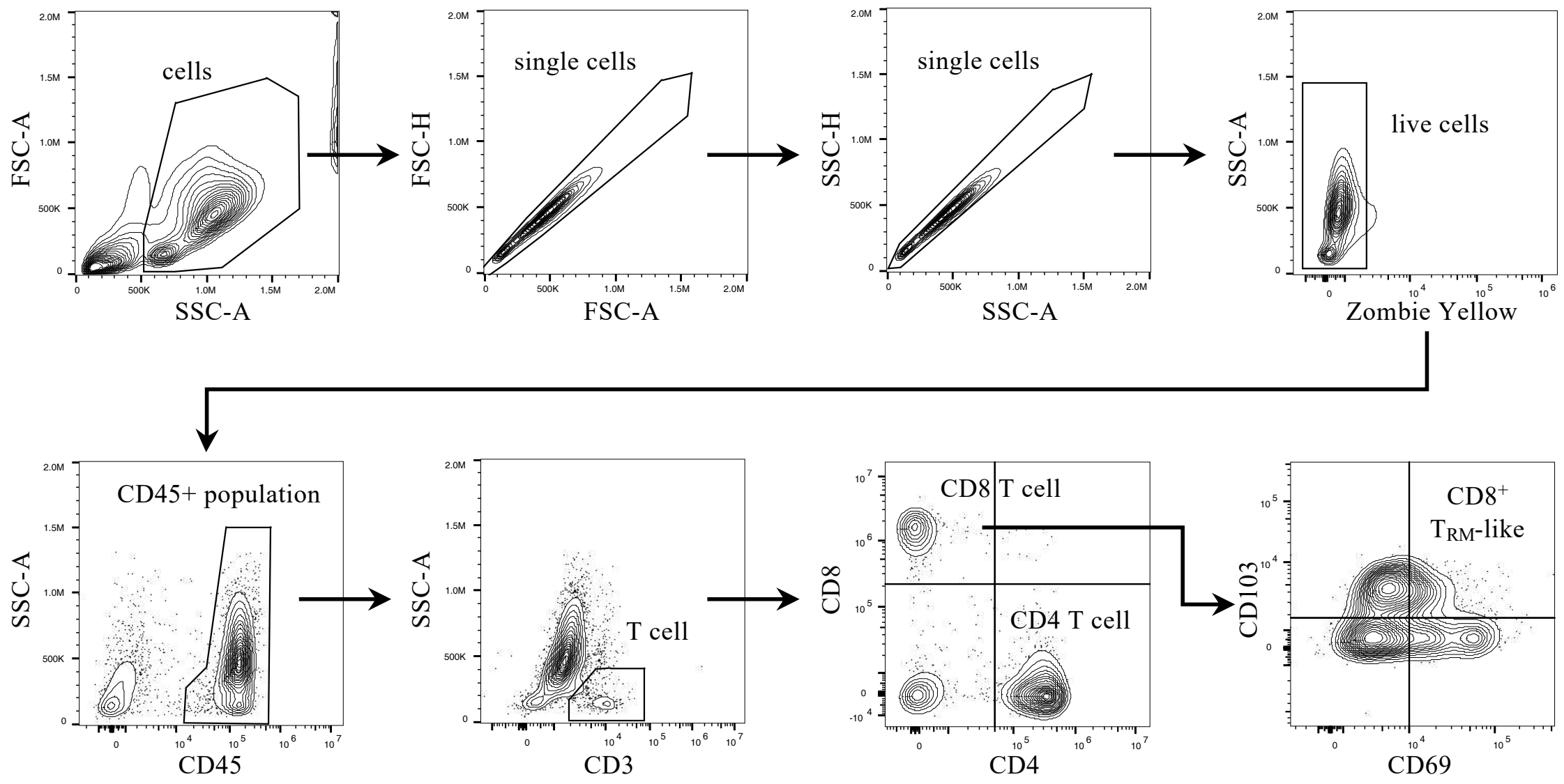

Figure S13. Representative flow cytometry gating strategies. A) Representative gating strategy for tumor-infiltrating immune cells isolated from 4T1 tumors. Cells were sequentially gated based on FSC-A/SSC-A, singlets (FSC-A/FSC-H and SSC-A/SSC-H), live cells (Zombie Yellow$^-$), and CD45$^+$ leukocytes. CD45$^+$ cells were further analyzed to identify lymphocyte subsets, including CD3$^+$ T cells, CD19$^+$ B cells, and CD49b$^+$ NK cells; CD8$^+$ and CD4$^+$ T-cell subsets; myeloid-derived suppressor cells (MDSCs); and tumor-associated macrophages (TAMs). CD8$^+$ T cells were further assessed for CD11a^{high}PD-1$^+$ antigen-experienced (putatively tumor-reactive) cells, effector molecules (GZMB, IFN-γ, and perforin), and T_N-like, T_{CM}-like, and T_{EM}-like phenotypes based on CD44 and CD62L expression. CD4$^+$ T-cell subsets included Treg, Th1, Th2, and Th17 cells. MDSCs were classified into M-MDSCs and G-MDSCs based on Ly6C and Ly6G expression, and TAMs were further evaluated for M1- and M2-like phenotypes based on NOS2$^+$ and CD206 expression. B) Representative gating strategy for lung CD8$^+$ T_{RM}-like cells from tumor-bearing mice. Following sequential gating of cells, singlets, live cells, CD45$^+$ leukocytes, and CD3$^+$ T cells, CD8$^+$ T cells were identified, and CD69$^+$CD103$^+$ cells were defined as CD8$^+$ T_{RM}-like cells. T_N-like, naive-like T cells; T_{CM}-like, central memory-like T cells; TEM-like, effector memory-like T cells; TRM, tissue-resident memory-like T cells.
