# Supplementary material for: SPRED2 Negatively Regulates CD8+ T Cell‐Mediated Antitumor Immunity in Breast Cancer
Source: Adv Sci (Weinh). 2026 Sep 27:e77909. Online ahead of print. doi: 10.1002/advs.77909 (PMC13616240; doi:10.1002/advs.77909)
Supplement: Supplementary file 2 [file ADVS-9999-e77909-s001.docx]

**Table S1. Clinical characteristics of patients used for analysis**

| **Paper Number** | **Histology** | **RCB** | **RCB Class** | **Age** | **Stage** | **TIL** |
| --- | --- | --- | --- | --- | --- | --- |
| 2 | invasive ductal | 1.244 | 1 | 53 | IIB | 0.05 |
| 4 | invasive ductal |  | 0 | 67 | IIA | 0.10 |
| 5 | invasive ductal |  | 0 | 53 | IIB | 0.60 |
| 9 | invasive ductal |  | 0 | 68 | IIA | 0.50 |
| 10 | invasive ductal | 1.78 | 2 | 36 | IIA | 0.00 |
| 12 | invasive ductal |  | 0 | 43 | IIA | 0.90 |
| 17 | invasive ductal | 3.374 | 3 | 43 | IIA |  |
| 26 | invasive ductal | 1.4 | 2 | 55 | IIB |  |
| 45 | invasive ductal |  | 2 | 94 | IIA |  |
| 53 | invasive ductal |  | 3 | 48 | IIB |  |

RCB: Residual Cancer Burden

RCB Class 1 and 0 are considered pathologic complete response

TIL: Tumor Infiltrating Leukocyte

**Table S2.** **The gene list for the *SPRED2^high^* T-cell signature, related to Figure 8**

***SPRED2^high^* T-cell signature genes**

*MYO5A*

*DLEU1*

*DNMT1*

*CAP1*

*PSMD14*

*DNAJC7*

*SNX6*

*TPM3*

*ANXA7*

*SPPL2A*

*CALM3*

*SPRED2*

**Table S3. Patient numbers and death events by subgroup**

| **Subgroup** | **n** | **Events** | **Event rate** | **Complete**  **cases, n** | **Complete**  **cases, events** | **Events excluded** |
| --- | --- | --- | --- | --- | --- | --- |
| Whole cohort | 2976 | 311 | 10.5% | 2825 | 292 | 19 |
| PAM50 luminal A | 1502 | 107 | 7.1% | 1443 | 102 | 5 |
| PAM50 luminal B | 669 | 75 | 11.2% | 640 | 69 | 6 |
| PAM50 HER2-enriched | 297 | 50 | 16.8% | 265 | 45 | 5 |
| PAM50 basal-like | 308 | 65 | 21.1% | 286 | 64 | 1 |
| PAM50 normal-like | 200 | 14 | 7.0% | 191 | 12 | 2 |
| CD8-enriched | 1488 | 137 | 9.2% | 1396 | 130 | 7 |
| CD8-decreased | 1488 | 174 | 11.7% | 1429 | 162 | 12 |
| Basal-like and CD8-enriched | 219 | 36 | 16.4% | 201 | 35 | 1 |
| Basal-like and CD8-decreased | 89 | 29 | 32.6% | 85 | 29 | 0 |
| Node-negative | 1820 | 158 | 8.7% | 1791 | 157 | 1 |
| Node-positive | 1067 | 136 | 12.7% | 1034 | 135 | 1 |
| Nottingham grade 1 | 449 | 23 | 5.1% | 437 | 22 | 1 |
| Nottingham grade 2 | 1394 | 118 | 8.5% | 1357 | 111 | 7 |
| Nottingham grade 3 | 1074 | 169 | 15.7% | 1031 | 159 | 10 |

Complete cases refer to patients with no missing value among the covariates used in the multivariable models; events excluded is the difference in deaths between the full and complete-case subgroup.

**Table S4.** **Kaplan–Meier and univariable Cox analyses of *SPRED2* expression and the *SPRED2^high^* T cell signature in breast cancer cohorts.**

| **Figure** | **Stratifying**  **variable** | **Cohort** | **Cutoff** | **Log-rank**  **P** | **Univariable**  **HR** | **95% CI** | **Adjusted P**  **(BH)** |
| --- | --- | --- | --- | --- | --- | --- | --- |
| 8G | *SPRED2* | BRCA | median | 0.5811 | 1.06 | 0.85 – 1.33 | 0.638 |
| 8J | *SPRED2* | Basal-like | median | 0.0411 | 1.66 | 1.09 – 2.73 | 0.082 |
| 8K | *SPRED2* | CD8-enriched | median | 0.0228 | 1.48 | 1.05 – 2.09 | 0.082 |
| 8K | *SPRED2* | CD8-decreased | median | 0.0771 | 0.76 | 0.57 – 1.03 | 0.116 |
| 8L | Signature | CD8-decreased | median | 0.6382 | 0.93 | 0.69 – 1.25 | 0.638 |
| 8L | Signature | CD8-enriched | median | 0.0276 | 1.46 | 1.04 – 2.05 | 0.082 |

Cut-offs were the median of the marker within each analyzed cohort; automatic selection of an optimal cut-off was not used. P values are from the log-rank test. Adjusted P values were obtained by the Benjamini–Hochberg procedure across the family of all six comparisons shown (m = 6).

**Table S5. Patient numbers and death events in each arm of the Kaplan-Meier analyses**

| **Figure** | **Cohort** | **Marker** | **n, low** | **Events, low** | **n, high** | **Events, high** | **Total events** |
| --- | --- | --- | --- | --- | --- | --- | --- |
| 8J | Basal-like | *SPRED2* | 154 | 25 | 154 | 40 | 65 |
| 8K | CD8-enriched | *SPRED2* | 744 | 55 | 744 | 82 | 137 |
| 8L | CD8-enriched | 12-gene signature | 744 | 57 | 744 | 80 | 137 |
| 8K | CD8-decreased | *SPRED2* | 743 | 98 | 745 | 76 | 174 |
| 8L | CD8-decreased | 12-gene signature | 744 | 92 | 744 | 82 | 174 |

Low and high arms were defined by the median of the marker within each cohort; ties were assigned to the high arm.

**Table S6. Multivariable Cox proportional-hazards models of *SPRED2* expression for overall survival in the breast cancer cohort**

**(a) *SPRED2*, continuous (per 1 SD)**

| **Group** | **Variable** | **HR** | **95% CI** | **P** |
| --- | --- | --- | --- | --- |
| *SPRED2* | *SPRED2* (per 1 SD) | 1.021 | 0.915-1.140 | 0.709 |
| Clinical | Age (per year) | 1.069 | 1.058-1.081 | <0.001 |
| Clinical | Tumor size (per mm) | 1.017 | 1.011-1.024 | <0.001 |
| Node status | Node-negative | 1.00 (reference) |  |  |
| Node status | Node-positive | 1.348 | 1.059-1.717 | 0.015 |
| Grade | Grade 1 | 1.00 (reference) |  |  |
| Grade | Grade 2 | 1.231 | 0.771-1.965 | 0.385 |
| Grade | Grade 3 | 1.745 | 1.040-2.928 | 0.035 |
| PAM50 | Luminal A | 1.00 (reference) |  |  |
| PAM50 | Luminal B | 1.026 | 0.727-1.448 | 0.885 |
| PAM50 | HER2-enriched | 1.680 | 1.119-2.522 | 0.012 |
| PAM50 | Basal-like | 3.302 | 2.219-4.914 | <0.001 |
| PAM50 | Normal-like | 0.789 | 0.429-1.451 | 0.446 |
| CD8 status | CD8-decreased | 1.00 (reference) |  |  |
| CD8 status | CD8-enriched | 0.842 | 0.657-1.079 | 0.174 |

n = 2825; events = 292; C-index = 0.785

**(b) *SPRED2*, median-dichotomized**

| **Group** | **Variable** | **HR** | **95% CI** | **P** |
| --- | --- | --- | --- | --- |
| *SPRED2* | *SPRED2* high vs low | 0.992 | 0.784-1.257 | 0.950 |
| Clinical | Age (per year) | 1.070 | 1.058-1.081 | <0.001 |
| Clinical | Tumor size (per mm) | 1.017 | 1.011-1.024 | <0.001 |
| Node status | Node-negative | 1.00 (reference) |  |  |
| Node status | Node-positive | 1.348 | 1.059-1.717 | 0.015 |
| Grade | Grade 1 | 1.00 (reference) |  |  |
| Grade | Grade 2 | 1.228 | 0.769-1.960 | 0.390 |
| Grade | Grade 3 | 1.734 | 1.034-2.907 | 0.037 |
| PAM50 | Luminal A | 1.00 (reference) |  |  |
| PAM50 | Luminal B | 1.021 | 0.723-1.443 | 0.906 |
| PAM50 | HER2-enriched | 1.681 | 1.120-2.523 | 0.012 |
| PAM50 | Basal-like | 3.295 | 2.214-4.906 | <0.001 |
| PAM50 | Normal-like | 0.786 | 0.428-1.446 | 0.439 |
| CD8 status | CD8-decreased | 1.00 (reference) |  |  |
| CD8 status | CD8-enriched | 0.834 | 0.652-1.068 | 0.150 |

n = 2825; events = 292; C-index = 0.785

In the no-interaction models the SPRED2 coefficient is the cohort-wide effect, averaged over CD8 strata under the assumption that the effect is common to both.

HR, hazard ratio; CI, confidence interval. All P values are from the Wald test.

**Table S7. Multivariable Cox proportional-hazards models including the *SPRED2* × CD8 interaction for overall survival in the breast cancer cohort**

**(a) *SPRED2*, continuous (per 1 SD)**

| **Group** | **Variable** | **HR** | **95% CI** | **P** |
| --- | --- | --- | --- | --- |
| *SPRED2* | *SPRED2* (per 1 SD) | 0.934 | 0.810-1.077 | 0.347 |
| Clinical | Age (per year) | 1.069 | 1.058-1.080 | <0.001 |
| Clinical | Tumor size (per mm) | 1.018 | 1.011-1.024 | <0.001 |
| Node status | Node-negative | 1.00 (reference) |  |  |
| Node status | Node-positive | 1.336 | 1.049-1.701 | 0.019 |
| Grade | Grade 1 | 1.00 (reference) |  |  |
| Grade | Grade 2 | 1.242 | 0.778-1.984 | 0.363 |
| Grade | Grade 3 | 1.774 | 1.058-2.976 | 0.030 |
| PAM50 | Luminal A | 1.00 (reference) |  |  |
| PAM50 | Luminal B | 1.018 | 0.721-1.438 | 0.919 |
| PAM50 | HER2-enriched | 1.684 | 1.122-2.527 | 0.012 |
| PAM50 | Basal-like | 3.406 | 2.287-5.073 | <0.001 |
| PAM50 | Normal-like | 0.797 | 0.433-1.464 | 0.464 |
| CD8 status | CD8-decreased | 1.00 (reference) |  |  |
| CD8 status | CD8-enriched | 0.834 | 0.653-1.065 | 0.147 |
| Interaction | *SPRED2* **×** CD8-enriched | 1.254 | 1.010-1.556 | 0.040 |
| Derived | *SPRED2* effect, CD8-decreased stratum | 0.934 | 0.810–1.077 | 0.347 |
| Derived | *SPRED2* effect, CD8-enriched stratum | 1.171 | 0.982–1.396 | 0.078 |

n = 2825; events = 292; C-index = 0.787; interaction Wald P = 0.050, likelihood-ratio P = 0.050

**(b) *SPRED2*, median dichotomy**

| **Group** | **Variable** | **HR** | **95% CI** | **P** |
| --- | --- | --- | --- | --- |
| *SPRED2* | *SPRED2* high vs low | 0.771 | 0.564-1.053 | 0.102 |
| Clinical | Age (per year) | 1.069 | 1.058-1.080 | <0.001 |
| Clinical | Tumor size (per mm) | 1.018 | 1.011-1.024 | <0.001 |
| Node status | Node-negative | 1.00 (reference) |  |  |
| Node status | Node-positive | 1.325 | 1.041-1.686 | 0.022 |
| Grade | Grade 1 | 1.00 (reference) |  |  |
| Grade | Grade 2 | 1.219 | 0.763-1.947 | 0.407 |
| Grade | Grade 3 | 1.750 | 1.043-2.936 | 0.034 |
| PAM50 | Luminal A | 1.00 (reference) |  |  |
| PAM50 | Luminal B | 1.006 | 0.711-1.422 | 0.974 |
| PAM50 | HER2-enriched | 1.686 | 1.124-2.530 | 0.012 |
| PAM50 | Basal-like | 3.321 | 2.231-4.944 | <0.001 |
| PAM50 | Normal-like | 0.787 | 0.428-1.446 | 0.439 |
| CD8 status | CD8-decreased | 1.00 (reference) |  |  |
| CD8 status | CD8-enriched | 0.621 | 0.439-0.878 | 0.007 |
| Interaction | *SPRED2* **×** CD8-enriched | 1.759 | 1.104-2.802 | 0.017 |
| Derived | *SPRED2* effect, CD8-decreased stratum | 0.771 | 0.564-1.053 | 0.102 |
| Derived | *SPRED2* effect, CD8-enriched stratum | 1.356 | 0.958-1.919 | 0.086 |

n = 2825; events = 292; C-index = 0.786; interaction Wald P = 0.017, likelihood-ratio P = 0.018

In the interaction models the SPRED2 coefficient is instead the SPRED2 effect within CD8-decreased patients (the CD8 reference level) and must not be read as a cohort-wide effect; likewise the CD8 coefficient becomes the CD8 effect at SPRED2 = 0 (binary models) or at the cohort mean (continuous models) rather than a marginal effect.

HR, hazard ratio; CI, confidence interval. All P values are from the Wald test.

**Table S8.** **Antibodies for immunohistochemistry**

| **Antibody** | **Dilution** | **Company (Cat. Number)** |
| --- | --- | --- |
| **Primary antibody**  CD3  CD4  CD8  **Secondary antibody**  Goat anti-rabbit IgG HRP  Polink-2 Plus HRP anti Mouse Detection Kit | undiluted  1:4000  1:500  undiluted  undiluted | Nichirei Bioscience Inc (413591)  Abcam (ab237722)  Cell Signaling Technology (98941T)  Nichirei Bioscience Inc (414341)  GBI Labs (D37-18) |

Abcam: Cambridge, UK

Cell Signaling Technology: Danvers, MA, USA

GBI Labs: Rockville, MD, USA

Nichirei Bioscience Inc: Tokyo, Japan

**Table S9.** **Taqman gene expression assays used for RT-qPCR**

| **Gene name** | **Taqman gene expression assay kit** |
| --- | --- |
| *Ifng*  *Tnfa*  *Granzyme B (Gzmb)*  *Perforin-1 (Prf1)*  *Cxcl9*  *Cxcl10*  *I12a (p35)*  *Il12b (p40)*  *Cxcr3*  *Tbx21*  *Gata3*  *Lef1*  *Il2*  *Fas*  *FasL*  *Spred2*  *Gapdh* | Mm01168134_m1  Mm00443258_m1  Mm00442834_m1  Mm00812512_m1  Mm00434946_m1  Mm00445275_m1  Mm00434165_m1  Mm00434174_m1  Mm99999054_sl  Mm00450960_m1  Mm00484683_m1  Mm00550265_m1  Mm00439861_m1  Mm01204974_m1  Mm00438864_m1  Mm01223872_g1  Mm99999915_g1 |

**Table S10**. **Antibodies for flow cytometer**

| **Antigen** | **Company (Cat. number)** | **clone name** |
| --- | --- | --- |
| CD16/32 | BioLegend (101302) | 93 |
| CD3-APC/Cy7 | BioLegend (100221) | 17A2 |
| CD3-Pacific Blue | BioLegend (100213) | 17A2 |
| CD4-APC | BioLegend (100411) | GK1.5 |
| CD4-FITC | BioLegend (130308) | H129.19 |
| CD8α-PE | BioLegend (100708) | 53-6.7 |
| CD8α-PerCP/Cy5.5 | BioLegend (100734) | 53-6.7 |
| CD103-PerCP/Cy5.5 | BioLegend (121415) | 2E7 |
| CD69-PE/Cy7 | BioLegend (104511) | H1.2F3 |
| CD62L-FITC | BioLegend (161212) | W18021D |
| CD44-PE/Cy7 | BioLegend (103029) | IM7 |
| CD45-APC/Cy7 | BioLegend (103115) | 30-F11 |
| CD45-FITC | BioLegend (157214) | S18009F |
| GZMB-PE | BioLegend (372207) | QA16A02 |
| IFNG-FITC | BioLegend (505806) | XMG1.2 |
| IFNG-APC | BioLegend (505810) | XMG1.2 |
| CD11a-APC | BioLegend (153110) | I21/7 |
| PD-1-BV421 | BioLegend (135217) | 29F.1A12 |
| Perforin-PE | BioLegend (154305) | S16009A |
| CD25-FITC | BioLegend (101908) | 3C7 |
| p-ERK-PerCP/Cy5.5 | BioLegend (369511) | 6B8B69 |
| CD11b-APC | BioLegend (101212) | M1/70 |
| CD11b-FITC | BioLegend (101206) | M1/70 |
| F4/80-PE | BioLegend (111604) | W20065B |
| Ly6G/Ly6C-FITC | BioLegend (108406) | RB6-8C5 |
| CD19-FITC | BioLegend (152403) | 1D3/CD19 |
| CD49b-PerCP/Cy5.5 | BioLegend (103519) | HMα2 |
| IL-4-BV605 | BioLegend (504125) | 11B11 |
| IL-17A-PerCP/Cy5.5 | BioLegend (506919) | TC11-18H10.1 |
| Foxp3-PE | BioLegend (126403) | MF-14 |
| Ly6G-FITC | BioLegend (127606) | 1A8 |
| Ly6C-PerCP/Cy5.5 | BioLegend (128012) | HK1.4 |
| NOS2-PE-Cy7 | BioLegend (696813) | W16030C |
| CD206-APC | BioLegend (141708) | C068C2 |

BioLegend: San Diego, CA, USA

**Table S11.** Antibodies for Western blotting

| **Antibody** | **Dilution** | **Company (Cat. Number)** |
| --- | --- | --- |
| **Primary antibody**  GAPDH  Bcl-2  p44/42 MAPK (ERK1/2)  Phospho-p44/42 MAPK (pERK1/2)(Thr 202/Tyr 204)  **Secondary antibody**  HRP-goat anti-rabbit IgG  HRP-anti-mouse IgG | 1:5000  1:1000  1:1000  1:1000  1:2000  1:2000 | Cell Signaling Technology (5174)  Cell Signaling Technology (3498)  Cell Signaling Technology (4695)  Cell Signaling Technology (4370)  Cell Signaling Technology (7074)  Cell Signaling Technology (7076) |

Cell Signaling Technology: Danvers, MA, USA
