# Supplementary material for: SPRED2 Negatively Regulates CD8+ T Cell‐Mediated Antitumor Immunity in Breast Cancer
Source: Adv Sci (Weinh). 2026 Sep 27:e77909. Online ahead of print. doi: 10.1002/advs.77909 (PMC13616240; doi:10.1002/advs.77909)
Supplement: Supplementary file 3 [file ADVS-9999-e77909-s003.docx]

**Supplementary Methods**

**Cell culture, immune-cell isolation, and functional assays**

*Cell lines:* The murine BC cell lines, 4T1 and EO771, were obtained from American Type Culture Collection (ATCC, USA). 4T1 cells were grown in RPMI 1640 medium (Sigma-Aldrich, USA), supplemented with 10% fetal bovine serum (FBS) (Gibco, Carlsbad, CA, USA), 2 mM L-glutamine, 1 mM sodium pyruvate (Wako, Japan), 100 U/mL penicillin and 100 μg/mL streptomycin (Sigma-Aldrich, St. Louis, MO) (complete medium). EO771 cells were grown in Dulbecco’s modified Eagle medium (DMEM) (Nacalai Tesque, Kyoto, Japan), supplemented with 10% FBS, 100 U/mL penicillin and 100 μg/mL streptomycin (complete medium). All experiments were performed using mycoplasma-free cells.

*Cell isolation:* For immune cell isolation from spleens and TDLNs (axillary and cervical), tissues were homogenized with RPMI-1640 complete medium, and red blood cells were lysed with ACK lysis buffer (0.15 M NH_4_Cl, 10 mM KHCO_3_, 0.5 mM EDTA). After filtering through 70 µm nylon mesh, the cells were washed with PBS. Splenocytes were dispersed in cell suspensions. Pan T cells or CD8^+^ T cells were isolated from splenocyte suspensions using a Pan T or CD8a^+^ T cell isolation kit (Miltenyi Biotec, Bergisch Gladbach, Germany), respectively. Purity and percent of live cells were > 95%. For single cell isolation from tumors and lungs, tissues were excised, minced, and digested with 5 mL digestion buffer (RPMI-1640 containing 20 mM HEPES, pH 7.4, plus 175U/mL type V collagenase, 0.5mg/mL DNase I) for 30 minutes using gentleMACS^TM^ dissociator (Miltenyi Biotec). After filtering through 70 µm nylon mesh, the cells were washed and red blood cells were lysed with ACK lysis buffer.

*Analysis of cell proliferation:* T cell suspensions (1 × 10^7^ cells in 1 mL PBS) were incubated with CFSE or CTV at a final concentration of 5 μM for 20 minutes at 37°C. After incubation, 5 volumes of RPMI-1640 containing 10% FBS were added, and the mixture was incubated for another 5 minutes at room temperature. After two washes, the CFSE-labeled T cells were adjusted to a concentration of 1 × 10^6^ cells/mL and plated. T cells were cultured in RPMI-1640 medium supplemented with 10% FBS, 100 U/mL penicillin, 100 μg/mL streptomycin and 50 U/mL IL-2 (BioLegend, 575402) on plates pretreated with anti-CD3e/CD28 mAbs (1 μg/mL) (Invitrogen, 16-0031-85) in the presence of soluble anti-CD28 antibody (2 μg/mL) (BioLegend, 102102). Cells were harvested at different time points for CFSE peak analysis by flow cytometry. Control cultures of CFSE-labeled but unstimulated cells, harvested on the same day, were used as a reference for the fluorescence intensity of undivided T cells.

*Apoptosis assay:* T cells were washed with PBS, resuspended in 100 μL cold binding buffer, and stained with fluorescein isothiocyanate (FITC)-Annexin V and propidium iodide (PI) using TACS Annexin V kits (R&D Systems, Minneapolis, MN). 400 μL of 1X binding buffer was added to each sample, and the samples were processed on the MACSQuant Analyzer (Miltenyi Biotec, Bergisch Gladbach, Germany) within 1 hour to obtain the maximum signal.

*CFSE-based in vitro cytotoxicity assay:* CD8^+^ T cells were isolated as described above, activated with plate-bound anti-CD3e/CD28 mAbs (1 μg/mL) (Invitrogen, 16-0031-85) and soluble anti-CD28 monoclonal antibody (2 μg/mL) (BioLegend, 102102), and cultured in RPMI-1640 medium in 10% fetal bovine serum (Invitrogen) and 1% penicillin/streptomycin with 50 U/mL of IL-2 (BioLegend, 575402). Target cells (4T1 cells) were resuspended at 1 × 10^6^ cells/mL in PBS and labeled with 5 μM CFSE (CellTrace Cell Proliferation Kits, ThermoFisher Scientific Inc.) for 20 minutes at 37°C. The reaction was stopped by the addition of 5 volumes of RPMI-1640 with 10% FBS, followed by 5 minutes of incubation at room temperature. After 2 washes, the CFSE-labeled 4T1 cells were plated in plates, and the cell concentration was adjusted to 1 × 10^5^ cells/mL. CD8^+^ T cells were added at a 10:1 effector-target ratio. Cells were harvested after 48 hours, and quantitative analysis of the cell populations was performed by flow cytometry. 7AAD (BioLegend) was added to stain dead cells. The cell populations were as follows: dead target cells (CFSE^+^7AAD^+^), live target cells (CFSE^+^7AAD^-^), dead effector cells (CFSE^-^7AAD^+^), live effector cells (CFSE^-^7AAD^-^).

*ELISA:* Cell culture supernatants were collected and stored at -20°C before use. Cytokine levels were determined by ELISA according to the manufacturer’s instructions (R&D Systems). Data were collected using a microplate reader (SH-1000 Lab, CORONA ELECTRIC, Japan). Secreted IFN-γ and GZMB were normalized to the number of viable cells measured under identical conditions and expressed per 10⁶ viable cells.

**A-Seq clustering, cell type annotation and analyses**

The resulting gene-by-cell matrices from CellRanger were transformed into Seurat objects (Seurat v4.0.3), and downstream analysis was conducted in R v4.5.0 unless otherwise stated. Solo software was used to remove doublet cells. Moreover, we filtered out cells with less than 200 genes per cell and more than 10% mitochondrial read content. The remaining cells were normalized and scaled using the SCTransform wrapper in Seurat.

The downstream analysis steps include normalization/scaling based on the number of unique molecular identifiers using SCTransform (v2) algorithm, identification of highly variable genes across single cells, scaling based on the number of UMI, dimensionality reduction (PCA and UMAP), unsupervised clustering, and discovery of differentially expressed cell type-specific markers. The main cell types were annotated based on the expression of established marker genes, which were further refined manually using established literature (*CD3D/CD3E/CD4/CD8A/CD8B* for the T cells, *CD14/ITGAX/HLA-DRA/CD68/ITGAM* for myeloid cells, *MS4A1/CD19* for B cells, *MS4A2* for mast cells, and *IGHG1/IGKC* for plasma cells) (Shiao et al., 2024; Tietscher et al., 2023). No large-scale batch effects derived from individual patients, sequencing runs, or immune environments were found upon visual inspection of the UMAP. Differentially expressed genes (DEGs) were identified using the nonparametric Wilcoxon rank-sum test, with FDR < 0.05, and |log2FC| > 0.5.

Patient-level (pseudobulk) comparison. To evaluate the difference in SPRED2 expression between responders and non-responders using the patient as the unit of analysis, a pseudobulk value was computed for each patient as the mean normalized SPRED2 expression (Seurat RNA assay, data slot) across all T cells from that patient. The resulting values (responders, n = 5; non-responders, n = 5) were compared with a two-sided Wilcoxon rank-sum test. The per-cell violin plot in Figure 7E is shown for descriptive purposes only; because cells from the same patient are not independent observations, no statistical test was applied at the cell level.

To define the *SPRED2^high^* T-cell signature, T cells were first extracted from the scRNA-seq datasets of patients with TNBC. Cells were then stratified into *SPRED2^low^* and *SPRED2^high^* T cells subpopulations based on the *SPRED2* expression (mean). DEGs between these two groups were identified, followed by filtering out genes that were not specifically enriched in T cells. Only genes with preferential expression in T cells compared to other cell types were retained, resulting in a final set of 12 *SPRED2^high^* T-cell signature genes. To estimate the presence of *SPRED2^high^* T cells in bulk RNA-seq data, we used the UCSC Xena database to calculate the average expression of the 12 signature genes and used it as the *SPRED2^high^* T-cell signature score. The gene list for this signature is provided in the Supporting Information (Table S2).

**Bioinformatic analysis of bulk RNA-seq data**

Public bulk RNA-seq data were used for the analysis of gene expression in human and mouse CD8^+^ T cells. After quality control (QC) and trimming with Trim Galore v0.6.10, reads were aligned with STAR v2.7.10b to the GRCh38 genome for human datasets and to the GRCm39 genome for mouse datasets (Dobin et al., 2013). Transcriptome assembly was conducted de novo with StringTie v2.2.1, guided by ENSEMBL annotations. Assembled transcripts were merged into a unified reference and re-annotated with SQANTI3 v5.0 for structural quality control (Tardaguila et al., 2018; Shumate et al., 2022). All samples were then re-quantified at the transcript level using Salmon v1.9.0 (Patro et al., 2017), and additional quality filtering was performed using IsoformSwitchAnalyzeR v1.17.05 based on the mapping results from Salmon (Vitting-Seerup and Sandelin, 2019).

After batch correction with Removal of Unwanted Variation from RNA-seq data (RUVSeq) (Risso et al., 2014), the differentially expressed genes (DEGs) were detected with DESeq2 v1.31.16 (Love et al., 2014). Significant DEGs were defined as False Discovery Rate (FDR) < 0.05 and |log_2_FC| > 1. Over-Representation Analysis (ORA) was conducted using the Gene Ontology (GO) databases and Kyoto Encyclopedia of Genes and Genomes (KEGG) database by clusterProfiler v4.6.0 (Wu et al., 2021).

To generate Fig. 6B and Fig. 7A, for each dataset, gene expression was extracted from the count matrices and scaled to a minimal value of 0 and a maximal value of 1 to allow for inter-gene comparison.

**Dynamic gene expression trend analyses**

Gene expression dynamics over time were evaluated for human CD8 T cells. Genes with all-zero counts or low expression ( counts < 10 and ≤ 2 samples) were removed. Housekeeping and Reference Transcript Atlas were excluded. Size factors were computed using MedianNorm, and following normalization procedures, gene expressional paths over time were identified for each severity group through time-series differential analysis via normalized counts were analyzed with EBSeq-HMM v1.5.2 across ordered time points (Leng et al., 2015).

Briefly, EBSeq-HMM utilizes an autoregressive hidden Markov model to categorize expressional dependence over ordered conditions (time points 0h, 12h, 24h, 72h and 7d in the case of this study) and identifies dynamic paths where gene expression continuously changes. For this study, we specifically retained genes possessing continuous changes (up or downregulation) between each time point, with the 16 pathways described. EBSeq-HMM was performed with 50 iterations of the Baum-Welsh algorithm, an expected fold-change of 2.0, two-chain mixture proportion updating set to FALSE , and all other parameters set to default. Dynamically expressed genes were retained, having a posterior probability ≥ 0.499 and FDR < 0.01.

**Analysis of transcriptomic data using online databases**

We performed multi-omics analyses of TCGA-BRCA using the LinkedOmics database (https://www.linkedomics.org/login.php). The significance threshold for Spearman correlation testing was set at p ≤ 0.05. Pearson correlation coefficients were used to quantify *SPRED2*-associated genes in the BRCA cohort, and results were displayed as volcano plots (false discovery rate, FDR ≤ 0.05). Over-representation analysis (ORA) was conducted with the LinkInterpreter module (P < 0.05). Cross-cohort correlation comparisons and meta-analysis were performed with the LinkCompare module.

We used the TIMER 3.0 database (https://compbio.cn/timer3/) to analyze potential correlations between *SPRED2* and the expression of multiple T cell related genes infiltrating BRCA. Partial Spearman correlation adjusted for tumor purity was applied, with purity as a covariate and rank-based partial correlations computed. Cross-database validation was performed using TNMplot (https://tnmplot.com/analysis/).

UCSC Xena (https://xena.ucsc.edu/) was used to assess differential expression of the *SPRED2^high^* T cell signature between adjacent and normal tissues and across molecular subtypes within the TCGA-BRCA dataset. UALCAN (<https://ualcan.path.uab.edu/index.html>) was used to assess differential expression of *SPRED2* mRNA between adjacent and normal tissues and across molecular subtypes within TCGA-BRCA.

**Protein-Protein Interaction (PPI) Network, clustering, and functional enrichment**

We constructed the PPI network using the genes from the target time-series cluster that showed significant functional enrichment. The PPI network was generated using the STRING database (Version: 12.0, <https://cn.string-db.org/>) (interaction score ≥ 0.40) and visualized in Cytoscape (v3.10.4). Network modules were further identified using the Markov Cluster Algorithm (MCL) plugin (inflation parameter = 3), and functional enrichment analysis of the modules was performed with the ClueGO plugin.

**Over-representation analysis (ORA) of DEGs**

ShinyGO 0.77 performed ORA to identify significantly enriched biological processes and molecular functions underlying DEGs. Functional annotation queried Gene Ontology (GO), Kyoto Encyclopedia of Genes and Genomes (KEGG), and the Reactome pathway database. Statistical significance was evaluated using the Benjamini–Hochberg method (FDR) (p-val.adj < 0.05).

**Gene Set Enrichment Analysis (GSEA)**

Gene sets were obtained from the Molecular Signatures Database (MSigDB) (<https://www.gsea-msigdb.org/gsea/msigdb/>). GSEA was performed using the GSEA software (Broad Institute, <https://www.broadinstitute.org/gsea/>). Pathways with |normalized enrichment score (NES)| > 1, P < 0.05, and FDR < 0.05 were regarded as significantly enriched.

**Survival prognosis analysis**

Overall survival was analyzed in the Sweden Cancerome Analysis Network—Breast (SCAN-B) cohort (NCBI Gene Expression Omnibus, GSE96058; Brueffer et al., JCO Precis Oncol 2018). Of 3,273 unique primary tumors in the series (technical replicates excluded), the 2,976 with available overall-survival follow-up were analyzed (2,969 on GPL11154, 7 on GPL18573). This set corresponds to the breast cancer cohort served by the Kaplan–Meier Plotter resource (<http://kmplot.com/analysis/>) for this series, and our whole-cohort analysis reproduced the values obtained through that interface exactly (n = 1,488 vs 1,488; log-rank P = 0.581; HR 1.06, 95% CI 0.85–1.33). Median follow-up, estimated by the reverse Kaplan–Meier method, was 56.3 months (95% CI 55.4–57.1; IQR 44.2–68.3; range 1.9–82.5 months), and 311 deaths were recorded. AJCC stage is not available for this cohort and was neither imputed nor included in any model; tumor size and nodal status, its principal components, were included as separate covariates.

Overall survival was estimated by the Kaplan–Meier method and compared with the log-rank test. *SPRED2* and the *SPRED2^high^* T-cell signature were dichotomized at the median of each analyzed cohort, with ties assigned to the high arm; cut-offs were re-derived within each subgroup rather than carried over from the whole cohort, and automatic selection of an optimal cut-off was not used. Hazard ratios (HR) with 95% confidence intervals were obtained from Cox proportional-hazards models. Multivariable models were adjusted for age, tumor size, nodal status, histological grade and PAM50 subtype, and *SPRED2* was modelled both as a continuous variable (per 1 SD, fully standardized including mean-centering) and as a median dichotomy. The primary analysis was the interaction model. Interaction P values were based on Wald tests throughout. The proportional-hazards assumption was assessed by Schoenfeld residual tests; age violated the assumption, and models were therefore refitted stratified by age tertile, with unchanged conclusions. Survival and prognostic analyses were performed in Python 3.13 with lifelines 0.30.3.
